# Genome-Wide Functional Profiling Identifies Genes and Processes Important for Zinc-Limited Growth of *Saccharomyces cerevisiae*

**DOI:** 10.1371/journal.pgen.1002699

**Published:** 2012-06-07

**Authors:** Matthew North, Janet Steffen, Alex V. Loguinov, Ginelle R. Zimmerman, Chris D. Vulpe, David J. Eide

**Affiliations:** 1 Department of Nutritional Science and Toxicology, University of California Berkeley, Berkeley, California, United States of America; 2 Department of Nutritional Sciences, University of Wisconsin–Madison, Madison, Wisconsin, United States of America; National Institute of Diabetes and Digestive and Kidney Diseases, United States of America

## Abstract

Zinc is an essential nutrient because it is a required cofactor for many enzymes and transcription factors. To discover genes and processes in yeast that are required for growth when zinc is limiting, we used genome-wide functional profiling. Mixed pools of ∼4,600 deletion mutants were inoculated into zinc-replete and zinc-limiting media. These cells were grown for several generations, and the prevalence of each mutant in the pool was then determined by microarray analysis. As a result, we identified more than 400 different genes required for optimal growth under zinc-limiting conditions. Among these were several targets of the Zap1 zinc-responsive transcription factor. Their importance is consistent with their up-regulation by Zap1 in low zinc. We also identified genes that implicate Zap1-independent processes as important. These include endoplasmic reticulum function, oxidative stress resistance, vesicular trafficking, peroxisome biogenesis, and chromatin modification. Our studies also indicated the critical role of macroautophagy in low zinc growth. Finally, as a result of our analysis, we discovered a previously unknown role for the *ICE2* gene in maintaining ER zinc homeostasis. Thus, functional profiling has provided many new insights into genes and processes that are needed for cells to thrive under the stress of zinc deficiency.

## Author Summary

Zinc is needed for the growth of all organisms because it acts as a required cofactor for many different proteins. Zinc deficiency is a common problem faced by free-living microbes, as well as plants and animals including humans. Among bacterial and fungal pathogens, zinc deficiency is also a key problem they can encounter during pathogenesis. To identify genes and processes that are important for growth when zinc is scarce, we used genome-wide functional profiling. In this approach, a collection of ∼4,600 mutant yeast strains, each lacking the function of a different gene, was tested to determine which genes are needed for optimal growth in low zinc. More than 400 genes were identified as being important. The identity of these genes implicates a large number of different processes as critical for low zinc growth. These included genes that are targets of the zinc-regulated Zap1 transcription factor as well as genes involved in secretory pathway function, oxidative stress resistance, vesicular trafficking, peroxisome biogenesis, and chromatin modification. As a result, we now know of many processes that might be good targets for the development of new antifungal drugs.

## Introduction

Zinc is an essential nutrient for all organisms because it is required as a catalytic and/or structural cofactor for hundreds of proteins [1]. In addition, recent evidence suggests that zinc may serve as an important secondary messenger in various signal transduction pathways [2], [3]. As a cofactor for C_2_H_2_ zinc finger proteins and other zinc-binding transcription factors, zinc is also required for transcriptional regulation of many genes [4]. Given these many roles, cells have evolved with efficient mechanisms to maintain intracellular zinc homeostasis and growth when extracellular zinc levels become limiting.

Zinc deficiency is a common problem faced by free-living microbes as well as plants and animals including humans. Among bacterial and fungal pathogens, zinc deficiency is also a key problem they can encounter during pathogenesis. The innate immune system is an important first line of defense against infection. One strategy of innate immunity has been referred to as “nutritional immunity”, i.e. the withholding of essential nutrients such as iron from the invading pathogen [5]. By preventing the pathogen from obtaining essential nutrients, the host limits the growth of microbes and sensitizes them to killing by other agents, e.g. reactive oxygen species. Withholding zinc from microbes is also an important mechanism of nutritional immunity. Up-regulation of the liver Zip14 zinc transporter during infection decreases plasma zinc levels [6]. In addition, neutrophils produce high levels of the S100A8/S100A9 protein dimer called “calprotectin” [7]. Calprotectin is released from neutrophils and binds zinc and other metals to inhibit microbial growth [8]–[12]. Thus, strategies used by cells to grow under zinc-limiting conditions are important for both microbial physiology and human health.

To address the question of what genes and processes are important for growth of a zinc-limited yeast cell, we and others have used genome-wide transcription profiling to identify genes with altered expression in response to changes in zinc status [13]–[16]. In our studies, zinc-deficiency was found to induce expression of over 400 genes [13], [14]. Among these are approximately 80 genes that are direct targets of the Zap1 transcription factor. Zap1 is a zinc-responsive activator protein that increases gene expression in zinc-limited cells [17]. Identification of Zap1-regulated genes has led to a number of insights into how cells adapt to zinc deficiency. Among Zap1 targets is the *ZAP1* gene itself. Up-regulation of its own transcription is likely to enhance the overall transcriptional response, especially for genes that have low affinity Zap1 binding sites in their promoters [14]. Zap1 also up-regulates the *ZRT1*, *ZRT2*, and *FET4* genes that encode zinc transporters needed for zinc uptake [18]–[20]. Zap1 increases *ZRT3* expression because this gene encodes a transporter responsible for mobilizing zinc stored in the yeast vacuole [21]. As a final example, Zap1 increases expression of the *TSA1* gene. Tsa1, the major peroxiredoxin in the cytosol of cells, degrades hydrogen peroxide [22]. This and other reactive oxygen species increase in zinc-limited cells and Tsa1 is required to combat that oxidative stress. Among Zap1-independent transcriptional effects, we discovered that the unfolded protein response, a response to disruption of processes in the endoplasmic reticulum, is also induced in low zinc and this led to the recognition that ER function requires zinc [23].

As these discoveries demonstrate, transcriptomics analysis of gene expression has told us many things about how cells respond and adapt to zinc-limiting conditions. While useful, however, this approach is limited by the fact that it requires a gene to change in expression in order to be identified as potentially important. Many proteins needed for low zinc growth are likely to be constitutively expressed or regulated at post-transcriptional levels. A complementary approach that does not have this limitation is genome-wide functional profiling [24], [25]. In this approach, a collection of haploid or homozygous diploid deletion strains, each disrupted for function of a different gene, is used to directly assess which genes are important for optimal growth in a particular environment. In addition, mutations that improve growth in that condition can also be identified. We have used this approach to identify genes that are required for zinc-limited growth. Our findings indicate that a large number of different processes are important for optimal growth under these conditions. These include genes that are targets of the Zap1 transcription factor as well as genes involved in endoplasmic reticulum function, oxidative stress resistance, vesicular trafficking, peroxisome biogenesis, and chromatin modification.

## Results

### A genome-wide screen for mutants altered for low zinc growth

**Figure 1:**
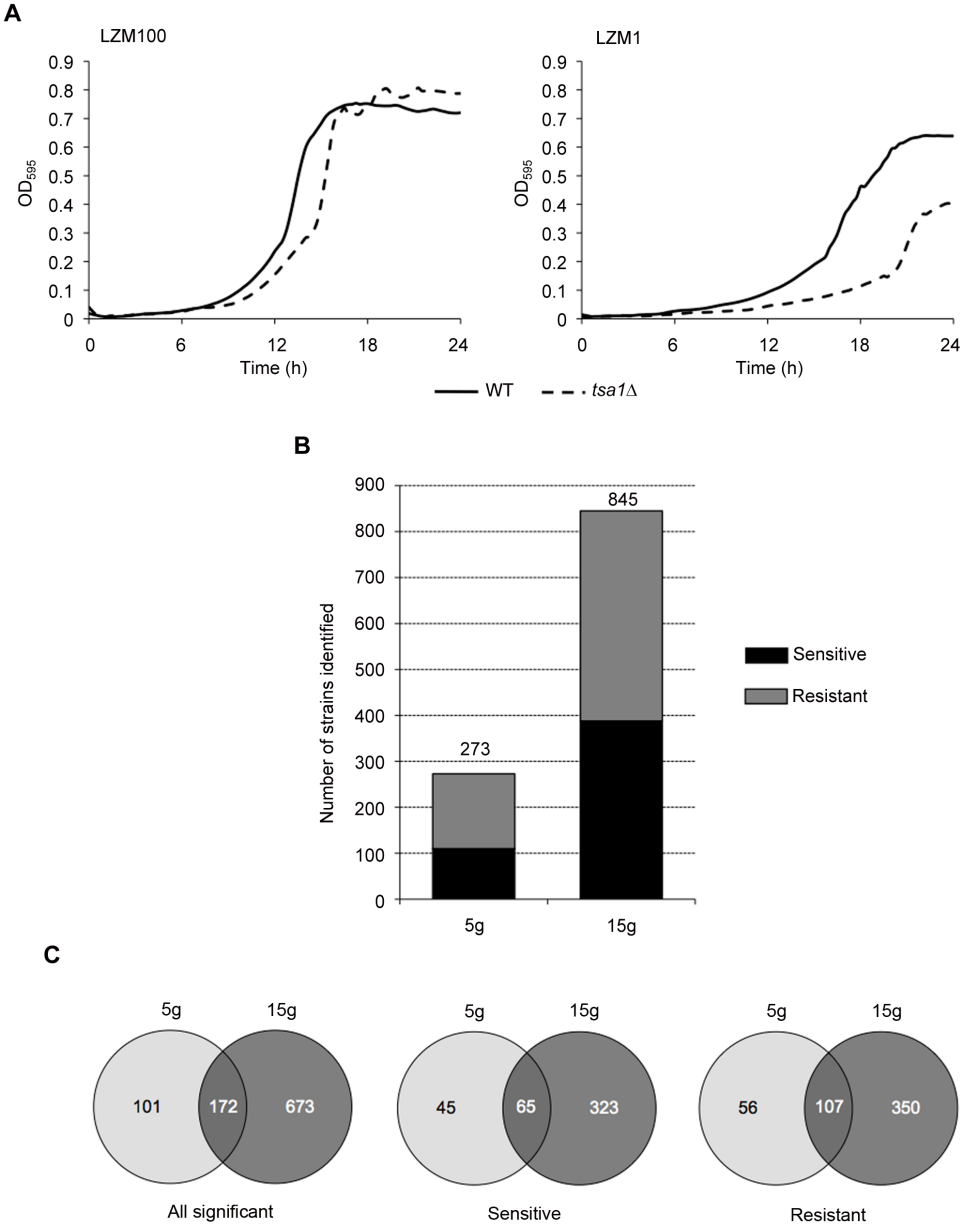
Functional profiling analysis. A) Zinc limitation in LZM+1 µM ZnCl_2_ (LZM1) results in decreased growth of wild-type cells relative to replete LZM+100 µM ZnCl_2_ (LZM100). A *tsa1*Δ mutant shows increased sensitivity to limiting zinc relative to wild type. B) Numbers of low zinc sensitive and resistant deletion strains identified by differential strain sensitivity analysis (DSSA). The number of significantly affected strains identified was greater after more generations of growth. C) Venn diagrams showing the number of genes whose mutants showed growth effects after five and fifteen generations. The degree of overlap is also indicated.

To identify genes important for low zinc growth, cells were grown in either zinc-limiting (LZM+1 µM ZnCl_2_) or zinc-replete (LZM+100 µM ZnCl_2_) media. LZM allows for control of the zinc available to cells due to the high concentrations of EDTA (1 mM) and citrate (20 mM) serving as a metal buffers in that medium. Wild-type cells grew markedly better in LZM+100 µM ZnCl_2_ [area under curve (AUC) value = 34.7] than did cells grown in LZM+1 µM ZnCl_2_ (AUC = 21.0) (Figure 1A). Thus, cells grown in LZM+1 µM ZnCl_2_ are zinc limited. Supplementation of 100 µM Zn was chosen as the replete condition because it provides sufficient zinc for maximal growth but does not exceed the metal buffering capacity of the medium and alter the availability of other metals such as Fe and Cu [18]. As an illustration of defective growth in low zinc, we included a *tsa1Δ* mutant in this experiment. *TSA1* encodes the major cytosolic peroxiredoxin in yeast and is required for combating the oxidative stress encountered during low zinc growth [22]. As shown in Figure 1A, the *tsa1Δ* mutant grew almost as well as the wild-type strain in LZM+100 µM ZnCl_2_ but very poorly in LZM+1 µM ZnCl_2_. Our previous studies demonstrated that adding other metals to 100 µM concentrations did not increase growth rate in LZM+1 µM Zn of either wild type or *tsa1Δ* mutants indicating that these cells are specifically limited for zinc [22].

To perform genome-wide functional profiling, mixed populations of 4607 different viable deletion mutants were inoculated into zinc-limiting and zinc-replete media. The cells were grown for either five or fifteen generations, genomic DNA was isolated from the populations, and the unique “barcode” oligonucleotides that identify each mutant in the deletion collection were amplified by PCR. These PCR products were then labeled and hybridized to Affymetrix TAG4 microarrays containing oligonucleotides complementary to the barcode sequences (see Materials and Methods). Four replicate cultures were used for each condition of zinc status and generation number.

Mutations in many genes were found to affect low zinc growth. After five generations, 110 mutants showed a reproducible growth defect in low zinc and were designated “low zinc sensitive”. Another 163 mutants were apparently resistant to low zinc and showed greater prevalence in the low zinc cultures than they did in the zinc-replete medium (Figure 1B). After fifteen generations, a total of 388 mutants were identified as sensitive to low zinc while another 457 mutants showed apparent low zinc resistance. The results for all mutant strains with altered growth are provided in Tables S1 and S2. A list of all strains tested and found to have no significant growth alteration (n = 3663) is provided in Table S3. It is important to note that genes required for growth in low iron (e.g. *FET3*, *FTR1*), copper (e.g. *CTR1*, *MAC1*) or manganese (e.g. *SMF1*) did not show a growth defect in low zinc relative to zinc-replete conditions confirming that the growth medium used in these experiments was specifically controlling zinc availability and the availability of other metals was not significantly affected. As shown in Figure 1C, there was a high degree of overlap between the mutants found to have altered growth after five generations versus fifteen generations. We will first consider those mutants showing a growth defect in low zinc. Surprisingly, 45 low zinc sensitive strains were detected after five generations that were not found in the fifteen generation data set. Their absence may be due to false negative effects (e.g. *icy2Δ*, *tsa1Δ*) or, alternatively, initially slow growing strains may have accelerated growth in later generations.

### Confirmation of the low zinc growth defect by competitive growth assays

**Figure 2:**
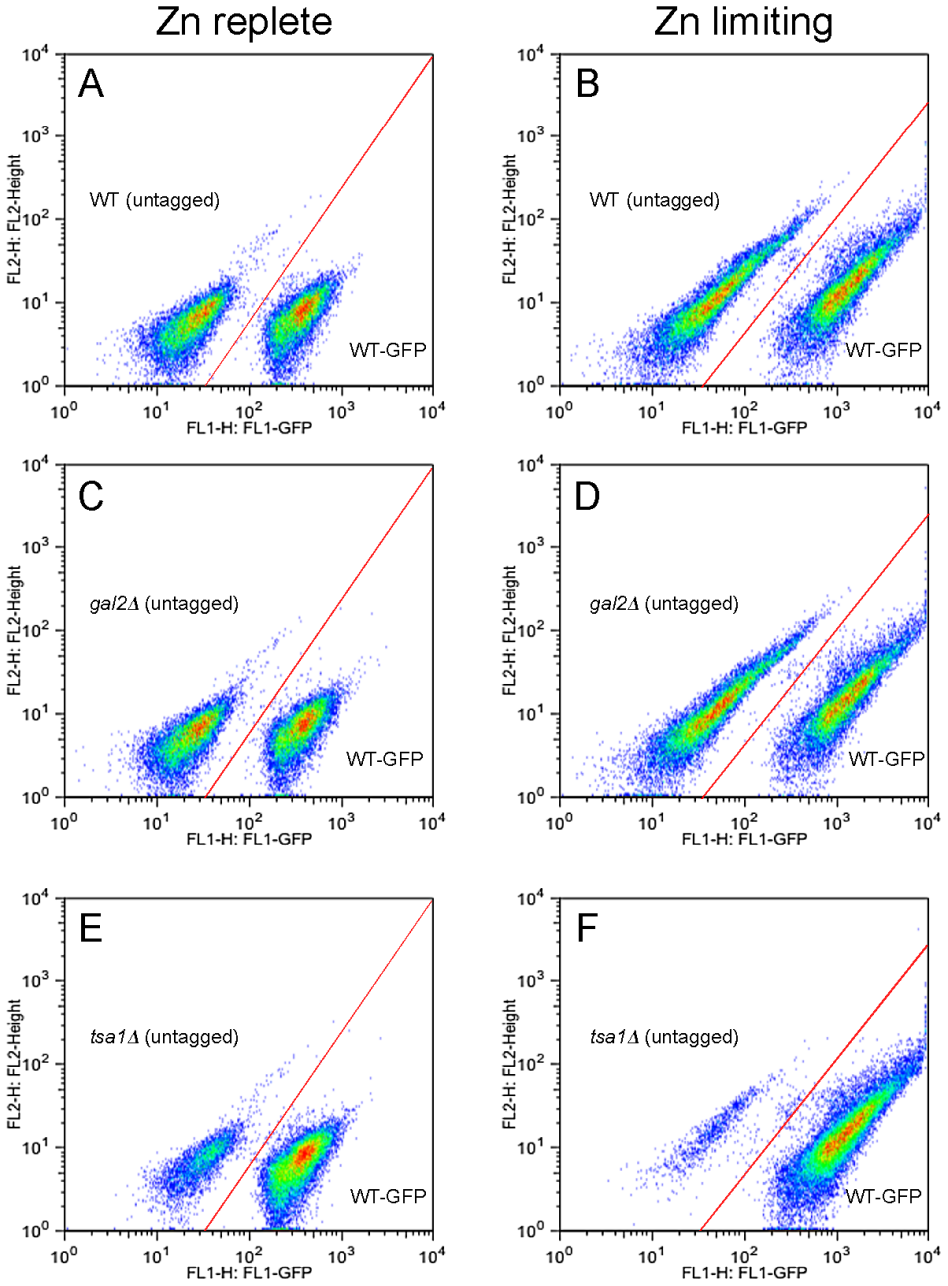
Analysis of low zinc growth by flow cytometry. Untagged wild-type BY4743 (panels A, B), *gal2Δ* (panels C, D), and *tsa1Δ* (panels E, F) cells were mixed with approximately equal numbers of GFP-expressing BY4743 cells and inoculated into zinc-replete (LZM+100 µM ZnCl_2_, panels A, C, E) or zinc-limiting (LZM+1 µM ZnCl_2_, panels B, D, F) media and grown for fifteen generations prior to analysis by flow cytometry. Approximately 20,000 total cells per culture were assessed for GFP fluorescence (x-axis) and autofluorescence (y-axis). The *red line* in each panel marks the boundary between the sub-populations of tagged and untagged cells. Quantitation of these data is provided in Table 1. The elongated distribution of fluorescence in zinc-limited cells is likely due to alterations in cell size and cell wall composition relative to zinc-replete cells and was observed for both GFP fluorescence and autofluorescence.

To confirm these results for selected mutants, we used an independent and sensitive assay of growth using flow cytometry. The wild-type strain (BY4743) was transformed with an integrating plasmid vector that expressed GFP from a strong promoter [26]. GFP-expressing cells can be readily distinguished from untagged cells by flow cytometry. To compare growth of a GFP-tagged wild-type strain with an untagged mutant, the two strains were mixed together with approximately equal numbers of cells and then inoculated into low zinc or zinc-replete media. After fifteen generations, the level of each strain was assessed by counting ∼20,000 total cells per culture. As shown in Figure 2A and 2B, GFP-tagged and untagged wild-type cells grew equally well in both low zinc and zinc-replete conditions. Quantitation of the results from triplicate cultures is provided in Table 1. As an additional control, we tested growth of an untagged *gal2Δ* mutant. *GAL2* encodes galactose permease and is not predicted to be required for growth in the medium used here where glucose is the carbon source. No growth defect was observed (Figure 2C and D, Table 1) indicating that the antibiotic resistance marker (kanMX4) used to generate the deletion mutants in the collection does not alter growth in low zinc or zinc-replete media. In contrast, while the untagged *tsa1Δ* mutant grew fairly well in the zinc-replete medium and was found at ∼28% of the final population after 15 generations, it was almost completely out-competed by the wild-type strain in low zinc cultures (0.6%, Figure 2E versus Figure 2F, Table 1).

**Table 1:** Confirmation of functional genomics analysis results by flow cytometry.

Strain	% of mutant in initial inoculum	% of mutant after 15 gen. in 1 µM ZnCl_2_	% of mutant after 15 gen. in 100 µM ZnCl_2_	−Zn/+Zn ratio	p-value^a^
**Control strains**					
BY4743	52.30	53.43±0.36	52.17±0.29	1.0	NS
*gal2Δ*	49.10	51.53±0.23	50.50±0.62	1.0	NS
**Zap1 target genes**					
*tsa1Δ*	45.72	0.62±0.16	27.89±0.19	0.02	0.00004
*icy2Δ*	48.79	1.21±0.45	55.22±0.64	0.02	0.0001
*adh4Δ*	50.62	38.77±3.24	53.73±0.38	0.7	0.01
*uth1Δ*	47.35	18.85±0.75	36.46±0.79	0.5	0.0009
*hnt1Δ*	49.70	30.00±1.30	47.30±0.30	0.6	0.002
*zrg17Δ*	51.01	16.00±0.33	53.73±0.34	0.3	0.0
**ER function**					
*ire1Δ*	50.00	3.84±0.22	46.60±0.66	0.08	0.00007
*scj1Δ*	45.10	10.31±0.48	30.87±0.42	0.3	0.0001
*erd1Δ*	27.70	6.43±0.09	9.30±0.55	0.7	0.01
**ROS resistance**					
*yap1Δ*	51.10	29.03±0.40	65.77±0.55	0.4	0.00004
*skn7Δ*	48.30	29.40±0.46	56.43±0.42	0.5	0.0002
**Peroxisome biogenesis**					
*pex6Δ*	47.00	5.66±0.14	23.13±0.86	0.2	0.0007
*pex10Δ*	47.20	5.59±0.26	14.97±0.59	0.4	0.003
**Histone deacetylation**					
*sap30Δ*	46.80	3.91±0.24	6.42±0.34	0.6	0.007
*sif2Δ*	48.20	16.97±0.35	37.17±0.76	0.4	0.0006

a Significance was defined as having a p-value less than 0.05 comparing the mutant prevalence after 15 generations growth in low versus high zinc; NS = not significant.

Using this method, we confirmed the results for fifteen of the mutants that showed poor low zinc growth in the functional profiling analysis (Table 1). Several of the mutants (e.g. *tsa1Δ*, *uth1Δ*, *scj1Δ*, *erd1Δ*) showed decreased competitive fitness relative to wild type in zinc-replete media but that growth defect was exacerbated by low zinc conditions. Independent confirmation of the low zinc growth defects of these strains indicated that the functional profiling results were very reliable.

### Functional enrichment and network mapping implicate a limited set of cellular processes and components as required for optimal low zinc growth

We analyzed the low zinc sensitive set of 433 genes identified in the five and fifteen generation experiments (Table S1) for enrichment of biological attributes by identifying significantly overrepresented GO (Gene Ontology) and MIPS (Munich Information Center for Protein Sequences) categories (Table 2). A number of categories were found to be enriched, including autophagy (GO category GO:0006914), the endosome (GO:0005768), vacuolar/lysosomal transport (MIPS category 20.09.13), modification by acetylation/deacetylation (14.07.04) and the peroxisomal membrane (760.01).

**Table 2:** Enrichment among low zinc-sensitive mutants by biological process, cellular component, functional classification, and subcellular localization.

GO Biological Process category	p-value	Genes identified	k^a^	f^b^
Negative regulation of transcription from RNA polymerase II promoter [GO:0000122]	3.15E-07	*SRB8 REG1 ARG82 HDA2 SSN2 YHP1 SPT2 OPI1 IXR1 SPT8 RFX1 MOT3 SSN8 MKS1 RTT106 SIN3 SFL1 SSN3 ROX1 HDA3*	20	85
Protein transport [GO:0015031]	3.37E-07	*ATG8 CCZ1 SEC66 ATG12 APM3 ATG22 GCS1 VPS41 VPS74 SXM1 ERD1 SNX41 VPS52 VPS60 DDI1 BST1 MON1 PEX14 LST7 APL6 HSE1 VPS29 CHS7 APS3 VPS53 SNX4 PEP8 VPS35 ATG27 SOP4 RAV1 VPS55 VPS24 DID2 PEP3 PEX13 NUP2 VPS38 VID22 VAC14 ERV25 COG8 NUP188 ATG16 SCJ1 COG6 ATG3 TLG2 VPS68 ERP4 VPS5 VPS17 VPS30 APL5*	54	412
Autophagy [GO:0006914]	3.42E-06	*ATG8 CCZ1 ATG12 ATG22 ATG15 TRS85 MON1 NVJ1 ATG27 AIM26 UTH1 ATG16 ATG3 VAM3 VPS30*	15	59

GO Cellular Component category	p-value	Genes identified	k^a^	f^b^
Endosome membrane [GO:0010008]	1.37E-07	*SNX41 NHX1 VPS52 VPS60 HSE1 ARN1 VPS53 SNX4 VPS55 VPS24 DID2 VPS38 STV1 VPS27 TLG2 VPS5 VPS30*	17	60
Rpd3L-Expanded complex [GO:0070210]	9.77E-07	*DEP1 SIF2 SNT1 UME6 HOS2 SET3 SAP30 PHO23 SIN3*	9	19
Endosome [GO:0005768]	1.09E-06	*CCZ1 ATG15 GCS1 VPS41 SNX41 NHX1 VPS52 VPS60 MON1 PSD2 HSE1 ARN1 VPS29 VPS53 SNX4 PEP8 VPS35 VPS55 VPS24 DID2 VPS38 STV1 PDR17 VPS27 TLG2 VPS5 VPS17 VPS30*	28	160
Astral microtubule [GO:0000235]	5.16E-06	*SHE1 CIN8 ARP1 LDB18 JNM1 NIP100*	6	9

MIPS Functional Classification category	p-value	Genes identified	k^a^	f^b^
Vacuolar/lysosomal transport [20.09.13]	1.44E-06	*ATG8 CCZ1 ATG12 ATG22 FEN1 VPS74 NHX1 DDI1 MON1 VAM7 LST7 HSE1 VPS29 VPS35 ATG27 VPS55 VPS24 DID2 PEP3 VPS38 STV1 VPS27 ATG3 TLG2 VPS68 VAM3 VPS30*	27	153
Protein targeting, sorting and translocation [14.04]	1.60E-06	*PEX22 ATG8 CCZ1 SEC66 ATG12 ATG22 PEX19 MAF1 PEX3 MSN5 VPS74 ERD1 SNX41 MON1 PEX14 PEX8 HSE1 VPS29 SVP26 PEP8 VPS35 VPS24 DID2 SSA2 PEP3 PEX13 NUP2 VPS38 NUP188 SCJ1 VPS27 ATG3 TLG2 PEX15 SHR5 VPS68 ERP4 VPS5 VPS17 VPS30*	40	281
N-directed glycosylation, deglycosylation [14.07.02.02]	1.86E-06	*ALG3 OST5 DIE2 SVP26 HOC1 VAN1 SCJ1 ALG9 ALG12 ALG6 ALG8 OST3 ALG5*	13	43
Transcriptional control [11.02.03.04]	2.26E-06	*DEP1 HHF1 SIF2 SRB8 SOK1 REG1 STB3 ARG82 UME6 HTA1 MET32 HDA2 SWR1 ESC2 SPT3 SSN2 YHP1 SWI4 SPT2 HAC1 OTU1 HOS2 SPT4 YAP3 OPI1 SNF6 SKN7 CST6 NOT3 HOS4 ASF1 SPT10 IXR1 CNB1 SPT8 BUR2 CDC73 CNA1 YAP1 SOK2 SPT21 SAP30 SSN8 CRZ1 PHO23 LEM3 SIN3 HTZ1 MBF1 TYE7 SSN3 HFI1 HDA3*	53	426
Autoproteolytic processing [14.07.11.01]	2.50E-06	*ATG8 CCZ1 ATG12 ATG22 ATG15 MON1 SNX4 ATG16 ATG3 VPS30*	10	26
DNA conformation modification (e.g. chromatin) [10.01.09.05]	3.33E-06	*HHF1 SIF2 SWC5 DPB3 SNT1 BRE1 PAA1 HTA1 HDA2 SWR1 ESC2 SPT3 HOS2 RSC1 SPT4 SNF6 HOS4 SPT10 ELF1 SET3 SPT8 YKU80 SAP30 YKU70 PHO23 SIN3 HTZ1 SPP1 HFI1 HDA3*	30	188
Transcription repression [11.02.03.04.03]	5.48E-06	*TUP1 MAF1 NRG1 PUF6 RFX1 MOT3 MKS1 SIN4 SFL1 ROX1*	10	28
Modification by acetylation, deacetylation [14.07.04]	5.94E-06	*SIF2 SNT1 NAT1 HDA2 SPT3 HOS2 ARD1 HOS4 SPT10 SET3 SPT8 SAP30 PHO23 SIN3 HFI1 HDA3*	16	69

MIPS Phenotypes category	p-value	Genes identified	k^a^	f^b^
Other vacuolar mutants [52.65.99]	4.92E-05	*VPS41 VAM7 PEP8 CNB1 PEP3 CNA1 VAN1 VPS27 ATG3 VPS5 VAM3 VPS17*	12	49

MIPS Subcellular Localization category	p-value	Genes identified	k^a^	f^b^
Endosome [765]	9.82E-07	*CCZ1 SNX41 NHX1 HSE1 ARN1 SNX4 PEP8 VPS35 VPS55 VPS24 DID2 PEP3 VPS38 VPS27 TLG2 VPS5 VPS17*	17	68
Peroxisomal membrane [760.01]	4.41E-06	*PEX19 PEX10 PEX3 VTC1 PEX14 PEX31 PEX4 PEX13 PEX15*	9	22

a k = number of genes of specific category identified by screen that when deleted increase sensitivity to low zinc.

b f = number of genes in specific GO/MIPS category.

**Figure 3:**
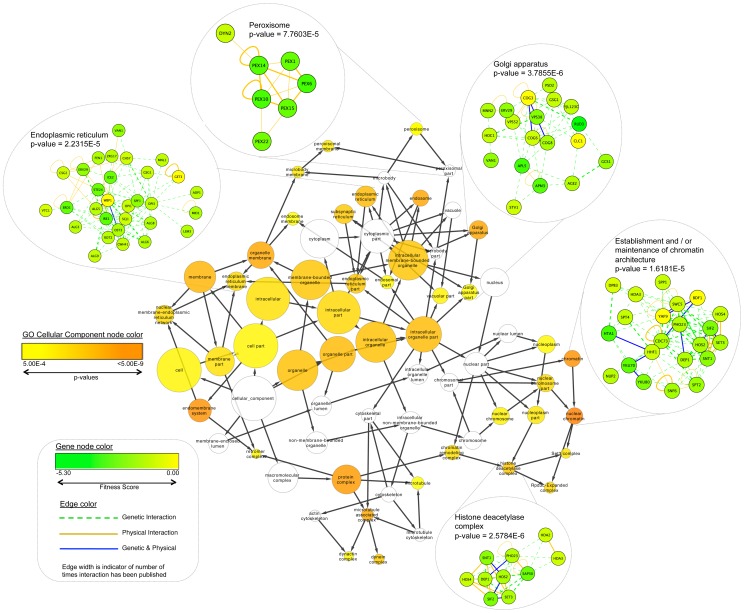
Fitness data for all significantly affected sensitive strains identified from this study were mapped onto the *S. cerevisiae* BioGRID interaction dataset using Cytoscape. The fitness scores (the difference in the mean of the log_2_ hybridization signal between LZM+1 µM ZnCl_2_ and LZM+100 µM ZnCl_2_) of these sensitive strains were then used to identify and create a smaller sub-network (283 genes) containing the sensitive genes and the non-sensitive and essential genes that link them through known genetic and physical interactions. The sub-network was then assessed for significant overrepresentation of Gene Ontology (GO) Cellular Component categories. These categories were visualized as a linked network. Node color of categories indicates the significance of representation (white = not identified as significant) and node size indicates the number of genes identified present in each category. Edge arrows indicate hierarchy of GO terms. For clarity, only GO Cellular Component categories with a p-value<0.0005 are shown. A separate GO enrichment assessment identified overrepresentation of all GO categories in the sub-network. This analysis was used to generate visual representations of the GO processes and cellular components identified showing the genes involved in these processes. In these cases, node color indicates the sensitivity of each deletion strain in our study (fitness score). The edge color defines the interaction type between nodes (from the BioGRID database).

To provide further insight into the biological processes required for the tolerance of zinc limitation, we performed a network mapping analysis. Fitness data for all sensitive strains identified in this study were mapped onto the BioGRID *S. cerevisiae* functional interaction data set. Fitness scores for each mutant, i.e. the difference in the mean of the log_2_ hybridization signal between LZM+1 µM ZnCl_2_ and LZM+100 µM ZnCl_2_, were then used to identify a sub-network within the BioGRID network enriched for genes that have sensitive fitness data associated with them, and so may identify cellular processes or protein complexes affected by zinc limitation. Mutants sensitive to low zinc were then assessed for significant overrepresentation of GO categories, which can be visualized in many different ways (Figure 3). Cellular components identified included the endoplasmic reticulum, the peroxisome, the Golgi apparatus, and histone deacetylase complexes.

### A subset of Zap1 targets are important for low zinc growth

Representative fitness scores from the microarray data for some of the mutants identified as low zinc sensitive are listed in Table 3. A fitness score less than 0 indicates a low zinc growth defect. Mutants disrupted for several Zap1-regulated genes showed poor low zinc growth consistent with their normally induced expression under these conditions. These genes were *TSA1*, *ICY2*, *ADH4*, *UTH1*, *HNT1*, and *ZRG17*.

**Table 3:** Representative mutants showing growth defects in low zinc.

Systematic ORF name	Standard gene name	Fitness score 5 Gen^a^ (log_2_)	Fitness score 15 Gen^b^ (log_2_)	Description
**Zap1 target genes**				
YML028W	*TSA1*	−1.6		Thioredoxin peroxidase required for oxidative stress resistance
YPL250C	*ICY2*	−1.8		Protein of unknown function
YGL256W	*ADH4*		−2.85	Alcohol dehydrogenase isozyme
YKR042W	*UTH1*		−1.65	Implicated in oxidative stress resistance, mitophagy, and cell wall biogenesis
YDL125C	*HNT1*		−1.3	Adenosine 5′-monophosphoramidase of unknown function
YNR039C	*ZRG17*		−1.45	Subunit of the Msc2/Zrg17 endoplasmic reticulum zinc uptake transporter
**Transcriptional response to oxidative stress**				
YML007W	*YAP1*		−1.3	Transcription factor required for oxidative stress tolerance
YIR037W	*HYR1*		−0.95	Thiol peroxidase required for Yap1 function
YBR216C	*YBP1*		−1	Required for Yap1 function
YGL060W	*YBP2*		−0.9	Required for Yap1 function
YHR206W	*SKN7*	−0.6	−1.55	Transcription factor required for oxidative stress tolerance
**ER function**				
YDR205W	*MSC2*		−0.8	Subunit of the Msc2/Zrg17 endoplasmic reticulum zinc uptake transporter
YFL031W	*HAC1*		−2.1	Transcription factor the regulates the Unfolded Protein Response
YHR079C	*IRE1*	−1.45	−3.4	Sensor of ER stress that controls Hac1 activity
YJL073W	*JEM1*		−0.6	DnaJ-like co-chaperone; ER protein folding
YMR214W	*SCJ1*		−1.1	DnaJ-like co-chaperone; ER protein folding
YBL082C	*ALG3*		−1.45	Alpha(1–3) mannosyltransferase; N-linked glycosylation
YPL227C	*ALG5*		−1.4	UDP-glucose:dolichyl-phosphate glucosyltransferase; N-linked glycosylation
YOR002W	*ALG6*		−1.6	Glucosyltransferase; N-linked glycosylation
YOR067C	*ALG8*		−1.65	Glucosyl transferase; N-linked glycosylation
YNL219C	*ALG9*		−1.25	Mannosyltransferase; N-linked glycosylation
YNR030W	*ALG12*		−1.2	Alpha-1,6-mannosyltransferase; N-linked glycosylation
YML115C	*VAN1*		−2.25	Alpha-1,6-mannosyltransferase; N-linked glycosylation
YOR085W	*OST3*	−0.75	−1.7	Oligosaccharyltransferase subunit; N-linked glycosylation
YGL226C-A	*OST5*		−1.1	Oligosaccharyltransferase subunit; N-linked glycosylation
YGR227W	*DIE2*		−1	Dolichyl-phosphoglucose-dependent glucosyltransferase; N-linked glycosylation
YCR044C	*PER1*	−0.85	−1.4	GPI anchor synthesis
YEL031W	*SPF1*	−1.25	−2.35	P-type ATPase involved in ER calcium homeostasis
YIL090W	*ICE2*	−1.1	−3.1	ER membrane protein required for normal ER morphology
YDR414C	*ERD1*		−3.25	Lumenal ER protein retention
YNR051C	*BRE5*		−5.1	Regulates ER-to-Golgi vesicular trafficking
YOR016C	*ERP4*		−0.7	ER-to-Golgi vesicular trafficking
YML012W	*ERV25*		−0.8	ER-to-Golgi vesicular trafficking
YHR181W	*SVP26*		−1	ER-to-Golgi vesicular trafficking
YFL025C	*BST1*		−0.7	ER-to-Golgi vesicular trafficking
**Peroxisome biogenesis**				
YKL197C	*PEX1*		−3.15	AAA ATPase-family peroxin; peroxisomal protein import
YDR329C	*PEX3*		−2.85	Peroxisomal membrane protein; peroxisomal protein import
YGR133W	*PEX4*		−3	Peroxisomal ubiquitin conjugating enzyme; peroxisomal protein import
YNL329C	*PEX6*		−3.9	AAA ATPase-family peroxin; peroxisomal protein import
YGR077C	*PEX8*		−3.5	Peroxisomal protein import
YDR265W	*PEX10*		−3.8	Peroxisomal E3 ubiquitin ligase; peroxisomal protein import
YLR191W	*PEX13*		−2.7	Peroxisomal membrane protein; peroxisomal protein import
YGL153W	*PEX14*		−3.6	Peroxisomal membrane protein; peroxisomal protein import
YOL044W	*PEX15*		−3.2	Peroxisomal protein import system
YDL065C	*PEX19*		−2.9	Chaperone and import receptor, Peroxisomal protein import
YAL055W	*PEX22*		−2.9	Peroxisomal protein import system
YGR004W	*PEX31*		−1.15	Regulator of peroxisome size
**Histone deacetylation**				
YBR103W	*SIF2*	−1.55	−2.7	Set3C histone deacetylase complex subunit
YOL004W	*SIN3*	−1	−1.7	Sin3-Rpd3 histone deacetylase complex subunit
YKR029C	*SET3*	−1	−1.7	SET3 histone deacetylase complex subunit
YGL194C	*HOS2*	−0.9	−1.75	SET3 histone deacetylase complex subunit
YCR033W	*SNT1*	−0.85	−2.4	SET3C histone deacetylase complex subunit
YNL097C	*PHO23*	−0.8	−1.55	Probable Rpd3 histone deacetylase complex subunit
YMR263W	*SAP30*	−0.7	−3.1	Sin3-Rpd3 histone deacetylase complex subunit
YIL112W	*HOS4*		−1.2	SET3 histone deacetylase complex subunit
YDR295C	*HDA2*		−1.05	HDA1 histone deacetylase complex subunit
YPR179C	*HDA3*		−1	HDA1 histone deacetylase complex subunit

Yeast pools were grown in zinc-replete (LZM+100 µM ZnCl_2_) and zinc-limiting (LZM+1 µM ZnCl_2_) media for either five or fifteen generations. The yeast ORFs/genes correspond to representative deletion strains that exhibited a significant change in growth in low zinc when compared to zinc-replete conditions (q<0.05). Numeric values are fitness scores (log_2_ ratios); a negative value indicates a growth defect of the mutant in low zinc. Empty cells in the table indicate that any differences observed were not significant at that generation point.

a Average fitness scores for representative sensitive strains in four replicate experiments after 5 generations of growth.

b Average fitness scores for representative sensitive strains in four replicate experiments after 15 generations of growth.

### Low zinc and oxidative stress

We had also previously shown that zinc-limited yeast cells produce more reactive oxygen species than do replete cells [22]. Induction of *TSA1* by Zap1 is critical for tolerating this increased ROS. Functional profiling suggests that a specific oxidative stress response mechanism is also activated in low zinc. Yap1 and Skn7 are transcription factors that activate antioxidant and heat shock genes in response to oxidative stress [27]. We found that mutants defective for these two factors are defective for low zinc growth (Table 3). In addition, three accessory factors (*HYR1*, *YBP1*, *YBP2*) required for full Yap1 activity were also identified as growing poorly in low zinc [27]–[29].

### Zinc and organellar function

**Figure 4:**
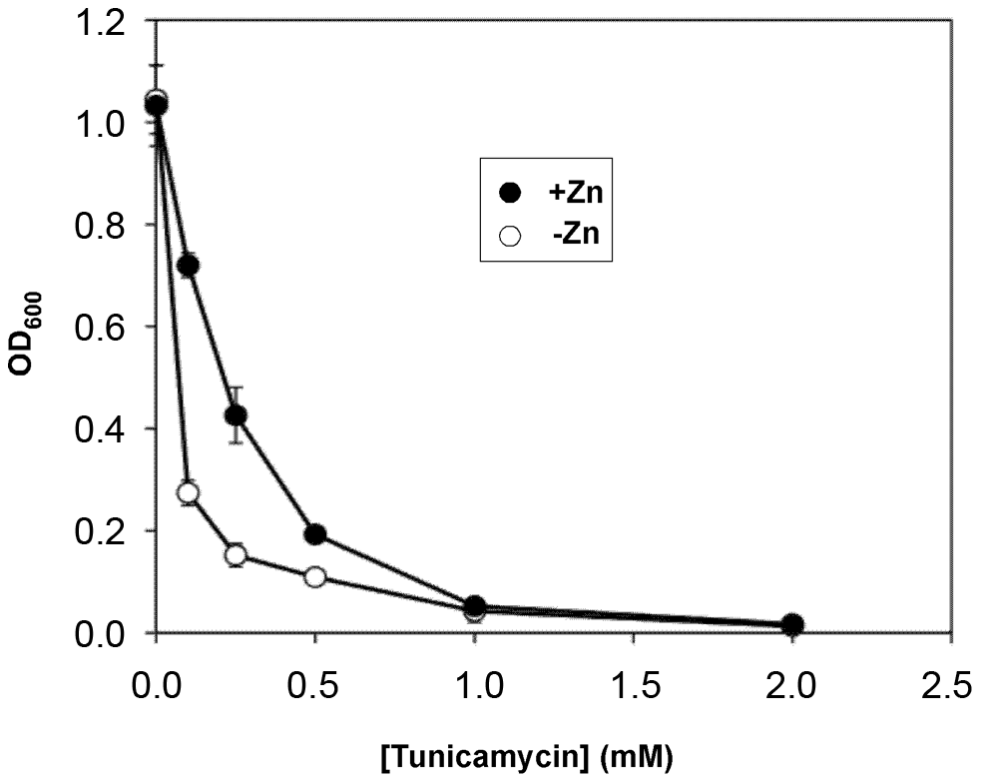
Effects of zinc status on sensitivity to an ER stress inducer. Wild-type (BY4743) cells were inoculated into zinc-replete (LZM+100 µM ZnCl_2_, +Zn) or zinc-limiting (LZM+1 µM ZnCl_2_, −Zn) media containing a range of tunicamycin concentrations and grown overnight prior to measuring the culture optical densities at 600 nm (OD_600_). Hypersensitivity to tunicamycin was observed for zinc-limited cells at 0.1, 0.25, and 0.5 mM tunicamcyin. Data presented are the averages of triplicate cultures for each condition and the error bars indicate ±1 S.D. For most points, the symbols obscure the error bars.

Many genes related to endoplasmic reticulum function were also identified (Table 3). These included *MSC2*, which encodes a zinc transporter protein that, along with Zrg17 (see above), transports zinc into the ER. *HAC1* and *IRE1*, which encode the transcription factor and its controlling sensor that are active in the ER unfolded protein response (UPR), molecular co-chaperones (*JEM1*, *SCJ1*), and several genes involved in ER protein modification and trafficking were identified as growing poorly in low zinc. These results suggested that disruption of ER function in many different ways impairs the ability of cells to grow in low zinc. To test this hypothesis further, we examined the effects of tunicamycin, an inducer of ER stress [30], [31], on low zinc growth. Cells were grown in either low zinc (LZM+1 µM ZnCl_2_) or zinc-replete conditions (LZM+100 µM ZnCl_2_) with a range of tunicamycin concentrations. As shown in Figure 4, sensitivity to tunicamycin was greatly exacerbated by growth in low zinc medium relative to zinc-replete conditions. These results support the hypothesis that unperturbed ER function is critical to growth when zinc is scarce.

Surprisingly, thirteen mutant strains disrupted for peroxisome biogenesis genes were defective for low zinc growth (Table 3). The peroxisome is required for fatty acid β–oxidation in yeast but no fatty acids were supplied as carbon source to these cells. Thus, the importance of the peroxisome in low zinc is unknown.

### Histone deacetylases

The functional enrichment of genes encoding histone deacetylase subunits (i.e. *SIF2*, *SIN3*, *SET3*, *HOS2*, *SNT1*, *PHO23*, *SAP30*, *HOS4*, *HDA2*, and *HDA3*) indicates that chromatin structure plays an important role in low zinc.

### A role for macroautophagy during low zinc growth

Autophagy, the process whereby cells degrade cytosolic proteins and organelles to recycle their components [32] was identified as required for low zinc growth (i.e. “autoproteolytic processing”, Table 2). Autophagy can be subdivided into a number of separate processes including the nonspecific pathway of macroautophagy and the cargo-specific pathways of cytoplasm-to-vacuole targeting (CVT), mitochondria-specific autophagy (mitophagy), and peroxisome-specific autophagy (pexophagy). Several genes involved in both macroautophagy and the cargo-specific pathways, referred to as “core” genes, were found to have a low zinc growth defect by functional profiling; these and additional core autophagy factor mutants (i.e. *atg6Δ*, *atg17Δ*) were confirmed by flow cytometry to have a low zinc growth defect (Table 4). Mutations in cargo-specific factors had much less effect on low zinc growth. For example, *ATG11* is required for both the CVT pathway and pexophagy but the *atg11Δ* mutant grew better in low zinc than in the zinc-replete medium. In addition, mutation of the CVT-specific factor *ATG19* had little effect. Mutation of *MID2* and *SLT2*, which are both required for pexophagy, had a modest effect on low zinc growth when assayed by flow cytometry but these genes are also required for maintenance of cell wall integrity. Thus, their low zinc growth defect may be due to disruption of roles unrelated to pexophagy. Finally, mutations specifically disrupting mitophagy (e.g. *atg32Δ*, *atg33Δ*) had little or no effect on low zinc growth. We conclude from these genetic studies that macroautophagy is required for low zinc growth but the cargo-specific pathways of CVT, pexophagy, and mitophagy are not.

**Table 4:** Macroautophagy is required for zinc-limited growth but not cargo-specific autophagy pathways.

Strain	Fitness score (log_2_)	% of mutant in initial inoculum	% of mutant after 15 gen. in 1 µM ZnCl_2_	% of mutant after 15 gen. in 100 µM ZnCl_2_	−Zn/+Zn ratio	p-value^a^
**Core autophagy factors**						
*atg3Δ*	−1.8	43.50	6.06±0.09	47.93±0.32	0.1	0.00001
*atg6Δ*	ND	43.00	20.80±0.53	26.03±0.06	0.8	0.004
*atg8Δ*	−1.7	41.20	28.73±0.06	46.63±0.15	0.6	0.00001
*atg12Δ*	−0.7	45.80	42.00±0.40	49.40±0.17	0.8	0.003
*atg15Δ*	−4.45	48.50	2.82±0.02	40.10±1.23	0.07	0.0004
*atg16Δ*	−0.7	37.30	16.87±0.51	31.20±0.60	0.5	0.0003
*atg17Δ*	ND	28.70	11.73±0.32	18.23±0.42	0.6	0.007
*atg22Δ*	−0.9	48.20	38.77±0.12	46.37±0.15	0.8	0.0006
*atg27Δ*	−0.85	45.00	36.67±0.45	47.33±0.47	0.8	0.002
**Cargo-specific pathway factors**						
*atg11Δ*	ND	43.20	44.97±0.64	36.37±0.38	1.2	0.0003
*atg19Δ* (CVT)	ND	48.20	48.20±0.44	50.10±0.20	1.0	NS
*mid2Δ* (pexophagy)	ND	47.20	36.67±1.12	38.80±0.36	0.9	NS
*slt2Δ* (pexophagy)	−4.6	37.30	7.68±0.26	9.89±0.21	0.8	0.01
*atg32Δ* (mitophagy)	ND	43.90	65.73±0.46	52.13±0.47	1.3	0.00002
*atg33Δ* (mitophagy)	ND	47.30	45.03±0.71	43.87±0.45	1.0	NS
*fcj1Δ* (mitophagy)	ND	49.40	45.40±0.46	49.93±0.40	0.9	0.007

a Significance was defined as having a p-value less than 0.05 comparing the mutant prevalence after 15 generations growth in low versus high zinc; NS = not significant.

To determine whether macroautophagy is required for growth when other metal ions are limiting, we examined growth of several of the mutants defective for macroautophagy under conditions of iron or copper limitation (Table S4). Iron limitation was induced by adding the iron chelator bathophenanthroline disulfonate (BPS, 100 µM) and copper limitation was imposed by adding the copper chelator bathocuproine disulfonate (BCS, 200 µM) to YPD medium. The four autophagy mutants tested (*atg3Δ*, *atg8Δ*, *atg15Δ*, and *atg16Δ*) showed little or no growth defect in low iron or low copper. In fact, these four mutants showed improved growth in low iron. As a positive control for a low iron growth defect, a *fet3Δ* mutant grew poorly on BPS but not on BCS. *FET3* encodes a component of the high affinity iron uptake system. As a positive control for a defect in low copper, an *irc21Δ* mutant was used and found to grow poorly on BCS but not BPS. *IRC21* encodes a putative cytochrome oxidoreductase and the low copper growth defect of the *irc21Δ* was observed in our previous studies (W. Jo, C. Vulpe, unpublished). When tested in minimal media, the greater importance of autophagy to low zinc growth relative to iron- or copper-limiting conditions was confirmed (Table S5).

### The *ICE2* gene is required for ER zinc homeostasis

**Figure 5:**
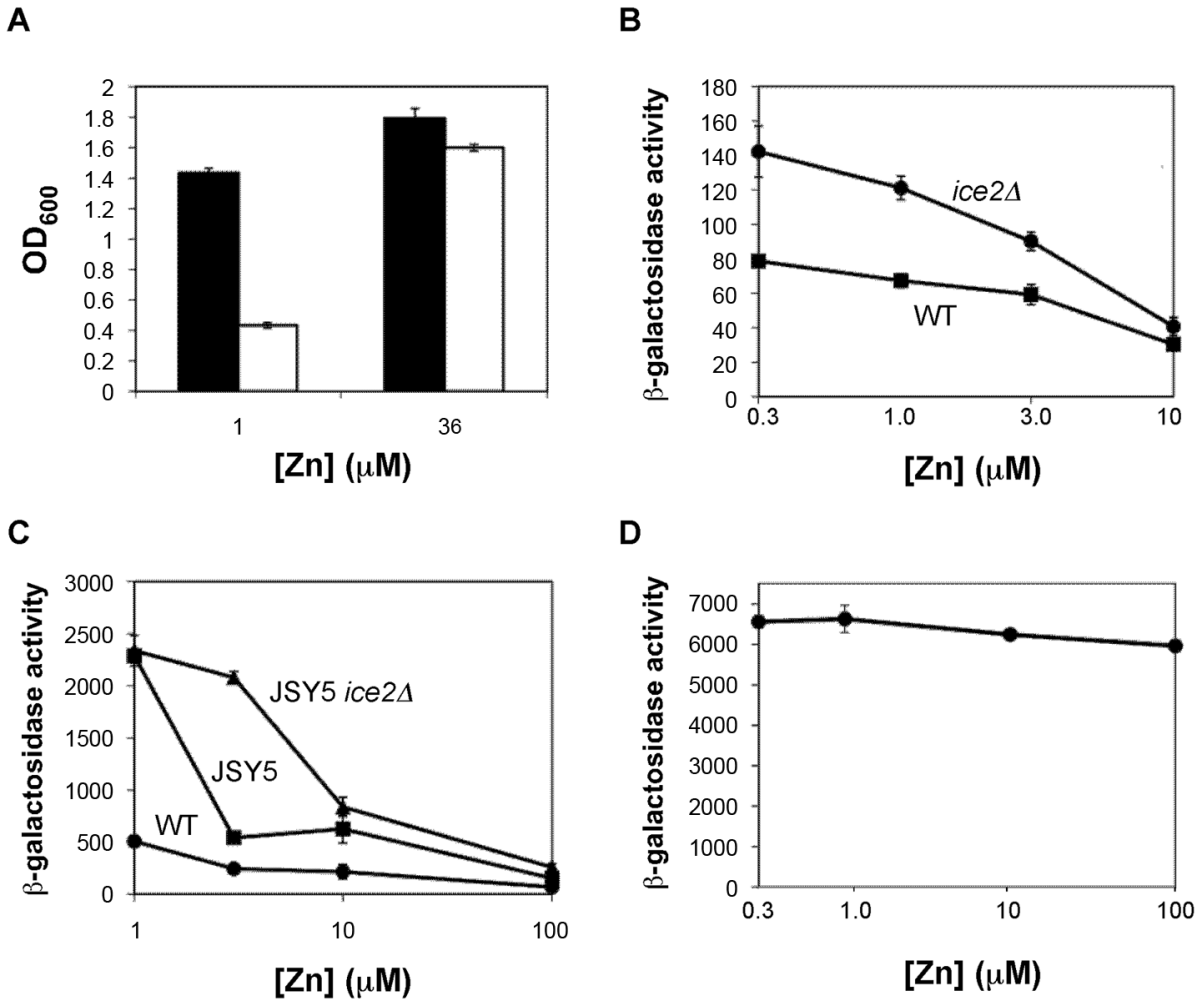
A possible role for *ICE2* in ER zinc homeostasis. A) Confirmation of the *ice2Δ* low zinc growth defect. Wild type (BY4743, *filled columns*) and *ice2Δ* (BY4743 *ice2Δ*, *open columns*) cells were inoculated into LZM supplemented with either 1 or 3 µM ZnCl_2_ and grown overnight prior to measuring the culture optical densities at 600 nm (OD_600_). B) Loss of Ice2 causes a zinc-suppressible hyper-induction of the unfolded protein response (UPR). Wild type (BY4743) and homozygous *ice2Δ* mutant (BY4743 *ice2Δ*) cells were transformed with the UPRE-lacZ reporter pMCZ-Y and inoculated into low zinc medium (LZM) supplemented with 0.3, 1, 3 or 10 µM ZnCl_2_. These cells were then grown overnight prior to measuring β–galactosidase activity. C) Loss of Ice2 exacerbates the zinc-suppressible hyper-induction of the UPR in *msc2Δ zrg17Δ zrc1Δ cot1Δ* quadruple mutants. JSY5 (*msc2Δ zrg17Δ zrc1Δ cot1Δ*) and JSY5 *ice2Δ* (*msc2Δ zrg17Δ zrc1Δ cot1Δ ice2Δ*) cells were transformed with the UPRE-lacZ reporter pMCZ-Y and inoculated into low zinc medium (LZM) supplemented with 1, 3, 10 or 100 µM ZnCl_2_. These cells were then grown overnight prior to measuring β–galactosidase activity. The wild-type strain used was the isogenic CM100 strain. D) Zinc treatment does not inhibit the UPR induction in response to tunicamycin. Wild-type BY4743 cells bearing the UPRE-lacZ reporter were grown to exponential phase in LZM supplemented with the indicated concentration of zinc, then treated for 2 hours with 2 µg/ml tunicamycin prior to β–galactosidase activity assay. Data presented are the averages of triplicate cultures for each condition and the error bars indicate ±1 S.D.

As another illustration of the power of functional profiling to identify novel genes important for low zinc growth, we focused our attention on the *ICE2* gene. *ICE2* was previously identified as being needed for proper ER morphology [33]. Ice2 is an integral membrane protein with eight potential transmembrane domains suggesting that it is a transporter of some type. Our previous results indicated that the Msc2/Zrg17 zinc transporter complex plays the major role in maintaining ER zinc while the vacuolar Zrc1 and Cot1 zinc transporters also contribute [23], [34]. Our results had also indicated that additional ER zinc transporters were present and we therefore hypothesized that the substrate for Ice2-mediated transport was zinc. Consistent with that hypothesis, *ice2Δ* mutants grew poorly in low zinc (Table 3). We confirmed this growth defect by inoculating wild type and *ice2Δ* mutant cells into batch cultures and measuring culture density after overnight growth (Figure 5A). The *ice2Δ* mutants grew poorly relative to wild-type cells in LZM+1 µM Zn but adding as little as 3 µM Zn to the medium was sufficient to restore near wild-type growth. Higher concentrations were similarly effective (data not shown). This result indicated that Ice2 is required for optimal growth under severe zinc-limiting conditions but not under more moderate conditions. Zinc-deficient wild-type cells have induced expression of the ER-stress induced unfolded protein response (UPR) and this induction is exacerbated in mutants disrupted for *MSC2* or *ZRG17* [23], [34]. Zinc supplements suppress UPR induction of both wild type and mutant cells indicating that luminal ER zinc is required for function of this compartment. Consistent with *ice2Δ* disrupting ER zinc homeostasis, we found that a UPRE-lacZ reporter was also hyper-induced in a zinc-limited *ice2Δ* mutant relative to wild-type cells and this hyper-induction was suppressed by supplementing zinc at 10 µM or above (Figure 5B, data not shown). We also deleted the *ICE2* gene in a strain in which all four genes known to contribute to ER zinc were mutated (i.e. *msc2Δ zrg17Δ zrc1Δ cot1Δ*). Loss of Ice2 function in this quadruple mutant background caused still greater induction of the UPR when zinc was supplied at a concentration of 3 µM but the UPR was still suppressible with higher concentrations of zinc (Figure 5C).

In control experiments, we confirmed that zinc supplements do not inhibit UPR induction in response to other ER stressors such as tunicamycin (Figure 5D). Therefore, these results suggest that Ice2 either directly or indirectly influences zinc homeostasis in the ER. To assess whether Ice2 could itself be a zinc transporter contributing to ER zinc levels, we tested if overexpression of Ice2 could suppress phenotypes caused by inactivation of ER zinc transport. Contrary to this hypothesis, Ice2 overexpression from the *GAL1* promoter failed to suppress the poor growth of *msc2Δ* mutants on respired carbon sources at elevated temperatures and also did not suppress UPR hyper-induction in an *msc2Δ* single mutant nor an *msc2Δ zrg17Δ zrc1Δ cot1Δ* quadruple mutant (data not shown). Thus it does not appear that Ice2 transports zinc directly but may transport another substrate that is required for optimal ER zinc trafficking.

### Analysis of mutants with apparent “resistance” to growth in low zinc

Over 500 mutant strains were identified by functional profiling as having an apparent fitness advantage in low zinc (Figure 1B). Many of these genes were found to encode subunits of the cytosolic and mitochondrial ribosomes (Table S6, Figure S1A, S1B). We tested these results independently by flow cytometry and found that the apparent zinc resistance of many of these strains is likely due to growth defects caused by these mutations under all conditions (Table 5). For example, *rsm27Δ*, *rpl2bΔ*, *rps7aΔ*, and *rpl6bΔ* mutants grown in zinc-replete conditions grew very poorly relative to wild-type cells. These mutants also grew less well than wild type in zinc-limiting conditions but were better able to compete when the wild-type strain is growth limited. Thus, a likely explanation for the functional profiling results is that in low zinc, growth of the wild-type strain is slowed by zinc deficiency to a rate closer to that of the mutant. In zinc-replete media, the wild-type strain grows at a much faster rate while the mutant growth is still impeded by the mutation and competes less well with wild type. Direct analysis of *rpl2bΔ* growth supported this hypothesis (Figure S1C). This effect could also explain the apparent low zinc resistance of *fet3Δ*, *ftr1Δ*, and *fre1Δ* mutants (Table S2); these genes are required for high affinity iron uptake and their respective mutants grow poorly in EDTA-containing LZM media regardless of zinc supplements.

**Table 5:** Flow cytometry analysis of low zinc resistant mutants.

Strain	Fitness score (log_2_)	% of mutant in initial inoculum	% of mutant after 15 gen. in 1 µM ZnCl_2_	% of mutant after 15 gen. in 100 µM ZnCl_2_	−Zn/+Zn ratio	p-value^a^
*rsm27Δ*	5.2	46.10	28.65±0.65	1.60±0.06	18.0	0.0002
*rpl2bΔ*	5.25	49.10	39.10±0.61	8.16±0.59	4.8	0.0002
*rps7aΔ*	3.55	49.90	24.50±0.92	8.28±0.67	3.0	0.002
*rpl6bΔ*	0.9	54.70	38.23±0.29	25.43±0.25	1.5	0.0006
*ubr2Δ*	1.0	50.30	70.03±0.87	47.50±0.10	1.5	0.0005
*hrd3Δ*	0.7	48.03	58.27±0.31	50.57±0.57	1.2	0.004
*yjl135wΔ*	0.95	47.40	57.43±1.04	48.53±0.32	1.2	0.007
*mrs3Δ*	0.8	49.20	65.27±0.90	54.37±0.15	1.2	0.003
*ygl034cΔ*	0.7	47.60	58.47±0.91	52.57±0.40	1.1	0.009
*yil077cΔ*	1.3	49.60	80.17±0.38	57.27±0.55	1.4	0.0005
*ydl094cΔ*	0.7	47.90	67.17±0.74	53.00±0.10	1.3	0.001

a Significance was defined as having a p-value less than 0.05 comparing the mutant prevalence after 15 generations growth in low versus high zinc; NS = not significant.

Of the 513 total strains showing apparent low zinc resistance (Table S2), 359 are documented to have a fitness disadvantage relative to wild-type cells in zinc-replete media (Saccharomyces Genome Database, http://www.yeastgenome.org/). Therefore, these mutants are likely to show apparent low zinc resistance by the same mechanism as was found for the ribosomal subunit mutants. We assayed growth of the remaining mutants with no known fitness defect and found that seven of these did indeed confer a growth advantage relative to wild-type cells in low zinc (Table 5). These include the *UBR2* and *HRD3* genes that encode protein-ubiquitin ligases involved in the degradation of aberrant cytosolic and ER proteins respectively [35], [36]. *MRS3* encodes a mitochondrial iron transporter [37]. Why mutation of these genes improves low zinc growth remains to be determined.

## Discussion

The goal of this study was to identify cellular processes in yeast that contribute to growth under zinc-limiting conditions. We found 433 different genes that are required for optimal growth in low zinc consistent with the many diverse processes in which zinc is involved. Zinc limitation might perturb cellular function by reducing the amount of cofactor available to enzymes, transcription factors, and other proteins that require the metal. Functional synergy between zinc limitation and mutations in genes important for these processes to occur optimally could exacerbate growth limitation.

### Functional profiling versus transcriptome analyses

Previous transcriptome analysis identified many genes induced in low zinc including ∼80 that are likely direct targets of the Zap1 transcription factor. We predicted that Zap1-regulated genes would be important for zinc-limited growth and several such mutants (*tsa1Δ*, *icy2Δ*, *adh4Δ*, etc) were found to have growth defects in low zinc. Surprisingly, ∼90% of Zap1 target genes (72/80) were not found to have growth defects in low zinc. These mutants may have a defect too subtle to be detected even after fifteen generations but could have a fitness disadvantage relevant to natural selection. Alternatively, these genes may be important under conditions where low zinc is combined with some other stress such as high temperature [38]. Another explanation is provided by our studies of *ZRC1*. Zrc1 is a vacuolar zinc transporter that moves zinc into the vacuole for storage and detoxification. Surprisingly, *ZRC1* is a Zap1 target gene and its expression is induced in low zinc [13]. We showed previously that this induction is not required for growth in low zinc but is needed for zinc-limited cells to survive re-supply with zinc, a condition we refer to as “zinc shock” [39]. The additional Zrc1 activity is needed to rapidly sequester the large amount of zinc that is taken up by the cell. It is possible that other Zap1 target genes are also needed to resist the toxicity of zinc shock.

With culture conditions similar to those used here, we previously identified over 400 genes that showed increased expression in zinc-limited cells [14]. Our hypothesis was that many of the genes with altered expression would be important for low zinc growth and detected in this functional profiling analysis as low zinc sensitive. Surprisingly, however, only 15 (∼4%) of the 433 genes we identified here as required for optimal low zinc growth were found previously to be up-regulated under those conditions (Table S7). This result is similar to what was previously observed for high pH, NaCl, sorbitol, or galactose treatment where only 0.3–7% of genes with increased expression under treatment conditions showed a detectable growth defect [25]. A similar lack of correlation between genes induced by DNA damaging agents and those genes needed to protect the cell against those agents has also been observed [40]. These remarkable findings indicate that, for many conditions, transcriptomic studies are a poor predictor of what genes are actually needed under adverse growth conditions. Nonetheless, the combination of transcriptomics and functional profiling studies allows us to focus on those changes in gene expression that are most critical to low zinc growth.

### The role of Zap1 target genes in low zinc

Almost half of the 15 genes with both altered expression and a detectable low zinc growth defect are Zap1 target genes. Among these are *ZAP1* itself and *ZRT1*, which encodes a high affinity zinc uptake transporter. These genes were not identified in this analysis because their mutants grow poorly in both LZM +1 and 100 µM ZnCl_2_ [19], [41]. Six other Zap1 targets were identified here and they are *ZRG17*, *TSA1*, *UTH1*, *ADH4*, *HNT1*, and *ICY2*. *ZRG17* encodes a critical subunit of the Msc2/Zrg17 transporter complex that passes zinc into the ER [34]. We showed previously that ER function is compromised in low zinc and this effect is exacerbated by loss of Msc2/Zrg17 function. *TSA1* is the major peroxiredoxin involved in oxidative stress resistance. Zinc-limited cells have elevated oxidative stress and Tsa1 is required to combat that stress [22]. *UTH1* has also been implicated in oxidative stress resistance [42] and may be important in low zinc for that reason. *UTH1* has also been implicated in maintaining cell wall integrity as well as in mitophagy [43], [44]. Uth1's role in mitophagy does not appear to explain the growth defect of the *uth1Δ* mutant because other mutants disrupted specifically for mitophagy (e.g. *atg32Δ*, *atg33Δ*) grew normally in low zinc. *ADH4* encodes an alcohol dehydrogenase isozyme that is up-regulated in zinc-limited cells at the same time that expression of two other isozymes, Adh1 and Adh3, are repressed by Zap1 [45]. This has been proposed to be a mechanism of zinc conservation because Adh1 and Adh3 bind two zinc atoms per monomer while Adh4 binds only one metal ion. Poor growth of the *adh4Δ* mutant may reflect the decreased overall ADH activity due to Zap1-mediated repression of *ADH1* and *ADH3*. *HNT1* encodes an adenosine phosphoramidase and may serve as a possible positive regulator of Kin28 [46]. Kin28 is a subunit of TFIIH and phosphorylates the carboxy-terminal domain of RNA polymerase II to maintain transcription elongation [47]. Thus, up-regulation of *HNT1* by Zap1 may enhance or help maintain transcription of genes in zinc-limited cells. Finally, *ICY2* is a gene of unknown function but its importance specifically to low zinc growth is clear from our results. *icy2Δ* mutant cells grew as well or even better than wild type cells in zinc-replete media, but were severely defective for growth in low zinc. This growth defect appears specific for zinc; *icy2Δ* mutants showed no growth defect in low iron or low copper media (data not shown).

### The importance of autophagy

In addition to identifying key Zap1 target genes, functional profiling highlighted a large number of other processes that are also important for low zinc growth. Of particular interest was the discovery of the need for autophagy in zinc-limited cells. Autophagy is a process whereby cells degrade cytosolic proteins and organelles to recycle their components [32]. Two of the regulatory factors that control autophagy are the Tor and PKA kinases that respond to nutrient starvation (i.e. nitrogen and carbon deficiency) to activate autophagic processes. A role for autophagy in zinc deficiency was hypothesized previously [48]. That zinc deficiency might in fact trigger autophagy in *S. cerevisiae* was suggested by the studies of Carman and colleagues [49] who found that accumulation of Atg8/Aut7, a marker for autophagy, increased in zinc-limited cells. Here we present evidence that autophagy is indeed important for zinc-limited growth.

Autophagy can be subdivided into several different pathways including macroautophagy, CVT, mitophagy, and pexophagy. Richie et al. previously showed that some process of autophagy was required for zinc-limited growth of *Aspergillus fumigatus* [50]. Specifically, *atg1* mutants, which are defective for all of these pathways of autophagy in *S. cerevisiae*, were defective for low zinc growth. By examining the effects of various mutants affecting different autophagy pathways, we found here that macroautophagy is required for low zinc growth of *S. cerevisiae* and that the cargo-specific pathways are less important.

What role does autophagy play in low zinc growth? An appealing hypothesis is that its purpose is to degrade zinc-containing proteins and organelles to release that zinc for other purposes. Alternatively, the stress of low zinc could damage proteins and organellar membranes and those damaged components may need to be turned over. ER stress induces autophagy [51] and the ER stress of zinc deficiency may signal autophagy to occur. Intriguingly, mutations in autophagy genes sensitizes *S. cerevisiae* to high zinc indicating the need for autophagy at both ends of the range of zinc status [52].

### Transcription factors required for low zinc growth

A large number of transcriptional regulators were identified as important in low zinc including Ace2, Crz1, Dal82, Gat1, Opi1, Rox1, and Ume6. These observations suggest that target genes of these factors are somehow needed under these conditions. Two other transcription factors that were found to be required for optimal growth in low zinc are Yap1 and Skn7. Both of these factors control the transcriptional response to oxidative stress [27], [53]. The poor growth of *yap1Δ* and *skn7Δ* mutants is consistent with the increased oxidative stress found in zinc-limited cells and the importance of Yap1/Skn7-mediated transcription. Also consistent with this hypothesis, we found that *hyr1Δ*, *ybp1Δ*, and *ybp2Δ* mutants are also defective for low zinc growth. These mutations disrupt accessory factors required for Yap1 activity [28], [29]. Paradoxically, however, no previous genome-wide expression studies identified Yap1 or Skn7 target genes as being induced in zinc-limited cells. One explanation for this apparent conflict between the transcriptomics and functional profiling is that in at least two of the transcriptome studies, a wild-type strain was used in which activation of Yap1 is much less efficient than in the strain background used in this study [54]. Thus, in strains where Yap1 induction in response to oxidative stress is strong, this transcription factor is indeed important for low zinc growth.

### Organellar function in low zinc

Our results implicate certain organelles as critical to zinc-limited growth. Surprisingly, we found that the peroxisome is particularly important. The peroxisome is best known for its role in fatty acid β–oxidation and this compartment is also a site of reactive oxygen metabolism through the action of the Cta1 catalase isozyme [55]. Mutation of *CTA1* did not confer sensitivity to low zinc indicating that the importance of the peroxisome in low zinc is not in combating oxidative stress (J. Steffen, unpublished result). A role of the peroxisome in fatty acid β–oxidation in zinc-limited cells would be surprising given that the cells were supplied with abundant glucose and not given exogenous fatty acids as carbon sources. It is conceivable that zinc-limited cells may have a defect in glucose utilization and catabolize lipid stores as a source of energy. This switch occurs in response to glucose deprivation [56] but a similar switch in low zinc/glucose-adequate conditions remains to be tested. In support of this hypothesis, we observed that mutations in *FAT1* encoding a peroxisomal fatty acid transporter and *POT1*, encoding 3-ketoacyl-CoA thiolase caused some growth defect in low zinc (Table S1). These genes are required for fatty acid β–oxidation. Alternatively, other peroxisomal functions such as the glyoxylate cycle or purine metabolism may be required.

Another organelle that is clearly critical for growth in low zinc is the endoplasmic reticulum. We showed previously that zinc deficiency induces ER stress in wild-type cells and that this stress is exacerbated by loss of the Msc2/Zrg17 transporter complex that passes zinc into the ER lumen. Consistent with these results, we observed here that mutants lacking Msc2 or Zrg17 show a low zinc growth defect. We also found that loss of either Ire1 or Hac1, the regulatory components of the ER stress response pathway, also impairs low zinc growth. This is consistent with the need to counter the increased ER stress. More unexpectedly, we found that genes involved in ER protein folding (e.g. *JEM1*, *SCJ1*), glycosylation (e.g. *ALG3*, *ALG5*, *OST3*, *OST5*), ER Ca homeostasis (*SPF1*), ER-to-Golgi trafficking (e.g. *BRE5*, *ERD1*, *ERP4*), and other ER processes were also sensitive to low zinc. One property shared by most if not all of the ER-related mutants we identified is that they cause ER stress or reduce the ability of the cells to respond to that stress [57]. Because zinc deficiency itself is a cause of ER stress, one likely hypothesis to explain the importance of so many ER-related genes is that the poor growth is the result of combined perturbation of ER function. This hypothesis was supported by our observation that zinc-limited cells are hypersensitive to tunicamycin. ER stress can generate increased oxidative stress [58]. Therefore, an alternative hypothesis is that disruption of ER function adds to the oxidative stress experienced normally by zinc-deficient cells. Disruption of ER function appears to be zinc-specific; no defect was observed for either *hac1Δ* or *ire1Δ* mutants in either low iron or low copper minimal media that were zinc replete (Table S5).

As one final example, we identified the *ICE2* gene as potentially important for ER zinc homeostasis. This gene was not required for growth in either low iron or low copper (Table S5). Ice2 is a predicted integral membrane protein with eight transmembrane domains and as other transporter proteins are required for zinc transport into ER lumen, we hypothesized that Ice2 might be a zinc transporter. While our results did not support this hypothesis, they did suggest that Ice2 is required for ER zinc homeostasis in some way. This conclusion was supported by results of a genome-wide screen of synthetic growth defects that linked Ice2 function with that of Zrg17 and the ER zinc transporter complex [59]. Thus, this functional profiling analysis has identified key metabolic processes that play important roles in low zinc and has also identified possible new roles for specific genes of unknown function.

## Materials and Methods

### Strains and media

Diploid yeast deletion strains used for functional profiling and for analysis of individual strains were of the BY4743 background (*MATa/MATα his3*Δ*1/his3*Δ*1 leu2*Δ*0/leu2*Δ*0 lys2*Δ*0/LYS2 MET15/met15*Δ*0 ura3*Δ*0/ura3*Δ*0*) (Invitrogen Corporation, Carlsbad, CA). JSY5 has the genotype of *MATα his3Δ leu2Δ trp1Δ ura3Δ msc2Δ::kanMX4 zrg17Δ::natMX4 zrc1Δ::HIS3 cot1Δ::URA3*. Cells were grown in a rich medium (YPD, 1% yeast extract, 2% peptone, 2% glucose) or low zinc medium (LZM) prepared as previously described [60]. LZM is zinc limiting because it contains 1 mM EDTA and 20 mM citrate to buffer metal availability. Therefore, only a small fraction of the total zinc in LZM is available for uptake by cells. Zinc was supplied as ZnCl_2_. An iron-limiting medium was generated by adding the iron chelator bathophenanthroline disulfonate (BPS, 100 µM) and copper limitation was imposed by adding the copper chelator bathocuproine disulfonate (BCS, 200 µM) to YPD medium. Also, LZM was made iron-limiting by leaving out the iron supplement and adding 100 µM ZnCl_2_ to make it zinc replete. Iron was then supplemented to either 1 (low iron) or 100 (replete iron) µM FeCl_3_. LZM was modified to control copper availability by leaving out the copper supplement and replacing the EDTA with 200 µM BCS. Copper was then supplemented to either 0.1 (low copper) or 10 (replete copper) µM CuCl_2_. Preliminary growth experiments confirmed that these conditions were limiting or replete for the respective metal.

### Growth curve assays

To assess the effect of limiting zinc on growth, yeast strains were pre-grown to mid-log phase in LZM+100 µM ZnCl_2_, diluted to an optical density at 595 nm (OD_595_) of 0.0165 in either LZM+1 µM ZnCl_2_ or LZM+100 µM ZnCl_2_, and dispensed in triplicate into different wells of a 48-well plate (non-treated polystyrene, Grenier Bio-One, Monroe, NC). Plates were incubated in a GENios microplate reader (Tecan, Durham, NC) set to 30°C with intermittent shaking. OD_595_ measurements were taken at 15-minute intervals for a period of 24 hours. Raw absorbance data were averaged for all replicates, background corrected, and plotted as a function of time. The area under the curve (AUC) as a measure of growth was calculated with Excel 2008 (Microsoft Corporation, Redmond, WA).

#### Cell culturing for functional profiling analysis

For the analysis of fitness over five generations, cells from frozen aliquots of the deletion collection pools were inoculated into 100 ml culture volumes in 250 ml metal-free polycarbonate Erlenmeyer flasks to a starting OD_600_ of 0.04 (6×10^5^ cells per ml). The cultures were then incubated with aeration at 30°C and grown to a final OD_600_ of 1.2 (1.8×10^7^ cells per ml). For the fifteen-generation experiments, the cells were inoculated at a starting OD_600_ of 0.005 (7.5×10^4^ cells per ml) and grown to an OD_600_ of 1.2 (eight generations), reinoculated into fresh media at a starting OD_600_ of 0.01 (1.5×10^5^ cells per ml) and grown to a final OD_600_ of 1.2 (seven generations). The initial inocula are equal to 0.75–6×10^7^ total cells. Thus, the cultures were inoculated with ∼1,600–13,000 cells of each mutant genotype. Four independent replicate cultures were used for each condition of zinc and generation number. After the desired generation number was reached, the cells were chilled, centrifuged at 1000×g for 6 min at 4°C, and the pellets resuspended in 2 ml dH_2_0. The resuspensions were divided equally among three microfuge tubes, centrifuged at 1000×g for 5 min, the supernatant was removed and the pellets frozen at −80°C.

#### Functional profiling analysis

Extraction of genomic DNA from cultured cells, PCR amplification of barcodes, and Affymetrix TAG4 array hybridization were performed as previously described [61]. An added genomic DNA purification step was conducted following DNA extraction using the Zymo Genomic DNA Clean and Concentrator kit (Zymo Research Corporation, Irvine, CA). The data discussed in this publication have been deposited in NCBI's Gene Expression Omnibus and are accessible through GEO Series accession number GSE37254 (http://www.ncbi.nlm.nih.gov/geo/query/acc.cgi?acc=GSE37254).

### Differential strain sensitivity analysis (DSSA)

Raw microarray data were processed and significant strains identified as previously described [61]. Briefly, a fitness score (ave(log_2_{Y|X = LZM1})-ave(log_2_{Y|X = LZM100}), the difference in the mean of the log_2_ hybridization signal between LZM+1 µM ZnCl_2_ and LZM+100 µM ZnCl_2_) was calculated for each deletion strain, to identify those differentially sensitive. This fitness score is an indicator of the effect of low zinc on the growth of the deletion strain. A negative fitness score indicates the deletion strain is sensitive to zinc limitation.

Significant genes (strains) were statistically inferred using an exact binomial test, assuming that the outcomes for each gene in all effective treatment-control pairs were independent binary variables with the same probability of success (p = 0.5) for all trials (Bernoulli trials) [61]. For a particular gene n, outcomes were considered as “successful” if they were significant in the outlier analysis with q-values≤0.05 in each of all effective pairs with log2 ratios of the same sign, simultaneously. The corresponding raw p-values based on the exact binomial test were then corrected for multiplicity of comparisons using q-value approach and only the genes with q-value≤0.05 were considered for further analysis. This approach does not apply a scale estimator and, as a result, it does not require between-chip pair normalization for the statistical inference.

### Functional enrichment analysis of DSSA results

Data sets from DSSA were verified for enrichment of any particular biological attribute by identifying significantly overrepresented Gene Ontology (GO) categories [62] and MIPS (Munich Information Center for Protein Sequences) categories [63] by a hypergeometric distribution using the Functional Specification resource, FunSpec (http://funspec.med.utoronto.ca/) [64], with a p-value cutoff of 0.01 and Bonferroni correction.

### Cytoscape network mapping analysis

Fitness data for all significantly sensitive strains identified from this study were mapped onto the *S. cerevisiae* BioGRID interaction dataset [65] using the software package Cytoscape [66]. The jActiveModules Cytoscape plug-in [67] was then used to identify functional modules (sub-networks) within the BioGRID network based on fitness data that are enriched for nodes (genes) that have sensitive fitness data associated with them, and so may identify cellular processes or protein complexes affected by zinc limitation. The sub-network with the highest significance score, comprising 283 genes (including both sensitive genes and the non-sensitive and essential genes that link them through known genetic and physical interactions) was then assessed for significant overrepresentation of Gene Ontology (GO) categories using the Cytoscape plug-in BiNGO [68]. A similar approach was used for analyzing the mutants with apparent low zinc resistance.

### Analysis of relative growth by flow cytometry

The BY4743 wild-type strain was transformed with an integrating vector that expresses GFP from the strong *TEF2* promoter [26]. The site of insertion is the *HIS3* gene that is already mutant in the parental strain. The GFP-tagged wild-type strain and untagged isogenic mutant strains were grown overnight to exponential phase (OD_600_ = 0.5) in YPD, mixed in approximately equal numbers, and then inoculated into LZM+1 or 100 µM ZnCl_2_. Cells were first inoculated into 5 ml cultures with a starting OD_600_ of 0.00375 and grown to an OD_600_ of 1.0. These cells were then inoculated into fresh cultures of the same media to an OD_600_ of 0.0075 and grown to a final OD_600_ of 1.0 for a total of fifteen generations. Approximately 20,000 total cells per culture were analyzed by flow cytometry using a FACSCalibur flow cytometer (BD Biosciences) and an air-cooled 488 nm argon ion laser. A 530/30 filter was used for measuring GFP fluorescence and a 585/42 filter was used to detect autofluorescence exhibited by both tagged and untagged cells. GFP-expressing wild type cells were readily distinguished from untagged mutant cells. In control experiments, wild-type cells that had lost GFP expression were found to be rare (i.e. <1% of the total cells) and therefore contribute little variability to mutant cell counts. Means of three independent cultures are reported with standard deviations and statistically significant differences between the low zinc and zinc-replete cultures were determined using Student's t-test.

## Supporting Information

Figure S1 Analysis of mutants with apparent resistance to low zinc growth. Mutations that disrupt the cytosolic (A) and mitochondrial (B) ribosomal subunits show apparent resistance to low zinc. C) An *rpl2bΔ* mutant grows as poorly in LZM+1 µM ZnCl_2_ (LZM1) as in LZM+100 µM ZnCl_2_ (LZM100). Because the wild-type strain grows less well in low zinc relative to high zinc, the proportion of the mutant in the low zinc culture is greater than in the high zinc culture so the mutant appears to be resistant to low zinc.
(PDF)

Table S1 Yeast gene deletion mutants with increased sensitivity to low zinc. Deletion mutants identified in the functional profiling analysis as sensitive to low zinc are listed.
(PDF)

Table S2 Yeast gene deletion mutants with increased tolerance to low zinc. Deletion mutants identified in the functional profiling analysis as resistant to low zinc are listed.
(PDF)

Table S3 Yeast gene deletion mutants unaffected in low zinc. Deletion mutants tested in the functional profiling analysis but not identified as sensitive or resistant to low zinc are listed.
(PDF)

Table S4 Autophagy is not greatly required for low iron or low copper growth. Various autophagy mutants showing growth defects in low zinc were cultured in metal replete (YPD) and low iron (YPD+100 µM BPS) or low copper (YPD+200 µM BCS) and assayed for growth as described for Table 1.
(PDF)

Table S5 Effects of mutations in autophagy and ER stress response on growth of iron- or copper-limited cells that are zinc replete. Various autophagy mutants and mutants disrupting ER function were cultured in metal replete, low iron, and low copper conditions and assayed for growth as described for Table 1. LZM was made iron-limiting by leaving out the iron supplement and adding 100 µM ZnCl_2_ to make it zinc replete. Iron was then supplemented to either 1 (low iron) or 100 (replete iron) µM FeCl_3_. LZM was modified to control copper availability by leaving out the copper supplement and replacing the EDTA with 200 µM BCS. Copper was then supplemented to either 0.1 (low copper) or 10 (replete copper) µM CuCl_2_.
(PDF)

Table S6 Enrichment among low zinc-resistant mutants for molecular function, biological processes, and other categories.
(PDF)

Table S7 Zinc-regulated genes whose mutants were sensitive to low zinc. Deletion mutants identified in the functional profiling analysis as sensitive to low zinc and also known from other studies to be induced in low zinc are listed.
(PDF)
