# Supplementary figures and images for: Genome-Wide Functional Profiling Identifies Genes and Processes Important for Zinc-Limited Growth of Saccharomyces cerevisiae
Source: PLoS Genet. 2012 Jun 7;8(6):e1002699. doi: 10.1371/journal.pgen.1002699 (PMC3369956; doi:10.1371/journal.pgen.1002699)

Supplemental Figure 1

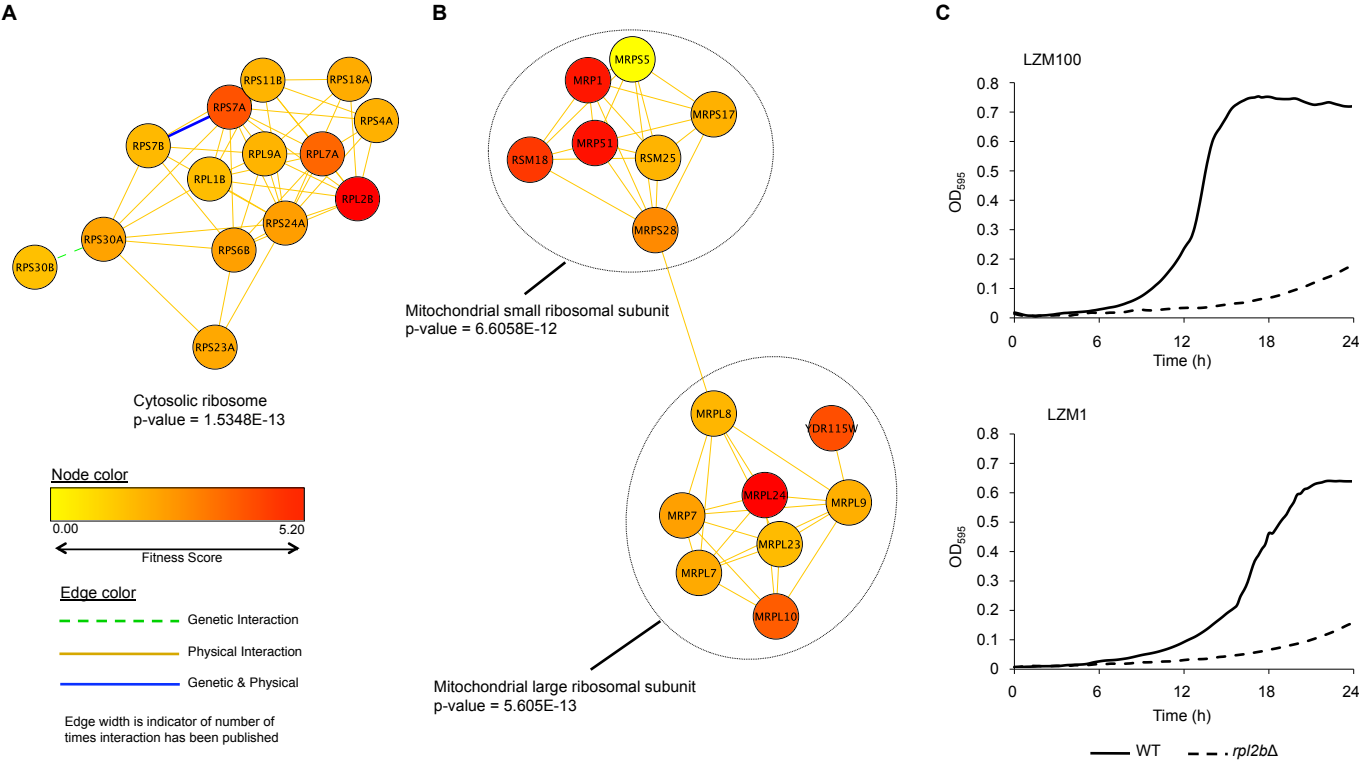

Supplement: Figure S1 — Analysis of mutants with apparent resistance to low zinc growth. Mutations that disrupt the cytosolic (A) and mitochondrial (B) ribosomal subunits show apparent resistance to low zinc. C) An rpl2bΔ mutant grows as poorly in LZM+1 µM ZnCl2 (LZM1) as in LZM+100 µM ZnCl2 (LZM100). Because the wild-type strain grows less well in low zinc relative to high zinc, the proportion of the mutant in the low zinc culture is greater than in the high zinc culture so the mutant appears to be resistant to low zinc. (PDF) [file pgen.1002699.s001.pdf]
