# Supplementary material for: Genome-Wide Functional Profiling Identifies Genes and Processes Important for Zinc-Limited Growth of Saccharomyces cerevisiae
Source: PLoS Genet. 2012 Jun 7;8(6):e1002699. doi: 10.1371/journal.pgen.1002699 (PMC3369956; doi:10.1371/journal.pgen.1002699)
Supplement: Table S1 — Yeast gene deletion mutants with increased sensitivity to low zinc. Deletion mutants identified in the functional profiling analysis as sensitive to low zinc are listed. (PDF) [file pgen.1002699.s002.pdf]

Supplemental Table 1. Yeast gene deletion mutants with increased sensitivity to low zinc.

ORF	Gene	5 generations	15 generations
YOR216C	*RUD3*	-1.2	-5.3
YNR051C	*BRE5*		-5.1
YDR149C		-0.9	-4.6
YHR030C	*SLT2*		-4.6
YCR068W	*ATG15*	-1.2	-4.45
YPL195W	*APL5*		-3.95
YDR225W	*HTA1*	-2.1	-3.9
YDR455C		-0.7	-3.9
YKL048C	*ELM1*	-0.7	-3.9
YNL329C	*PEX6*		-3.9
YMR073C	*IRC21*		-3.9
YHR129C	*ARP1*	-1.3	-3.85
YDR265W	*PEX10*		-3.8
YOL012C	*HTZ1*		-3.75
YOR360C	*PDE2*	-1.2	-3.75
YGR184C	*UBR1*	-1.75	-3.7
YLR425W	*TUS1*		-3.7
YMR294W	*JNM1*	-1	-3.65
YGL153W	*PEX14*		-3.6
YKR035W-A	*DID2*		-3.6
YBR288C	*APM3*		-3.55
YFL032W		-1.15	-3.5
YBR131W	*CCZ1*		-3.5
YGR077C	*PEX8*		-3.5
YJL024C	*APS3*		-3.5
YJL211C			-3.5
YER167W	*BCK2*	-1.1	-3.45
YFR040W	*SAP155*	-1.7	-3.45
YHR079C	*IRE1*	-1.45	-3.4
YJL080C	*SCP160*	-1.7	-3.3
YBR227C	*MCX1*		-3.3
YDL203C	*ACK1*		-3.3
YMR284W	*YKU70*		-3.3
YMR275C	*BUL1*		-3.3
YPL120W	*VPS30*		-3.25
YDR414C	*ERD1*		-3.25
YOL044W	*PEX15*		-3.2

YKL197C	*PEX1*		-3.15
YJR117W	*STE24*		-3.1
YIL090W	*ICE2*	-1.1	-3.1
YMR263W	*SAP30*	-0.7	-3.1
YDR456W	*NHX1*		-3.1
YGR133W	*PEX4*		-3
YDL118W		-1.2	-3
YNL324W			-3
YML055W	*SPC2*	-1.5	-2.9
YCR036W	*RBK1*	-0.95	-2.9
YAL055W	*PEX22*		-2.9
YDL065C	*PEX19*		-2.9
YNL067W	*RPL9B*		-2.9
YDR329C	*PEX3*		-2.85
YGL256W	*ADH4*		-2.85
YMR304W	*UBP15*	-1.45	-2.8
YNL325C	*FIG4*	-0.7	-2.8
YBL083C			-2.8
YLR055C	*SPT8*	-1.1	-2.75
YIL148W	*RPL40A*	-0.8	-2.75
YAL040C	*CLN3*	-1.4	-2.7
YBR103W	*SIF2*	-1.55	-2.7
YLR191W	*PEX13*		-2.7
YPR201W	*ARR3*		-2.7
YKL037W			-2.7
YDR443C	*SSN2*		-2.7
YGR261C	*APL6*		-2.7
YKL190W	*CNB1*		-2.7
YNL025C	*SSN8*		-2.7
YMR106C	*YKU80*		-2.65
YNR029C			-2.6
YBR291C	*CTP1*		-2.6
YGL152C			-2.6
YER059W	*PCL6*	-0.85	-2.55
YKL114C	*APN1*	-1.45	-2.5
YBR298C	*MAL31*	-0.8	-2.5
YLR360W	*VPS38*		-2.5
YMR299C	*DYN3*		-2.5
YLR373C	*VID22*	-1	-2.45
YPL152W	*RRD2*	-0.95	-2.45
YPL174C	*NIP100*	-0.8	-2.45
YCR081W	*SRB8*		-2.45
YDR335W	*MSN5*		-2.45
YCR033W	*SNT1*	-0.85	-2.4
YDR150W	*NUM1*	-0.7	-2.4
YNL264C	*PDR17*		-2.4

YCR026C	*NPP1*	-0.7	-2.4
YEL031W	*SPF1*	-1.25	-2.35
YBR249C	*ARO4*		-2.35
YCL005W	*LDB16*		-2.3
YGL124C	*MON1*		-2.3
YKL032C	*IXR1*		-2.3
YGR284C	*ERV29*		-2.3
YOL115W	*PAP2*		-2.3
YML115C	*VAN1*		-2.25
YNL016W	*PUB1*		-2.2
YDR486C	*VPS60*		-2.2
YCR009C	*RVS161*		-2.15
YEL029C	*BUD16*		-2.15
YLL049W	*LDB18*		-2.1
YFL031W	*HAC1*		-2.1
YBL039C	*URA7*		-2.05
YJR044C	*VPS55*		-2.05
YER161C	*SPT2*		-2.05
YDL226C	*GCS1*		-2.05
YOR322C	*LDB19*		-2.05
YMR016C	*SOK2*		-2
YOR124C	*UBP2*		-2
YLL038C	*ENT4*	-0.8	-2
YDL074C	*BRE1*		-2
YIL036W	*CST6*		-2
YJL127C	*SPT10*	-0.7	-1.9
YBL031W	*SHE1*		-1.9
YDR043C	*NRG1*		-1.9
YBR023C	*CHS3*		-1.9
YJL036W	*SNX4*		-1.85
YLR436C	*ECM30*	-0.8	-1.8
YJL154C	*VPS35*		-1.8
YOL129W	*VPS68*		-1.8
YML071C	*COG8*		-1.8
YJR054W			-1.8
YCL029C	*BIK1*		-1.8
YNR007C	*ATG3*		-1.8
YGL160W			-1.8
YPL042C	*SSN3*		-1.8
YGL194C	*HOS2*	-0.9	-1.75
YCL001W-A		-0.8	-1.75
YDR005C	*MAF1*		-1.75
YDL066W	*IDP1*		-1.75
YNL291C	*MID1*		-1.75
YJL053W	*PEP8*		-1.75
YOL004W	*SIN3*	-1	-1.7

YKR029C	*SET3*	-1	-1.7
YML117W	*NAB6*	-0.7	-1.7
YOR085W	*OST3*	-0.75	-1.7
YBL078C	*ATG8*		-1.7
YIL135C	*VHS2*		-1.7
YDR388W	*RVS167*		-1.7
YCR034W	*FEN1*		-1.65
YKR042W	*UTH1*		-1.65
YOR067C	*ALG8*		-1.65
YBR015C	*MNN2*		-1.65
YLR418C	*CDC73*	-0.95	-1.6
YCR045C		-0.8	-1.6
YIL044C	*AGE2*		-1.6
YOL116W	*MSN1*		-1.6
YPR065W	*ROX1*		-1.6
YOR002W	*ALG6*		-1.6
YKL077W			-1.6
YCR087W		-1.1	-1.6
YHL009C	*YAP3*		-1.6
YNL323W	*LEM3*		-1.6
YDR488C	*PAC11*		-1.6
YHR012W	*VPS29*		-1.6
YNL097C	*PHO23*	-0.8	-1.55
YGR276C	*RNH70*	-0.5	-1.55
YLR434C			-1.55
YKR052C	*MRS4*		-1.55
YHR206W	*SKN7*	-0.6	-1.55
YCR050C		-0.9	-1.5
YLR335W	*NUP2*		-1.5
YDR363W	*ESC2*		-1.5
YJR033C	*RAV1*		-1.5
YOL064C	*MET22*		-1.5
YJL115W	*ASF1*		-1.5
YEL007W			-1.5
YKL041W	*VPS24*		-1.5
YOL018C	*TLG2*		-1.5
YDR425W	*SNX41*		-1.5
YDR276C	*PMP3*		-1.45
YJL192C	*SOP4*		-1.45
YBR229C	*ROT2*		-1.45
YNR039C	*ZRG17*		-1.45
YBL082C	*ALG3*		-1.45
YGR289C	*MAL11*		-1.45
YGR170W	*PSD2*		-1.45
YCR044C	*PER1*	-0.85	-1.4
YOR269W	*PAC1*		-1.4

YCR011C	ADP1		-1.4
YDR426C			-1.4
YML017W	PSP2		-1.4
YNL074C	MLF3		-1.4
YOR304C-A			-1.4
YMR010W			-1.4
YBL091C	MAP2		-1.4
YCR087C-A	LUG1		-1.4
YPL227C	ALG5		-1.4
YGR064W			-1.4
YLR058C	SHM2		-1.35
YOR068C	VAM10		-1.35
YHR204W	MNL1		-1.35
YBL046W	PSY4		-1.35
YBL061C	SKT5		-1.35
YLR079W	SIC1	-0.85	-1.3
YML007W	YAP1		-1.3
YML103C	NUP188		-1.3
YDR496C	PUF6		-1.3
YGR270W	YTA7		-1.3
YJL123C			-1.3
YDL125C	HNT1		-1.3
YDR505C	PSP1		-1.3
YNL027W	CRZ1		-1.3
YDL173W		-1.15	-1.25
YPR064W			-1.25
YGL228W	SHE10		-1.25
YNL219C	ALG9		-1.25
YJR075W	HOC1		-1.25
YGL027C	CWH41		-1.25
YGR063C	SPT4		-1.25
YNL170W		-1.9	-1.2
YJL152W		-0.6	-1.2
YDR169C	STB3		-1.2
YBL062W			-1.2
YDR108W	GSG1		-1.2
YKR041W			-1.2
YOR298C-A	MBF1		-1.2
YDR253C	MET32		-1.2
YIL112W	HOS4		-1.2
YDR451C	YHP1		-1.2
YDR424C	DYN2		-1.2
YPL138C	SPP1		-1.2
YNR030W	ALG12		-1.2
YDR289C	RTT103		-1.2
YDL162C			-1.2
YNR040W			-1.15
YER020W	GPA2		-1.15

YLR404W			-1.15
YDR395W	*SXM1*		-1.15
YGR004W	*PEX31*		-1.15
YHL023C	*RMD11*		-1.15
YOR123C	*LEO1*	-1.15	-1.1
YDR290W		-0.9	-1.1
YDL172C		-0.8	-1.1
YLL013C	*PUF3*		-1.1
YGR106C			-1.1
YMR214W	*SCJ1*		-1.1
YDR466W	*PKH3*		-1.1
YDR294C	*DPL1*		-1.1
YML035C-A			-1.1
YOR008C-A			-1.1
YPL066W			-1.1
YGR217W	*CCH1*		-1.1
YOR069W	*VPS5*		-1.1
YJL051W	*IRC8*		-1.1
YPL253C	*VIK1*		-1.1
YIL162W	*SUC2*		-1.1
YMR276W	*DSK2*		-1.1
YGL226C-A	*OST5*		-1.1
YBR260C	*RGD1*		-1.1
YML010W-A			-1.1
YNL283C	*WSC2*		-1.1
YGL003C	*CDH1*		-1.1
YDR112W	*IRC2*		-1.1
YKR051W			-1.1
YNL183C	*NPR1*		-1.1
YDR348C			-1.1
YBR041W	*FAT1*		-1.05
YBR231C	*SWC5*		-1.05
YEL062W	*NPR2*		-1.05
YBR009C	*HHF1*		-1.05
YDR295C	*HDA2*		-1.05
YDR071C	*PAA1*		-1.05
YBR171W	*SEC66*	-1.2	-1
YJL128C	*PBS2*		-1
YBR036C	*CSG2*		-1
YPR179C	*HDA3*		-1
YOR364W			-1
YHR142W	*CHS7*		-1
YHR181W	*SVP26*		-1
YNL041C	*COG6*		-1
YMR152W	*YIM1*		-1
YDL133W			-1

YLR131C	*ACE2*		-1
YGR227W	*DIE2*		-1
YOR344C	*TYE7*		-1
YBR216C	*YBP1*		-1
YIR037W	*HYR1*		-0.95
YHR078W			-0.95
YEL061C	*CIN8*		-0.95
YMR190C	*SGS1*		-0.95
YBR027C			-0.95
YML006C	*GIS4*	-0.8	-0.9
YNL314W	*DAL82*		-0.9
YNL322C	*KRE1*		-0.9
YBR263W	*SHM1*		-0.9
YFL044C	*OTU1*		-0.9
YKL149C	*DBR1*		-0.9
YML010C-B			-0.9
YOR371C	*GPB1*		-0.9
YDR525W	*API2*		-0.9
YIL038C	*NOT3*		-0.9
YML032C	*RAD52*		-0.9
YML008C	*ERG6*		-0.9
YGR007W	*MUQ1*		-0.9
YNL224C	*SQS1*		-0.9
YGR197C	*SNG1*		-0.9
YKR040C			-0.9
YPR087W	*VPS69*		-0.9
YGL060W	*YBP2*		-0.9
YLR412W			-0.9
YOR132W	*VPS17*		-0.9
YLR269C			-0.9
YOR112W	*CEX1*		-0.9
YGR250C			-0.9
YHL002W	*HSE1*		-0.9
YCL038C	*ATG22*		-0.9
YJL178C	*ATG27*		-0.85
YCR076C			-0.85
YJR142W			-0.85
YNL201C	*PSY2*		-0.85
YER072W	*VTC1*		-0.85
YHR004C	*NEM1*		-0.8
YAL009W	*SPO7*		-0.8
YOR352W			-0.8
YHR031C	*RRM3*		-0.8
YDR254W	*CHL4*		-0.8
YPL245W			-0.8
YFL043C			-0.8

YMR167W	*MLH1*		-0.8
YJR083C	*ACF4*		-0.8
YOL110W	*SHR5*		-0.8
YJL193W			-0.8
YLL007C			-0.8
YIL137C	*TMA108*		-0.8
YDR006C	*SOK1*		-0.8
YBR277C			-0.8
YER143W	*DDI1*		-0.8
YIL027C	*KRE27*		-0.8
YOL035C			-0.8
YMR199W	*CLN1*		-0.8
YML012W	*ERV25*		-0.8
YOR140W	*SFL1*		-0.8
YJL146W	*IDS2*		-0.8
YHR195W	*NVJ1*		-0.8
YBR255W			-0.8
YMR070W	*MOT3*		-0.8
YHR135C	*YCK1*		-0.8
YNR006W	*VPS27*		-0.8
YMR179W	*SPT21*		-0.8
YLL024C	*SSA2*		-0.8
YKL160W	*ELF1*		-0.8
YGL049C	*TIF4632*		-0.8
YCR032W	*BPH1*		-0.8
YOL128C	*YGK3*		-0.8
YPL269W	*KAR9*		-0.8
YDR205W	*MSC2*		-0.8
YDL180W			-0.8
YKL063C			-0.8
YNL076W	*MKS1*		-0.8
YOL124C	*TRM11*		-0.8
YER005W	*YND1*		-0.8
YCR008W	*SAT4*	-0.8	-0.75
YBR278W	*DPB3*		-0.75
YOL036W			-0.75
YOL087C		-0.8	-0.7
YOR351C	*MEK1*		-0.7
YLR433C	*CNA1*		-0.7
YBL067C	*UBP13*	-0.7	-0.7
YOR223W			-0.7
YKR032W			-0.7
YLR176C	*RFX1*		-0.7
YOR016C	*ERP4*		-0.7
YOR276W	*CAF20*		-0.7
YIL125W	*KGD1*		-0.7

YDR334W	*SWR1*		-0.7
YFL025C	*BST1*		-0.7
YCR086W	*CSM1*		-0.7
YMR159C	*ATG16*		-0.7
YML095C	*RAD10*		-0.7
YIL119C	*RPI1*		-0.7
YKL047W			-0.7
YBR217W	*ATG12*		-0.7
YLR225C			-0.7
YOR233W	*KIN4*		-0.7
YMR238W	*DFG5*		-0.7
YPL022W	*RAD1*		-0.7
YPL150W			-0.7
YNR021W			-0.7
YHL029C	*OCA5*		-0.7
YPL261C			-0.7
YHR045W			-0.7
YKL034W	*TUL1*		-0.7
YEL042W	*GDA1*		-0.7
YNR067C	*DSE4*		-0.6
YHL040C	*ARN1*		-0.6
YJL073W	*JEM1*		-0.6
YIL160C	*POT1*		-0.6
YOR017W	*PET127*		-0.6
YFL021W	*GAT1*		-0.6
YBL056W	*PTC3*		-0.6
YCR060W	*TAH1*		-0.5
YDR392W	*SPT3*	-2.7	
YLL039C	*UBI4*	-2.6	
YIL083C		-2.3	
YOL081W	*IRA2*	-2.1	
YJL175W		-2	
YLR315W	*NKP2*	-1.9	
YHL025W	*SNF6*	-1.9	
YGL168W	*HUR1*	-1.9	
YDR028C	*REG1*	-1.85	
YPL250C	*ICY2*	-1.8	
YAL013W	*DEP1*	-1.6	
YML028W	*TSA1*	-1.6	
YHR013C	*ARD1*	-1.4	
YGL212W	*VAM7*	-1.3	
YJR073C	*OPI3*	-1.3	
YDL040C	*NAT1*	-1.25	
YGR056W	*RSC1*	-1.2	
YJL184W	*GON7*	-1.2	
YDR484W	*VPS52*	-1.2	

YOR106W	*VAM3*	-1.2	
YHR018C	*ARG4*	-1.2	
YJL029C	*VPS53*	-1.2	
YHL020C	*OPI1*	-1.15	
YLR322W	*VPS65*	-1.15	
YBR156C	*SLI15*	-1.1	
YLR226W	*BUR2*	-1.1	
YMR054W	*STV1*	-1.05	
YDR207C	*UME6*	-1.05	
YOR198C	*BFR1*	-1	
YLR386W	*VAC14*	-1	
YLR148W	*PEP3*	-1	
YGL054C	*ERV14*	-0.95	
YDR080W	*VPS41*	-0.9	
YMR062C	*ECM40*	-0.9	
YDR372C	*VPS74*	-0.85	
YIL052C	*RPL34B*	-0.8	
YGL244W	*RTF1*	-0.8	
YPL254W	*HFI1*	-0.8	
YDR173C	*ARG82*	-0.8	
YER111C	*SWI4*	-0.8	
YKL054C	*DEF1*	-0.8	
YCR084C	*TUP1*	-0.7	
YNL236W	*SIN4*	-0.7	
YGR057C	*LST7*	-0.7	
YNL206C	*RTT106*	-0.6	
