# Supplementary material for: Genome-Wide Functional Profiling Identifies Genes and Processes Important for Zinc-Limited Growth of Saccharomyces cerevisiae
Source: PLoS Genet. 2012 Jun 7;8(6):e1002699. doi: 10.1371/journal.pgen.1002699 (PMC3369956; doi:10.1371/journal.pgen.1002699)
Supplement: Table S2 — Yeast gene deletion mutants with increased tolerance to low zinc. Deletion mutants identified in the functional profiling analysis as resistant to low zinc are listed. (PDF) [file pgen.1002699.s003.pdf]

Supplemental Table 2. Yeast gene deletion mutants with increased tolerance to low zinc.

ORF	Gene	5 generations	15 generations
YIL018W	*RPL2B*	1	5.25
YMR193W	*MRPL24*	0.8	5.2
YGR215W	*RSM27*	0.8	5.2
YBL038W	*MRPL16*		5.1
YOR065W	*CYT1*	0.7	5
YPL118W	*MRP51*	0.85	4.9
YDR347W	*MRP1*	1	4.8
YER087W		1	4.65
YOR205C	*FMP38*	0.8	4.6
YLR069C	*MEF1*	0.95	4.5
YLL033W	*IRC19*	0.95	4.5
YGR285C	*ZUO1*	1.1	4.25
YDR140W	*MTQ2*	1	4.2
YDR298C	*ATP5*	1	4.2
YOR035C	*SHE4*	1.5	4.15
YNL229C	*URE2*		4.15
YMR205C	*PFK2*	0.7	4.1
YER050C	*RSM18*	1.1	4.1
YFL016C	*MDJ1*		4.1
YMR097C	*MTG1*	0.7	4.05
YDL044C	*MTF2*	0.7	4
YEL050C	*RML2*	0.9	4
YDR174W	*HMO1*	1.6	4
YIL053W	*RHR2*	1.1	4
YKR085C	*MRPL20*		4
YLR068W	*FYV7*	1.5	3.9
YHR064C	*SSZ1*	1.1	3.9
YPL106C	*SSE1*		3.9
YNL003C	*PET8*	0.9	3.85
YOR201C	*MRM1*		3.85
YJL120W		0.7	3.8
YPR116W		0.9	3.8
YGR219W			3.8
YMR293C		0.7	3.7
YML061C	*PIF1*	0.9	3.7
YDR065W		1.15	3.7
YGL246C	*RAI1*		3.7
YPL005W	*AEP3*		3.7
YDR115W			3.65
YLR382C	*NAM2*	0.8	3.6
YHR067W	*HTD2*	1	3.55
YOR096W	*RPS7A*	1.65	3.55
YBR268W	*MRPL37*	0.9	3.55
YER017C	*AFG3*		3.55
YIL060W			3.55
YDR157W		0.9	3.5
YLR048W	*RPS0B*	1.4	3.4

YMR267W	*PPA2*		3.4
YNL284C	*MRPL10*	0.7	3.35
YDR268W	*MSW1*	1.2	3.3
YDL202W	*MRPL11*	1	3.25
YKL169C			3.2
YGL076C	*RPL7A*	1.1	3.15
YBL090W	*MRP21*		3.15
YDR359C	*VID21*		3.1
YFL001W	*DEG1*		3.1
YGL219C	*MDM34*		3.1
YOL023W	*IFM1*		3.1
YBR026C	*ETR1*	0.6	3.05
YGL064C	*MRH4*		3.05
YGR240C	*PFK1*	1	3
YNR012W	*URK1*	1	3
YER077C		0.6	2.95
YPR024W	*YME1*		2.9
YLR192C	*HCR1*	1.2	2.85
YDL020C	*RPN4*	0.9	2.8
YJL204C	*RCY1*	0.9	2.8
YPL178W	*CBC2*		2.8
YFR001W	*LOC1*	1.3	2.8
YGR159C	*NSR1*	1.3	2.8
YEL033W		1.05	2.75
YOR078W	*BUD21*	1.3	2.75
YIL110W	*MNI1*	1	2.75
YDR241W	*BUD26*	0.8	2.65
YJR118C	*ILM1*	1.1	2.65
YBR168W	*PEX32*	1.4	2.65
YPL125W	*KAP120*		2.65
YOR183W	*FYV12*	1	2.6
YGR102C			2.6
YJR049C	*UTR1*		2.6
YHR011W	*DIA4*	0.8	2.6
YDR136C	*VPS61*		2.6
YDR020C		1	2.55
YML122C		1.1	2.55
YIL047C	*SYG1*	1	2.55
YJR032W	*CPR7*	1.4	2.5
YDR393W	*SHE9*		2.5
YIL017C	*VID28*		2.5
YOR211C	*MGM1*		2.45
YKL016C	*ATP7*		2.45
YHR041C	*SRB2*		2.45
YBR163W	*DEM1*	0.6	2.4
YDR337W	*MRPS28*		2.4
YFL023W	*BUD27*		2.4
YHR010W	*RPL27A*	0.95	2.35
YGL088W		0.9	2.35
YML095C-A			2.35
YKL138C	*MRPL31*		2.35

YMR066W	*SOV1*	0.75	2.3
YOL009C	*MDM12*	0.85	2.3
YLL006W	*MMM1*	0.9	2.3
YMR142C	*RPL13B*	1.1	2.3
YJL046W			2.3
YGL072C			2.3
YOR196C	*LIP5*		2.3
YPL047W	*SGF11*		2.3
YMR098C			2.25
YMR312W	*ELP6*		2.25
YHR194W	*MDM31*	0.6	2.2
YJL124C	*LSM1*		2.2
YKL110C	*KTI12*	0.9	2.15
YHR038W	*RRF1*		2.15
YPR131C	*NAT3*	0.9	2.1
YGL127C	*SOH1*		2.1
YDL056W	*MBP1*		2.1
YAL020C	*ATS1*		2.1
YKL134C	*39356*	0.8	2.1
YPR132W	*RPS23B*	0.8	2.1
YJR034W	*PET191*		2.1
YGL105W	*ARC1*		2.1
YOR001W	*RRP6*	0.9	2.05
YBR106W	*PHO88*		2.05
YDR350C	*ATP22*		2.05
YER074W	*RPS24A*	0.95	2
YDR126W	*SWF1*		2
YCR094W	*CDC50*	0.9	2
YPL157W	*TGS1*		2
YLR061W	*RPL22A*		1.95
YNL005C	*MRP7*		1.95
YNL184C			1.95
YLR410W	*VIP1*		1.95
YBR181C	*RPS6B*	0.7	1.95
YCR028C-A	*RIM1*		1.95
YGR257C	*MTM1*		1.95
YLR368W	*MDM30*		1.9
YLR287C-A	*RPS30A*		1.9
YBR266C	*SLM6*		1.9
YAL026C	*DRS2*		1.9
YOR375C	*GDH1*		1.9
YPR074C	*TKL1*		1.9
YDR463W	*STP1*		1.9
YPL090C	*RPS6A*	0.6	1.9
YOR241W	*MET7*		1.9
YDR083W	*RRP8*	0.75	1.85
YIL098C	*FMC1*		1.85
YFL018C	*LPD1*		1.85
YPL102C			1.85
YCL058C	*FYV5*	0.6	1.85
YLR052W	*IES3*		1.85

YHR162W		0.8	1.8
YGR160W		0.95	1.8
YGL136C	*MRM2*		1.8
YNL071W	*LAT1*		1.8
YGR118W	*RPS23A*	0.7	1.8
YJL136C	*RPS21B*	0.7	1.8
YPR066W	*UBA3*		1.8
YDL083C	*RPS16B*		1.8
YIL015C-A			1.8
YDL082W	*RPL13A*		1.8
YOR039W	*CKB2*		1.75
YDR237W	*MRPL7*		1.75
YDR450W	*RPS18A*	0.8	1.7
YIR026C	*YVH1*	0.9	1.7
YER156C			1.7
YGR162W	*TIF4631*	0.9	1.7
YBR221C	*PDB1*		1.7
YOL100W	*PKH2*		1.7
YMR282C	*AEP2*		1.65
YER139C			1.65
YMR064W	*AEP1*		1.65
YDR194C	*MSS116*		1.65
YGR220C	*MRPL9*		1.65
YMR188C	*MRPS17*	0.5	1.6
YHL011C	*PRS3*		1.6
YNL271C	*BNI1*		1.6
YJR145C	*RPS4A*		1.6
YNL069C	*RPL16B*		1.6
YLR423C	*ATG17*		1.6
YDL151C	*BUD30*		1.6
YLR262C	*YPT6*		1.6
YNL294C	*RIM21*		1.6
YOR246C			1.55
YPL101W	*ELP4*		1.55
YER081W	*SER3*		1.55
YOR061W	*CKA2*		1.55
YGL080W	*FMP37*		1.55
YLR420W	*URA4*		1.55
YLR264W	*RPS28B*		1.55
YGL147C	*RPL9A*		1.55
YIL093C	*RSM25*		1.55
YBR048W	*RPS11B*		1.55
YJL063C	*MRPL8*		1.5
YNL199C	*GCR2*		1.5
YAL044C	*GCV3*		1.5
YML026C	*RPS18B*		1.5
YGR163W	*GTR2*		1.5
YBR162C	*TOS1*		1.5
YNL096C	*RPS7B*	0.7	1.45
YHR034C	*PIH1*		1.45
YMR072W	*ABF2*		1.45

YDR377W	*ATP17*		1.45
YNL141W	*AAH1*		1.45
YPR166C	*MRP2*		1.45
YPL214C	*THI6*		1.45
YGL019W	*CKB1*		1.45
YNL177C	*MRPL22*		1.4
YIR005W	*IST3*		1.4
YGR135W	*PRE9*		1.4
YKR024C	*DBP7*		1.4
YIL041W	*GVP36*		1.4
YOR150W	*MRPL23*		1.4
YPL148C	*PPT2*		1.4
YHL027W	*RIM101*		1.4
YOL001W	*PHO80*		1.4
YPR051W	*MAK3*		1.4
YML121W	*GTR1*		1.4
YGL253W	*HXK2*		1.4
YGR101W	*PCP1*		1.4
YMR099C		0.85	1.35
YOR274W	*MOD5*		1.35
YLR021W	*IRC25*		1.35
YDR477W	*SNF1*		1.35
YDR405W	*MRP20*		1.35
YLR062C	*BUD28*		1.35
YDR516C	*EMI2*		1.35
YMR089C	*YTA12*		1.3
YDR363W-A	*SEM1*	0.7	1.3
YGR272C		0.8	1.3
YNL091W	*NST1*	0.8	1.3
YOR167C	*RPS28A*		1.3
YOR182C	*RPS30B*		1.3
YHR081W	*LRP1*		1.3
YML013W	*SEL1*		1.3
YOR006C			1.3
YGR200C	*ELP2*		1.3
YKL074C	*MUD2*		1.3
YLR357W	*RSC2*		1.3
YKR020W	*VPS51*		1.3
YPL065W	*VPS28*		1.3
YPL226W	*NEW1*		1.3
YGR255C	*COQ6*		1.3
YDR360W	*OPI7*		1.3
YIL077C			1.3
YPL029W	*SUV3*		1.3
YOR030W	*DFG16*		1.25
YDL090C	*RAM1*	0.7	1.25
YKR074W			1.25
YBR077C	*SLM4*		1.25
YLR200W	*YKE2*		1.25
YHR203C	*RPS4B*		1.25
YOR271C	*FSF1*		1.25

YOL095C	*HMI1*		1.25
YLR014C	*PPR1*		1.25
YPR101W	*SNT309*	0.95	1.2
YOL121C	*RPS19A*	0.9	1.2
YBL071C		0.8	1.2
YDL032W			1.2
YOR275C	*RIM20*		1.2
YGL046W			1.2
YDL081C	*RPP1A*		1.2
YBL024W	*NCL1*		1.2
YBR267W	*REI1*	0.6	1.2
YNL054W	*VAC7*		1.2
YJL062W	*LAS21*		1.2
YGL066W	*SGF73*		1.2
YMR216C	*SKY1*		1.2
YDR512C	*EMI1*		1.2
YER164W	*CHD1*		1.2
YNL079C	*TPM1*		1.2
YOR043W	*WHI2*		1.2
YGL213C	*SKI8*		1.2
YER040W	*GLN3*		1.2
YCL037C	*SRO9*		1.2
YER116C	*SLX8*		1.2
YOR270C	*VPH1*		1.2
YGL129C	*RSM23*		1.2
YGR165W	*MRPS35*		1.2
YKR057W	*RPS21A*		1.2
YEL054C	*RPL12A*		1.2
YCR077C	*PAT1*		1.2
YLR388W	*RPS29A*		1.2
YJR055W	*HIT1*	0.8	1.15
YBR058C	*UBP14*		1.15
YLR384C	*IKI3*		1.15
YPL173W	*MRPL40*		1.15
YNL120C		0.8	1.15
YPR189W	*SKI3*	0.7	1.15
YMR063W	*RIM9*		1.15
YLR369W	*SSQ1*		1.15
YGR122W			1.15
YHR147C	*MRPL6*		1.15
YNL148C	*ALF1*		1.15
YKR007W	*MEH1*		1.15
YER141W	*COX15*		1.15
YMR230W	*RPS10B*		1.15
YPL086C	*ELP3*	0.8	1.1
YBR272C	*HSM3*		1.1
YGL035C	*MIG1*		1.1
YNL119W	*NCS2*	0.8	1.1
YPL144W			1.1
YLR139C	*SLS1*		1.1
YGR259C			1.1

YMR258C			1.1
YLR239C	*LIP2*		1.1
YPR106W	*ISR1*		1.1
YLR074C	*BUD20*		1.1
YPR130C			1.1
YJL208C	*NUC1*		1.1
YNL227C	*JJJ1*		1.1
YGR182C			1.1
YGL043W	*DST1*		1.1
YPR099C			1.1
YDR024W	*FYV1*		1.1
YDR462W	*MRPL28*		1.1
YMR100W	*MUB1*		1.1
YPL213W	*LEA1*		1.1
YPR070W	*MED1*		1.05
YHR193C	*EGD2*		1.05
YNL252C	*MRPL17*		1.05
YLR185W	*RPL37A*		1.05
YHR100C			1.05
YOR295W	*UAF30*		1.05
YGR214W	*RPS0A*	0.7	1.05
YKR023W			1.05
YJL023C	*PET130*		1.05
YDR204W	*COQ4*		1.05
YDR447C	*RPS17B*		1.05
YGL214W			1.05
YHR066W	*SSF1*		1.05
YHR116W	*COX23*		1.05
YIL006W	*YIA6*		1.05
YIL076W	*SEC28*		1.05
YBR069C	*TAT1*		1.05
YPL097W	*MSY1*		1
YMR287C	*DSS1*		1
YER110C	*KAP123*	0.9	1
YOL041C	*NOP12*		1
YJL149W			1
YPL270W	*MDL2*		1
YLR201C	*COQ9*		1
YNR020C			1
YML128C	*MSC1*		1
YBR044C	*TCM62*		1
YNL055C	*POR1*		1
YMR116C	*ASC1*		1
YOR125C	*CAT5*		1
YDR322W	*MRPL35*		1
YJL098W	*SAP185*		1
YLR024C	*UBR2*		1
YLR391W			1
YNL226W			1
YCR031C	*RPS14A*		1
YDR048C		0.8	1

YGR192C	*TDH3*		1
YOR330C	*MIP1*		1
YPL061W	*ALD6*		1
YGL163C	*RAD54*		1
YGR076C	*MRPL25*		1
YNL228W			0.95
YGR206W	*MVB12*		0.95
YJL135W			0.95
YHR051W	*COX6*		0.95
YOR376W			0.95
YLR099C	*ICT1*		0.95
YPR057W	*BRR1*		0.95
YNL248C	*RPA49*	0.75	0.9
YJL150W			0.9
YJL179W	*PFD1*		0.9
YIL133C	*RPL16A*		0.9
YDR385W	*EFT2*		0.9
YDR025W	*RPS11A*		0.9
YGR081C	*SLX9*		0.9
YJL141C	*YAK1*		0.9
YNL121C	*TOM70*		0.9
YAR014C	*BUD14*		0.9
YPR100W	*MRPL51*		0.9
YDL023C			0.9
YIL005W	*EPS1*		0.9
YLR202C			0.9
YLR448W	*RPL6B*	0.8	0.9
YEL013W	*VAC8*		0.9
YNL198C			0.9
YPR018W	*RLF2*		0.9
YPR069C	*SPE3*		0.9
YDL136W	*RPL35B*		0.9
YMR228W	*MTF1*		0.9
YPL062W			0.9
YBR290W	*BSD2*		0.9
YIL028W			0.9
YDR297W	*SUR2*		0.85
YDR332W	*IRC3*		0.85
YMR075C-A			0.85
YOR309C			0.85
YKL040C	*NFU1*		0.85
YOR135C	*IRC14*		0.85
YLR180W	*SAM1*		0.8
YGR260W	*TNA1*		0.8
YJR148W	*BAT2*		0.8
YBL080C	*PET112*		0.8
YPL089C	*RLM1*		0.8
YOR355W	*GDS1*		0.8
YPL037C	*EGD1*		0.8
YNL215W	*IES2*		0.8
YNR036C			0.8

YIL095W	*PRK1*		0.8
YGL031C	*RPL24A*		0.8
YLR237W	*THI7*		0.8
YOR136W	*IDH2*		0.8
YER153C	*PET122*		0.8
YMR075W	*RCO1*		0.8
YML014W	*TRM9*		0.8
YOR076C	*SKI7*		0.8
YJL134W	*LCB3*		0.8
YNL023C	*FAP1*		0.8
YBR099C			0.8
YER174C	*GRX4*		0.8
YJL133W	*MRS3*		0.8
YGR187C	*HGH1*		0.8
YJL168C	*SET2*		0.8
YIL069C	*RPS24B*		0.8
YDR073W	*SNF11*		0.8
YLR111W			0.8
YOR358W	*HAP5*		0.8
YGL143C	*MRF1*		0.8
YJR066W	*TOR1*		0.8
YJL159W	*HSP150*		0.75
YHR103W	*SBE22*		0.75
YER058W	*PET117*		0.7
YJR059W	*PTK2*		0.7
YER154W	*OXA1*		0.7
YMR154C	*RIM13*		0.7
YML073C	*RPL6A*		0.7
YML024W	*RPS17A*		0.7
YGR150C			0.7
YMR241W	*YHM2*		0.7
YNL288W	*CAF40*		0.7
YPL099C	*FMP14*		0.7
YOL068C	*HST1*		0.7
YLR006C	*SSK1*		0.7
YMR313C	*TGL3*		0.7
YKL007W	*CAP1*		0.7
YDL094C			0.7
YLR207W	*HRD3*		0.7
YMR223W	*UBP8*		0.7
YJR082C	*EAF6*		0.7
YJL164C	*TPK1*		0.7
YKL206C	*ADD66*		0.7
YJR005W	*APL1*		0.7
YLR144C	*ACF2*		0.7
YGL034C			0.7
YIL016W	*SNL1*		0.65
YMR036C	*MIH1*		0.6
YBR114W	*RAD16*		0.6
YHR073W	*OSH3*		0.6
YIL050W	*PCL7*		0.6

YIL065C	*FIS1*		0.6
YLR289W	*GUF1*		0.6
YJL117W	*PHO86*	2.8	
YCR028C	*FEN2*	1.9	
YCR047C	*BUD23*	1.6	
YML035C	*AMD1*	1.45	
YPL161C	*BEM4*	1.45	
YOR209C	*NPT1*	1.45	
YPR067W	*ISA2*	1.4	
YGL135W	*RPL1B*	1.35	
YLL027W	*ISA1*	1.3	
YML097C	*VPS9*	1.25	
YOR221C	*MCT1*	1.25	
YKR026C	*GCN3*	1.2	
YLR177W		1.2	
YPL271W	*ATP15*	1.2	
YEL045C		1.2	
YKL009W	*MRT4*	1.2	
YDL198C	*GGC1*	1.15	
YOL108C	*INO4*	1.15	
YKL170W	*MRPL38*	1.1	
YIR033W	*MGA2*	1.1	
YJL075C	*APQ13*	1.1	
YHR120W	*MSH1*	1.05	
YFR009W	*GCN20*	1.05	
YJR040W	*GEF1*	1.05	
YPL180W	*TCO89*	1	
YLR439W	*MRPL4*	1	
YMR014W	*BUD22*	1	
YLR091W		1	
YMR123W	*PKR1*	1	
YNL064C	*YDJ1*	1	
YGR157W	*CHO2*	1	
YHR059W	*FYV4*	0.95	
YLR403W	*SFP1*	0.9	
YDR161W		0.9	
YPL104W	*MSD1*	0.9	
YMR058W	*FET3*	0.9	
YER145C	*FTR1*	0.9	
YJL121C	*RPE1*	0.9	
YCR063W	*BUD31*	0.9	
YDL160C	*DHH1*	0.9	
YDR175C	*RSM24*	0.9	
YDR123C	*INO2*	0.85	
YPL002C	*SNF8*	0.8	
YKL003C	*MRP17*	0.8	
YJL102W	*MEF2*	0.8	
YDR296W	*MHR1*	0.8	
YAL023C	*PMT2*	0.8	
YOR089C	*VPS21*	0.8	
YPR047W	*MSF1*	0.8	

YFL036W	*RPO41*	0.8	
YOR235W	*IRC13*	0.75	
YLR214W	*FRE1*	0.7	
YDR195W	*REF2*	0.7	
YKR084C	*HBS1*	0.7	
YDR049W		0.7	
YDR269C		0.7	
