# Supplementary material for: Genome-Wide Functional Profiling Identifies Genes and Processes Important for Zinc-Limited Growth of Saccharomyces cerevisiae
Source: PLoS Genet. 2012 Jun 7;8(6):e1002699. doi: 10.1371/journal.pgen.1002699 (PMC3369956; doi:10.1371/journal.pgen.1002699)
Supplement: Table S3 — Yeast gene deletion mutants unaffected in low zinc. Deletion mutants tested in the functional profiling analysis but not identified as sensitive or resistant to low zinc are listed. (PDF) [file pgen.1002699.s004.pdf]

Supplemental Table 3. Unaffected mutants.

ORF	Gene
YAL002W	VPS8
YAL004W	
YAL005C	SSA1
YAL007C	ERP2
YAL008W	FUN14
YAL011W	SWC3
YAL014C	SYN8
YAL015C	NTG1
YAL016W	TPD3
YAL017W	PSK1
YAL018C	
YAL019W	FUN30
YAL021C	CCR4
YAL022C	FUN26
YAL024C	LTE1
YAL027W	
YAL028W	FRT2
YAL029C	MYO4
YAL030W	SNC1
YAL031C	GIP4
YAL034C	FUN19
YAL035W	FUN12
YAL036C	RBG1
YAL037W	
YAL039C	CYC3
YAL042W	ERV46
YAL043C-A	
YAL045C	
YAL046C	
YAL047C	SPC72
YAL048C	GEM1
YAL049C	
YAL051W	OAF1
YAL053W	FLC2
YAL054C	ACS1
YAL056W	GPB2
YAL058C-A	
YAL058W	CNE1
YAL059W	ECM1
YAL060W	BDH1
YAL061W	
YAL062W	GDH3
YAL064C-A	
YAL065C	
YAL066W	
YAL067C	SEO1
YAL068C	PAU8
YAR002C-A	ERP1
YAR002W	NUP60
YAR003W	SWD1
YAR018C	KIN3
YAR020C	PAU7
YAR023C	
YAR027W	UIP3
YAR028W	
YAR029W	
YAR030C	
YAR031W	PRM9
YAR035W	YAT1
YAR037W	
YAR040C	
YAR042W	SWH1
YAR043C	
YAR044W	
YAR047C	
YAR050W	FLO1
YBL001C	ECM15
YBL002W	HTB2
YBL003C	HTA2
YBL005W	PDR3
YBL006C	LDB7
YBL007C	SLA1
YBL008W	HIR1
YBL009W	ALK2
YBL010C	
YBL011W	SCT1
YBL012C	
YBL013W	FMT1
YBL015W	ACH1
YBL017C	PEP1
YBL019W	APN2
YBL022C	PIM1
YBL027W	RPL19B
YBL028C	
YBL029W	
YBL032W	HEK2
YBL033C	RIB1
YBL036C	
YBL037W	APL3
YBL042C	FUI1

YBL043W	ECM13
YBL044W	
YBL045C	COR1
YBL047C	EDE1
YBL048W	
YBL049W	MOH1
YBL051C	PIN4
YBL052C	SAS3
YBL053W	
YBL054W	
YBL055C	
YBL057C	PTH2
YBL058W	SHP1
YBL059W	
YBL060W	
YBL063W	KIP1
YBL064C	PRX1
YBL065W	
YBL066C	SEF1
YBL068W	PRS4
YBL069W	AST1
YBL070C	
YBL072C	RPS8A
YBL075C	SSA3
YBL079W	NUP170
YBL081W	
YBL085W	BOI1
YBL086C	
YBL087C	RPL23A
YBL088C	TEL1
YBL089W	AVT5
YBL091C-A	SCS22
YBL093C	ROX3
YBL094C	
YBL095W	
YBL096C	
YBL098W	BNA4
YBL099W	ATP1
YBL100C	
YBL101C	ECM21
YBL102W	SFT2
YBL104C	
YBL106C	SRO77
YBL107C	
YBR001C	NTH2
YBR005W	RCR1
YBR006W	UGA2
YBR007C	DSF2
YBR008C	FLR1
YBR010W	HHT1
YBR012C	
YBR013C	
YBR014C	
YBR016W	
YBR018C	GAL7
YBR019C	GAL10
YBR020W	GAL1
YBR022W	POA1
YBR024W	SCO2
YBR025C	
YBR028C	
YBR029C	CDS1
YBR031W	RPL4A
YBR032W	
YBR033W	EDS1
YBR034C	HMT1
YBR035C	PDX3
YBR037C	SCO1
YBR038W	CHS2
YBR040W	FIG1
YBR042C	
YBR043C	QDR3
YBR045C	GIP1
YBR046C	ZTA1
YBR047W	FMP23
YBR050C	REG2
YBR051W	
YBR052C	RFS1
YBR053C	
YBR054W	YRO2
YBR056W	
YBR057C	MUM2
YBR059C	AKL1
YBR062C	
YBR063C	
YBR064W	
YBR065C	ECM2
YBR066C	NRG2
YBR067C	TIP1
YBR068C	BAP2
YBR071W	
YBR072W	HSP26
YBR073W	RDH54
YBR074W	
YBR075W	
YBR076W	ECM8

YBR083W	TEC1
YBR084C-A	RPL19A
YBR084W	MIS1
YBR090C	
YBR090C-A	
YBR092C	PHO3
YBR093C	PHO5
YBR094W	PBY1
YBR098W	MMS4
YBR100W	
YBR101C	FES1
YBR104W	YMC2
YBR105C	VID24
YBR107C	IML3
YBR108W	
YBR112C	CYC8
YBR113W	
YBR116C	
YBR119W	MUD1
YBR120C	CBP6
YBR125C	PTC4
YBR126C	TPS1
YBR127C	VMA2
YBR128C	ATG14
YBR129C	OPY1
YBR130C	SHE3
YBR133C	HSL7
YBR134W	
YBR137W	
YBR138C	
YBR139W	
YBR141C	
YBR144C	
YBR145W	ADH5
YBR146W	MRPS9
YBR147W	
YBR148W	YSW1
YBR149W	ARA1
YBR150C	TBS1
YBR151W	APD1
YBR157C	ICS2
YBR158W	AMN1
YBR159W	IFA38
YBR161W	CSH1
YBR162W-A	YSY6
YBR164C	ARL1
YBR169C	SSE2
YBR170C	NPL4
YBR172C	SMY2
YBR173C	UMP1
YBR174C	
YBR175W	SWD3
YBR176W	ECM31
YBR177C	EHT1
YBR178W	
YBR179C	FZO1
YBR180W	DTR1
YBR182C	SMP1
YBR183W	YPC1
YBR184W	
YBR185C	MBA1
YBR186W	PCH2
YBR187W	GDT1
YBR188C	NTC20
YBR189W	RPS9B
YBR191W	RPL21A
YBR194W	SOY1
YBR195C	MSI1
YBR197C	
YBR199W	KTR4
YBR200W	BEM1
YBR201W	DER1
YBR203W	COS111
YBR204C	
YBR205W	KTR3
YBR206W	
YBR207W	FTH1
YBR208C	DUR1,2
YBR210W	ERV15
YBR212W	NGR1
YBR213W	MET8
YBR214W	SDS24
YBR215W	HPC2
YBR218C	PYC2
YBR219C	
YBR220C	
YBR222C	PCS60
YBR223C	TDP1
YBR224W	
YBR225W	
YBR226C	
YBR228W	SLX1
YBR230C	OM14
YBR232C	
YBR233W	PBP2
YBR235W	

YBR238C	
YBR239C	
YBR240C	THI2
YBR241C	
YBR242W	
YBR244W	GPX2
YBR245C	ISW1
YBR246W	
YBR248C	HIS7
YBR250W	SPO23
YBR251W	MRPS5
YBR258C	SHG1
YBR259W	
YBR261C	
YBR262C	FMP51
YBR264C	YPT10
YBR269C	FMP21
YBR270C	BIT2
YBR271W	
YBR273C	UBX7
YBR274W	CHK1
YBR275C	RIF1
YBR276C	PPS1
YBR279W	PAF1
YBR280C	SAF1
YBR281C	DUG2
YBR282W	MRPL27
YBR283C	SSH1
YBR284W	
YBR285W	
YBR286W	APE3
YBR287W	ZSP1
YBR292C	
YBR293W	VBA2
YBR294W	SUL1
YBR295W	PCA1
YBR296C	PHO89
YBR297W	MAL33
YBR299W	MAL32
YBR300C	
YBR301W	DAN3
YCL001W	RER1
YCL002C	
YCL006C	
YCL007C	
YCL008C	STP22
YCL009C	ILV6
YCL010C	SGF29
YCL011C	GBP2
YCL012W	
YCL013W	
YCL014W	BUD3
YCL016C	DCC1
YCL022C	
YCL023C	
YCL024W	KCC4
YCL025C	AGP1
YCL026C	
YCL026C-A	FRM2
YCL027W	FUS1
YCL028W	RNQ1
YCL030C	HIS4
YCL032W	STE50
YCL033C	
YCL034W	LSB5
YCL035C	GRX1
YCL036W	GFD2
YCL039W	GID7
YCL040W	GLK1
YCL042W	
YCL044C	MGR1
YCL045C	
YCL046W	
YCL047C	
YCL048W	SPS22
YCL049C	
YCL050C	APA1
YCL051W	LRE1
YCL055W	KAR4
YCL056C	
YCL057W	PRD1
YCL060C	
YCL061C	MRC1
YCL062W	
YCL063W	VAC17
YCL064C	CHA1
YCL069W	VBA3
YCL074W	
YCL075W	
YCL076W	
YCR001W	
YCR004C	YCP4
YCR005C	CIT2
YCR006C	
YCR007C	
YCR010C	ADY2

YCR014C	POL4
YCR015C	
YCR016W	
YCR017C	CWH43
YCR019W	MAK32
YCR020C	PET18
YCR020C-A	MAK31
YCR020W-B	HTL1
YCR021C	HSP30
YCR022C	
YCR023C	
YCR024C	SLM5
YCR025C	
YCR027C	RHB1
YCR030C	SYP1
YCR037C	PHO87
YCR043C	
YCR048W	ARE1
YCR049C	
YCR051W	
YCR053W	THR4
YCR059C	YIH1
YCR061W	
YCR062W	
YCR065W	HCM1
YCR066W	RAD18
YCR067C	SED4
YCR069W	CPR4
YCR071C	IMG2
YCR073C	SSK22
YCR073W-A	SOL2
YCR075C	ERS1
YCR079W	PTC6
YCR082W	AHC2
YCR083W	TRX3
YCR085W	
YCR088W	ABP1
YCR089W	FIG2
YCR090C	
YCR091W	KIN82
YCR092C	MSH3
YCR095C	OCA4
YCR098C	GIT1
YCR099C	
YCR100C	
YCR101C	
YCR102C	
YCR102W-A	
YCR105W	ADH7
YCR106W	RDS1
YCR107W	AAD3
YDL001W	RMD1
YDL002C	NHP10
YDL006W	PTC1
YDL009C	
YDL010W	
YDL011C	
YDL012C	
YDL013W	HEX3
YDL018C	ERP3
YDL019C	OSH2
YDL021W	GPM2
YDL022W	GPD1
YDL024C	DIA3
YDL025C	
YDL026W	
YDL027C	
YDL033C	SLM3
YDL034W	
YDL035C	GPR1
YDL036C	PUS9
YDL037C	BSC1
YDL038C	
YDL039C	PRM7
YDL041W	
YDL045W-A	MRP10
YDL046W	NPC2
YDL047W	SIT4
YDL048C	STP4
YDL049C	KNH1
YDL050C	
YDL051W	LHP1
YDL052C	SLC1
YDL053C	PBP4
YDL054C	MCH1
YDL059C	RAD59
YDL061C	RPS29B
YDL062W	
YDL063C	
YDL067C	COX9
YDL068W	
YDL069C	CBS1
YDL070W	BDF2
YDL071C	IRC1
YDL072C	YET3
YDL073W	

YDL075W	RPL31A
YDL076C	RXT3
YDL077C	VAM6
YDL078C	MDH3
YDL079C	MRK1
YDL080C	THI3
YDL085W	NDE2
YDL086W	
YDL088C	ASM4
YDL089W	
YDL091C	UBX3
YDL093W	PMT5
YDL095W	PMT1
YDL096C	OPI6
YDL099W	BUG1
YDL100C	GET3
YDL101C	DUN1
YDL104C	QRI7
YDL106C	PHO2
YDL107W	MSS2
YDL109C	
YDL110C	TMA17
YDL112W	TRM3
YDL113C	ATG20
YDL114W	
YDL115C	IWR1
YDL116W	NUP84
YDL117W	CYK3
YDL121C	
YDL122W	UBP1
YDL123W	SNA4
YDL124W	
YDL127W	PCL2
YDL128W	VCX1
YDL129W	
YDL130W	RPP1B
YDL130W-A	STF1
YDL131W	LYS21
YDL133C-A	RPL41B
YDL134C	PPH21
YDL134C-A	
YDL135C	RDI1
YDL137W	ARF2
YDL138W	RGT2
YDL142C	CRD1
YDL144C	
YDL146W	LDB17
YDL149W	ATG9
YDL154W	MSH5
YDL155W	CLB3
YDL156W	
YDL157C	
YDL161W	ENT1
YDL167C	NRP1
YDL168W	SFA1
YDL169C	UGX2
YDL170W	UGA3
YDL171C	GLT1
YDL174C	DLD1
YDL175C	AIR2
YDL176W	
YDL177C	
YDL178W	DLD2
YDL179W	PCL9
YDL182W	LYS20
YDL183C	
YDL184C	RPL41A
YDL185W	TFP1
YDL186W	
YDL187C	
YDL188C	PPH22
YDL189W	RBS1
YDL190C	UFD2
YDL191W	RPL35A
YDL192W	ARF1
YDL194W	SNF3
YDL197C	ASF2
YDL199C	
YDL200C	MGT1
YDL201W	TRM8
YDL204W	RTN2
YDL206W	
YDL210W	UGA4
YDL211C	
YDL213C	NOP6
YDL214C	PRR2
YDL215C	GDH2
YDL216C	RRI1
YDL218W	
YDL219W	DTD1
YDL222C	FMP45
YDL223C	HBT1
YDL224C	WHI4
YDL225W	SHS1
YDL227C	HO
YDL229W	SSB1

YDL230W	PTP1
YDL231C	BRE4
YDL232W	OST4
YDL233W	
YDL234C	GYP7
YDL236W	PHO13
YDL237W	
YDL238C	GUD1
YDL239C	ADY3
YDL240W	LRG1
YDL241W	
YDL242W	
YDL243C	AAD4
YDR001C	NTH1
YDR003W	RCR2
YDR004W	RAD57
YDR007W	TRP1
YDR008C	
YDR009W	GAL3
YDR010C	
YDR011W	SNQ2
YDR014W	RAD61
YDR015C	
YDR017C	KCS1
YDR018C	
YDR019C	GCV1
YDR022C	CIS1
YDR026C	
YDR027C	VPS54
YDR029W	
YDR030C	RAD28
YDR031W	MIC14
YDR032C	PST2
YDR033W	MRH1
YDR034C	LYS14
YDR035W	ARO3
YDR036C	EHD3
YDR042C	
YDR046C	BAP3
YDR051C	
YDR055W	PST1
YDR056C	
YDR057W	YOS9
YDR058C	TGL2
YDR059C	UBC5
YDR061W	
YDR063W	
YDR066C	
YDR067C	OCA6
YDR068W	DOS2
YDR069C	DOA4
YDR070C	FMP16
YDR072C	IPT1
YDR074W	TPS2
YDR075W	PPH3
YDR076W	RAD55
YDR077W	SED1
YDR078C	SHU2
YDR079W	PET100
YDR084C	TVP23
YDR085C	AFR1
YDR089W	
YDR090C	
YDR092W	UBC13
YDR093W	DNF2
YDR094W	
YDR095C	
YDR096W	GIS1
YDR097C	MSH6
YDR098C	GRX3
YDR099W	BMH2
YDR100W	TVP15
YDR101C	ARX1
YDR102C	
YDR104C	SPO71
YDR105C	TMS1
YDR107C	
YDR109C	
YDR110W	FOB1
YDR111C	ALT2
YDR114C	
YDR116C	MRPL1
YDR117C	TMA64
YDR119W	
YDR120C	TRM1
YDR121W	DPB4
YDR122W	KIN1
YDR124W	
YDR125C	ECM18
YDR128W	
YDR129C	SAC6
YDR130C	FIN1
YDR131C	
YDR132C	
YDR133C	
YDR134C	

YDR135C	YCF1
YDR138W	HPR1
YDR139C	RUB1
YDR142C	PEX7
YDR143C	SAN1
YDR144C	MKC7
YDR146C	SWI5
YDR147W	EKI1
YDR148C	KGD2
YDR151C	CTH1
YDR152W	GIR2
YDR153C	ENT5
YDR154C	
YDR155C	CPR1
YDR156W	RPA14
YDR159W	SAC3
YDR162C	NBP2
YDR163W	CWC15
YDR165W	TRM82
YDR171W	HSP42
YDR176W	NGG1
YDR178W	SDH4
YDR179C	CSN9
YDR179W-A	
YDR181C	SAS4
YDR183W	PLP1
YDR184C	ATC1
YDR185C	
YDR186C	
YDR191W	HST4
YDR192C	NUP42
YDR193W	
YDR197W	CBS2
YDR198C	RKM2
YDR199W	
YDR200C	VPS64
YDR202C	RAV2
YDR203W	
YDR206W	EBS1
YDR209C	
YDR210W	
YDR213W	UPC2
YDR214W	AHA1
YDR215C	
YDR216W	ADR1
YDR217C	RAD9
YDR218C	SPR28
YDR219C	MFB1
YDR220C	
YDR221W	GTB1
YDR222W	
YDR223W	CRF1
YDR226W	ADK1
YDR229W	IVY1
YDR230W	
YDR231C	COX20
YDR233C	RTN1
YDR239C	
YDR242W	AMD2
YDR244W	PEX5
YDR245W	MNN10
YDR247W	VHS1
YDR248C	
YDR249C	
YDR250C	
YDR251W	PAM1
YDR252W	BTT1
YDR255C	RMD5
YDR256C	CTA1
YDR257C	SET7
YDR258C	HSP78
YDR259C	YAP6
YDR260C	SWM1
YDR261C	EXG2
YDR262W	
YDR263C	DIN7
YDR264C	AKR1
YDR266C	
YDR270W	CCC2
YDR271C	
YDR272W	GLO2
YDR273W	DON1
YDR274C	
YDR275W	BSC2
YDR277C	MTH1
YDR278C	
YDR279W	RNH202
YDR282C	
YDR283C	GCN2
YDR284C	DPP1
YDR285W	ZIP1
YDR286C	
YDR287W	
YDR291W	HRQ1
YDR293C	SSD1
YDR300C	PRO1

YDR304C	CPR5
YDR305C	HNT2
YDR306C	
YDR307W	
YDR309C	GIC2
YDR310C	SUM1
YDR312W	SSF2
YDR313C	PIB1
YDR314C	RAD34
YDR315C	IPK1
YDR316W	OMS1
YDR317W	HIM1
YDR318W	MCM21
YDR319C	
YDR320C	SWA2
YDR321W	ASP1
YDR323C	PEP7
YDR326C	YSP2
YDR330W	UBX5
YDR333C	
YDR336W	
YDR338C	
YDR340W	
YDR344C	
YDR345C	HXT3
YDR346C	SVF1
YDR349C	YPS7
YDR351W	SBE2
YDR352W	
YDR354W	TRP4
YDR357C	
YDR358W	GGA1
YDR368W	YPR1
YDR369C	XRS2
YDR370C	
YDR371W	CTS2
YDR374C	
YDR375C	BCS1
YDR378C	LSM6
YDR379W	RGA2
YDR380W	ARO10
YDR382W	RPP2B
YDR383C	NKP1
YDR384C	ATO3
YDR386W	MUS81
YDR387C	
YDR389W	SAC7
YDR391C	
YDR399W	HPT1
YDR400W	URH1
YDR401W	
YDR402C	DIT2
YDR403W	DIT1
YDR406W	PDR15
YDR409W	SIZ1
YDR411C	DFM1
YDR415C	
YDR417C	
YDR418W	RPL12B
YDR419W	RAD30
YDR420W	HKR1
YDR421W	ARO80
YDR422C	SIP1
YDR423C	CAD1
YDR428C	
YDR430C	CYM1
YDR431W	
YDR432W	NPL3
YDR433W	
YDR435C	PPM1
YDR436W	PPZ2
YDR438W	THI74
YDR439W	LRS4
YDR440W	DOT1
YDR441C	APT2
YDR442W	
YDR444W	
YDR445C	
YDR446W	ECM11
YDR448W	ADA2
YDR452W	PPN1
YDR453C	TSA2
YDR457W	TOM1
YDR458C	HEH2
YDR459C	PFA5
YDR461W	MFA1
YDR465C	RMT2
YDR467C	
YDR469W	SDC1
YDR470C	UGO1
YDR471W	RPL27B
YDR474C	
YDR476C	
YDR479C	PEX29
YDR480W	DIG2
YDR481C	PHO8

YDR482C	CWC21
YDR483W	KRE2
YDR485C	VPS72
YDR490C	PKH1
YDR491C	
YDR492W	IZH1
YDR493W	FMP36
YDR494W	RSM28
YDR495C	VPS3
YDR497C	ITR1
YDR500C	RPL37B
YDR501W	PLM2
YDR502C	SAM2
YDR503C	LPP1
YDR504C	SPG3
YDR506C	
YDR507C	GIN4
YDR508C	GNP1
YDR509W	
YDR511W	ACN9
YDR513W	GRX2
YDR514C	
YDR515W	SLF1
YDR517W	GRH1
YDR518W	EUG1
YDR519W	FPR2
YDR520C	
YDR521W	
YDR522C	SPS2
YDR523C	SPS1
YDR524C	AGE1
YDR525W-A	SNA2
YDR528W	HLR1
YDR529C	QCR7
YDR530C	APA2
YDR532C	
YDR533C	HSP31
YDR534C	FIT1
YDR535C	
YDR536W	STL1
YDR537C	
YDR538W	PAD1
YDR539W	
YDR540C	IRC4
YDR541C	
YEL003W	GIM4
YEL004W	YEA4
YEL005C	VAB2
YEL006W	YEA6
YEL008W	
YEL009C	GCN4
YEL010W	
YEL011W	GLC3
YEL012W	UBC8
YEL014C	
YEL015W	EDC3
YEL016C	NPP2
YEL017C-A	PMP2
YEL017W	GTT3
YEL018W	EAF5
YEL020C	
YEL023C	
YEL024W	RIP1
YEL025C	
YEL027W	CUP5
YEL028W	
YEL030W	ECM10
YEL036C	ANP1
YEL037C	RAD23
YEL038W	UTR4
YEL039C	CYC7
YEL040W	UTR2
YEL041W	YEF1
YEL043W	
YEL044W	IES6
YEL046C	GLY1
YEL047C	
YEL048C	
YEL049W	PAU2
YEL051W	VMA8
YEL052W	AFG1
YEL053C	MAK10
YEL056W	HAT2
YEL057C	
YEL059W	
YEL060C	PRB1
YEL063C	CAN1
YEL064C	AVT2
YEL065W	SIT1
YEL066W	HPA3
YEL067C	
YEL068C	
YEL071W	DLD3
YER001W	MNN1
YER002W	NOP16
YER004W	FMP52

YER007C-A	TMA20
YER007W	PAC2
YER011W	TIR1
YER014C-A	BUD25
YER019C-A	SBH2
YER019W	ISC1
YER024W	YAT2
YER027C	GAL83
YER028C	MIG3
YER030W	CHZ1
YER031C	YPT31
YER032W	FIR1
YER033C	ZRG8
YER034W	
YER037W	PHM8
YER038W-A	FMP49
YER039C	HVG1
YER039C-A	
YER041W	YEN1
YER042W	MXR1
YER044C	ERG28
YER044C-A	MEI4
YER045C	ACA1
YER046W	SPO73
YER046W-A	
YER047C	SAP1
YER048C	CAJ1
YER049W	TPA1
YER051W	JHD1
YER053C	PIC2
YER055C	HIS1
YER056C	FCY2
YER056C-A	RPL34A
YER057C	HMF1
YER060W	FCY21
YER060W-A	FCY22
YER062C	HOR2
YER063W	THO1
YER064C	
YER065C	ICL1
YER066C-A	
YER066W	
YER067C-A	
YER067W	
YER068W	MOT2
YER071C	
YER073W	ALD5
YER075C	PTP3
YER078C	
YER079W	
YER080W	FMP29
YER083C	GET2
YER084W	
YER085C	
YER087C-A	
YER088C	DOT6
YER089C	PTC2
YER091C-A	
YER092W	IES5
YER093C-A	
YER095W	RAD51
YER096W	SHC1
YER097W	
YER098W	UBP9
YER101C	AST2
YER103W	SSA4
YER106W	MAM1
YER108C	
YER109C	FLO8
YER113C	
YER114C	BOI2
YER115C	SPR6
YER117W	RPL23B
YER118C	SHO1
YER119C	AVT6
YER119C-A	
YER120W	SCS2
YER121W	
YER122C	GLO3
YER123W	YCK3
YER124C	DSE1
YER128W	
YER129W	SAK1
YER130C	
YER131W	RPS26B
YER132C	PMD1
YER134C	
YER135C	
YER137C	
YER140W	
YER142C	MAG1
YER144C	UBP5
YER149C	PEA2
YER150W	SPI1
YER151C	UBP3
YER152C	

YER155C	BEM2
YER158C	
YER162C	RAD4
YER163C	
YER166W	DNF1
YER169W	RPH1
YER170W	ADK2
YER173W	RAD24
YER175C	TMT1
YER176W	ECM32
YER177W	BMH1
YER178W	PDA1
YER179W	DMC1
YER180C	ISC10
YER181C	
YER182W	FMP10
YER183C	FAU1
YER184C	
YER185W	
YER186C	
YER187W	
YER188W	
YFL003C	MSH4
YFL004W	VTC2
YFL006W	
YFL007W	BLM10
YFL010C	WWM1
YFL010W-A	AUA1
YFL011W	HXT10
YFL012W	
YFL013C	IES1
YFL013W-A	
YFL014W	HSP12
YFL015C	
YFL019C	
YFL020C	PAU5
YFL027C	GYP8
YFL028C	CAF16
YFL030W	AGX1
YFL033C	RIM15
YFL034C-A	RPL22B
YFL034W	
YFL035C-B	
YFL040W	
YFL041W	FET5
YFL042C	
YFL046W	FMP32
YFL047W	RGD2
YFL048C	EMP47
YFL049W	SWP82
YFL050C	ALR2
YFL051C	
YFL052W	
YFL053W	DAK2
YFL054C	
YFL055W	AGP3
YFL056C	AAD6
YFL063W	
YFR006W	
YFR007W	
YFR008W	FAR7
YFR010W	UBP6
YFR011C	
YFR012W	
YFR013W	IOC3
YFR014C	CMK1
YFR015C	GSY1
YFR016C	
YFR017C	
YFR018C	
YFR019W	FAB1
YFR020W	
YFR021W	ATG18
YFR022W	ROG3
YFR023W	PES4
YFR024C	
YFR024C-A	LSB3
YFR025C	HIS2
YFR026C	
YFR030W	MET10
YFR031C-A	RPL2A
YFR032C	
YFR032C-A	RPL29
YFR033C	QCR6
YFR034C	PHO4
YFR035C	
YFR036W	CDC26
YFR038W	IRC5
YFR039C	
YFR041C	ERJ5
YFR043C	IRC6
YFR044C	DUG1
YFR045W	
YFR046C	CNN1
YFR047C	BNA6
YFR048W	RMD8

YFR049W	YMR31
YFR053C	HXK1
YFR054C	
YFR055W	IRC7
YFR056C	
YFR057W	
YGL002W	ERP6
YGL004C	RPN14
YGL005C	COG7
YGL006W	PMC1
YGL007W	BRP1
YGL010W	
YGL012W	ERG4
YGL013C	PDR1
YGL014W	PUF4
YGL015C	
YGL016W	KAP122
YGL017W	ATE1
YGL020C	GET1
YGL021W	ALK1
YGL023C	PIB2
YGL025C	PGD1
YGL028C	SCW11
YGL032C	AGA2
YGL033W	HOP2
YGL036W	
YGL037C	PNC1
YGL038C	OCH1
YGL039W	
YGL041C	
YGL042C	
YGL045W	RIM8
YGL050W	TYW3
YGL051W	MST27
YGL053W	PRM8
YGL056C	SDS23
YGL057C	
YGL058W	RAD6
YGL059W	
YGL062W	PYC1
YGL063W	PUS2
YGL067W	NPY1
YGL070C	RPB9
YGL071W	AFT1
YGL077C	HNM1
YGL078C	DBP3
YGL079W	
YGL081W	
YGL082W	
YGL083W	SCY1
YGL084C	GUP1
YGL085W	
YGL086W	MAD1
YGL087C	MMS2
YGL089C	MF(ALPHA)2
YGL090W	LIF1
YGL094C	PAN2
YGL095C	VPS45
YGL096W	TOS8
YGL101W	
YGL104C	VPS73
YGL107C	RMD9
YGL108C	
YGL109W	
YGL110C	CUE3
YGL114W	
YGL115W	SNF4
YGL117W	
YGL118C	
YGL121C	GPG1
YGL125W	MET13
YGL126W	SCS3
YGL131C	SNT2
YGL132W	
YGL133W	ITC1
YGL138C	
YGL139W	FLC3
YGL140C	
YGL141W	HUL5
YGL144C	ROG1
YGL146C	
YGL148W	ARO2
YGL149W	
YGL151W	NUT1
YGL154C	LYS5
YGL156W	AMS1
YGL157W	
YGL158W	RCK1
YGL159W	
YGL161C	YIP5
YGL162W	SUT1
YGL164C	YRB30
YGL165C	
YGL166W	CUP2
YGL167C	PMR1
YGL170C	SPO74

YGL175C	SAE2
YGL176C	
YGL177W	
YGL179C	TOS3
YGL180W	ATG1
YGL181W	GTS1
YGL195W	GCN1
YGL196W	
YGL197W	MDS3
YGL198W	YIP4
YGL199C	
YGL200C	EMP24
YGL202W	ARO8
YGL203C	KEX1
YGL205W	POX1
YGL208W	SIP2
YGL209W	MIG2
YGL210W	YPT32
YGL211W	NCS6
YGL215W	CLG1
YGL216W	KIP3
YGL217C	
YGL218W	
YGL220W	
YGL221C	NIF3
YGL222C	EDC1
YGL224C	SDT1
YGL226W	
YGL227W	VID30
YGL229C	SAP4
YGL230C	
YGL231C	
YGL232W	TAN1
YGL235W	
YGL237C	HAP2
YGL240W	DOC1
YGL241W	KAP114
YGL242C	
YGL243W	TAD1
YGL248W	PDE1
YGL249W	ZIP2
YGL250W	
YGL252C	RTG2
YGL254W	FZF1
YGL255W	ZRT1
YGL257C	MNT2
YGL258W	VEL1
YGL259W	YPS5
YGL260W	
YGL261C	PAU11
YGL262W	
YGL263W	COS12
YGR001C	
YGR003W	CUL3
YGR006W	PRP18
YGR008C	STF2
YGR010W	NMA2
YGR011W	
YGR012W	
YGR014W	MSB2
YGR015C	
YGR016W	
YGR017W	
YGR018C	
YGR019W	UGA1
YGR021W	
YGR022C	
YGR023W	MTL1
YGR025W	
YGR026W	
YGR027C	RPS25A
YGR028W	MSP1
YGR031W	
YGR032W	GSC2
YGR033C	TIM21
YGR034W	RPL26B
YGR035C	
YGR036C	CAX4
YGR037C	ACB1
YGR038W	ORM1
YGR039W	
YGR040W	KSS1
YGR041W	BUD9
YGR042W	
YGR043C	
YGR044C	RME1
YGR045C	
YGR049W	SCM4
YGR050C	
YGR051C	
YGR052W	FMP48
YGR053C	
YGR054W	
YGR055W	MUP1
YGR058W	
YGR059W	SPR3

YGR062C	COX18
YGR066C	
YGR067C	
YGR068C	
YGR069W	
YGR070W	ROM1
YGR071C	
YGR072W	UPF3
YGR078C	PAC10
YGR079W	
YGR080W	TWF1
YGR084C	MRP13
YGR085C	RPL11B
YGR086C	PIL1
YGR087C	PDC6
YGR088W	CTT1
YGR089W	NNF2
YGR092W	DBF2
YGR093W	
YGR096W	TPC1
YGR097W	ASK10
YGR100W	MDR1
YGR104C	SRB5
YGR105W	VMA21
YGR107W	
YGR108W	CLB1
YGR109C	CLB6
YGR110W	
YGR111W	
YGR112W	SHY1
YGR117C	
YGR121C	MEP1
YGR122C-A	
YGR123C	PPT1
YGR124W	ASN2
YGR125W	
YGR126W	
YGR127W	
YGR129W	SYF2
YGR130C	
YGR131W	
YGR132C	PHB1
YGR134W	CAF130
YGR136W	LSB1
YGR137W	
YGR138C	TPO2
YGR139W	
YGR141W	VPS62
YGR142W	BTN2
YGR143W	SKN1
YGR144W	THI4
YGR146C	
YGR148C	RPL24B
YGR149W	
YGR151C	
YGR152C	RSR1
YGR153W	
YGR154C	GTO1
YGR155W	CYS4
YGR161C	RTS3
YGR164W	
YGR166W	KRE11
YGR167W	CLC1
YGR168C	
YGR169C	PUS6
YGR171C	MSM1
YGR173W	RBG2
YGR174C	CBP4
YGR176W	
YGR177C	ATF2
YGR178C	PBP1
YGR180C	RNR4
YGR181W	TIM13
YGR183C	QCR9
YGR188C	BUB1
YGR189C	CRH1
YGR193C	PDX1
YGR194C	XKS1
YGR196C	FYV8
YGR199W	PMT6
YGR201C	
YGR202C	PCT1
YGR203W	
YGR205W	
YGR207C	
YGR208W	SER2
YGR209C	TRX2
YGR210C	
YGR212W	SLI1
YGR213C	RTA1
YGR221C	TOS2
YGR222W	PET54
YGR223C	HSV2
YGR224W	AZR1
YGR225W	AMA1
YGR226C	

YGR228W	
YGR229C	SMI1
YGR230W	BNS1
YGR231C	PHB2
YGR232W	NAS6
YGR233C	PHO81
YGR234W	YHB1
YGR235C	
YGR236C	SPG1
YGR237C	
YGR238C	KEL2
YGR239C	PEX21
YGR241C	YAP1802
YGR242W	
YGR243W	FMP43
YGR244C	LSC2
YGR247W	CPD1
YGR248W	SOL4
YGR249W	MGA1
YGR252W	GCN5
YGR254W	ENO1
YGR256W	GND2
YGR258C	RAD2
YGR262C	BUD32
YGR263C	SAY1
YGR266W	
YGR268C	HUA1
YGR269W	
YGR271W	SLH1
YGR273C	
YGR275W	RTT102
YGR279C	SCW4
YGR281W	YOR1
YGR282C	BGL2
YGR283C	
YGR286C	BIO2
YGR287C	
YGR288W	MAL13
YGR290W	
YGR291C	
YGR292W	MAL12
YGR295C	COS6
YHL003C	LAG1
YHL005C	
YHL006C	SHU1
YHL007C	STE20
YHL008C	
YHL010C	
YHL012W	
YHL013C	OTU2
YHL014C	YLF2
YHL016C	DUR3
YHL017W	
YHL019C	APM2
YHL021C	FMP12
YHL022C	SPO11
YHL024W	RIM4
YHL026C	
YHL028W	WSC4
YHL030W	ECM29
YHL032C	GUT1
YHL033C	RPL8A
YHL034C	SBP1
YHL035C	VMR1
YHL036W	MUP3
YHL037C	
YHL038C	CBP2
YHL039W	
YHL041W	
YHL042W	
YHL043W	ECM34
YHL044W	
YHL045W	
YHL046C	PAU13
YHL047C	ARN2
YHR001W-A	QCR10
YHR003C	
YHR006W	STP2
YHR008C	SOD2
YHR009C	
YHR014W	SPO13
YHR015W	MIP6
YHR016C	YSC84
YHR017W	YSC83
YHR021C	RPS27B
YHR021W-A	ECM12
YHR022C	
YHR026W	PPA1
YHR028C	DAP2
YHR029C	YHI9
YHR032W	
YHR033W	
YHR035W	
YHR037W	PUT2
YHR039C	MSC7
YHR039C-B	

YHR043C	DOG2
YHR044C	DOG1
YHR046C	INM1
YHR047C	AAP1
YHR048W	
YHR049C-A	
YHR049W	FSH1
YHR050W	SMF2
YHR057C	CPR2
YHR060W	VMA22
YHR061C	GIC1
YHR075C	PPE1
YHR076W	PTC7
YHR077C	NMD2
YHR079C-B	
YHR080C	
YHR082C	KSP1
YHR086W	NAM8
YHR087W	
YHR091C	MSR1
YHR092C	HXT4
YHR093W	AHT1
YHR094C	HXT1
YHR095W	
YHR096C	HXT5
YHR097C	
YHR104W	GRE3
YHR105W	YPT35
YHR106W	TRR2
YHR108W	GGA2
YHR109W	CTM1
YHR110W	ERP5
YHR111W	UBA4
YHR112C	
YHR113W	
YHR114W	BZZ1
YHR115C	DMA1
YHR117W	TOM71
YHR121W	LSM12
YHR123W	EPT1
YHR124W	NDT80
YHR125W	
YHR126C	
YHR127W	
YHR130C	
YHR131C	
YHR132C	ECM14
YHR132W-A	IGO2
YHR133C	NSG1
YHR134W	WSS1
YHR136C	SPL2
YHR137W	ARO9
YHR138C	
YHR139C	SPS100
YHR139C-A	
YHR140W	
YHR143W	DSE2
YHR146W	CRP1
YHR150W	PEX28
YHR151C	
YHR152W	SPO12
YHR153C	SPO16
YHR154W	RTT107
YHR155W	YSP1
YHR156C	LIN1
YHR157W	REC104
YHR158C	KEL1
YHR159W	
YHR160C	PEX18
YHR161C	YAP1801
YHR163W	SOL3
YHR167W	THP2
YHR168W	MTG2
YHR171W	ATG7
YHR176W	FMO1
YHR177W	
YHR178W	STB5
YHR179W	OYE2
YHR180W	
YHR182W	
YHR183W	GND1
YHR184W	SSP1
YHR185C	PFS1
YHR189W	PTH1
YHR191C	CTF8
YHR198C	FMP22
YHR199C	FMP34
YHR200W	RPN10
YHR202W	
YHR207C	SET5
YHR209W	CRG1
YHR210C	
YIL001W	
YIL002C	INP51
YIL007C	NAS2
YIL008W	URM1

YIL009C-A	EST3
YIL009W	FAA3
YIL010W	DOT5
YIL011W	TIR3
YIL012W	
YIL013C	PDR11
YIL014W	MNT3
YIL015W	BAR1
YIL020C	HIS6
YIL023C	YKE4
YIL024C	
YIL025C	
YIL029C	
YIL030C	SSM4
YIL032C	
YIL034C	CAP2
YIL035C	CKA1
YIL037C	PRM2
YIL040W	APQ12
YIL042C	PKP1
YIL043C	CBR1
YIL045W	PIG2
YIL049W	DFG10
YIL054W	
YIL055C	
YIL056W	VHR1
YIL057C	
YIL058W	
YIL059C	
YIL064W	
YIL066C	RNR3
YIL067C	
YIL070C	MAM33
YIL071C	PCI8
YIL072W	HOP1
YIL073C	SPO22
YIL074C	SER33
YIL079C	AIR1
YIL084C	SDS3
YIL085C	KTR7
YIL086C	
YIL087C	
YIL088C	AVT7
YIL089W	
YIL092W	
YIL094C	LYS12
YIL096C	
YIL099W	SGA1
YIL100W	
YIL101C	XBP1
YIL102C	
YIL105C	SLM1
YIL107C	PFK26
YIL108W	
YIL111W	COX5B
YIL113W	SDP1
YIL114C	POR2
YIL116W	HIS5
YIL117C	PRM5
YIL120W	QDR1
YIL121W	QDR2
YIL122W	POG1
YIL123W	SIM1
YIL124W	AYR1
YIL128W	MET18
YIL130W	ASG1
YIL131C	FKH1
YIL132C	CSM2
YIL134W	FLX1
YIL136W	OM45
YIL138C	TPM2
YIL139C	REV7
YIL140W	AXL2
YIL141W	
YIL145C	PAN6
YIL146C	ECM37
YIL149C	MLP2
YIL152W	
YIL153W	RRD1
YIL154C	IMP2'
YIL155C	GUT2
YIL156W	UBP7
YIL157C	FMP35
YIL158W	
YIL159W	BNR1
YIL161W	
YIL163C	
YIL164C	NIT1
YIL165C	
YIL166C	
YIL167W	
YIL168W	
YIL170W	
YIL173W	VTH1
YIR001C	SGN1
YIR002C	MPH1

YIR003W	
YIR004W	DJP1
YIR007W	
YIR009W	MSL1
YIR013C	GAT4
YIR014W	
YIR016W	
YIR017C	MET28
YIR018W	YAP5
YIR019C	MUC1
YIR020C	
YIR020W-B	
YIR021W	MRS1
YIR023W	DAL81
YIR024C	
YIR025W	MND2
YIR027C	DAL1
YIR028W	DAL4
YIR029W	DAL2
YIR030C	DCG1
YIR031C	DAL7
YIR032C	DAL3
YIR035C	
YIR036C	IRC24
YIR038C	GTT1
YIR039C	YPS6
YIR042C	
YIR043C	
YIR044C	
YJL003W	COX16
YJL004C	SYS1
YJL006C	CTK2
YJL007C	
YJL013C	MAD3
YJL016W	
YJL017W	
YJL020C	BBC1
YJL021C	
YJL022W	
YJL027C	
YJL028W	
YJL030W	MAD2
YJL037W	IRC18
YJL038C	
YJL042W	MHP1
YJL043W	
YJL044C	GYP6
YJL045W	
YJL047C	RTT101
YJL048C	UBX6
YJL049W	
YJL052W	TDH1
YJL055W	
YJL056C	ZAP1
YJL057C	IKS1
YJL058C	BIT61
YJL059W	YHC3
YJL060W	BNA3
YJL064W	
YJL065C	DLS1
YJL066C	MPM1
YJL067W	
YJL068C	
YJL070C	
YJL071W	ARG2
YJL077C	ICS3
YJL078C	PRY3
YJL079C	PRY1
YJL082W	IML2
YJL083W	TAX4
YJL084C	ALY2
YJL089W	SIP4
YJL092W	HPR5
YJL093C	TOK1
YJL094C	KHA1
YJL095W	BCK1
YJL096W	MRPL49
YJL099W	CHS6
YJL100W	LSB6
YJL101C	GSH1
YJL103C	GSM1
YJL105W	SET4
YJL106W	IME2
YJL107C	
YJL108C	PRM10
YJL110C	GZF3
YJL112W	MDV1
YJL116C	NCA3
YJL118W	
YJL119C	
YJL122W	ALB1
YJL126W	NIT2
YJL129C	TRK1
YJL130C	URA2
YJL131C	
YJL132W	

YJL137C	GLG2
YJL139C	YUR1
YJL140W	RPB4
YJL142C	IRC9
YJL144W	
YJL145W	SFH5
YJL147C	
YJL148W	RPA34
YJL151C	SNA3
YJL153C	INO1
YJL155C	FBP26
YJL157C	FAR1
YJL158C	CIS3
YJL160C	
YJL161W	FMP33
YJL162C	JJJ2
YJL163C	
YJL165C	HAL5
YJL166W	QCR8
YJL169W	
YJL170C	ASG7
YJL171C	
YJL172W	CPS1
YJL176C	SWI3
YJL177W	RPL17B
YJL180C	ATP12
YJL181W	
YJL182C	
YJL183W	MNN11
YJL185C	
YJL186W	MNN5
YJL187C	SWE1
YJL188C	BUD19
YJL189W	RPL39
YJL190C	RPS22A
YJL191W	RPS14B
YJL196C	ELO1
YJL197W	UBP12
YJL198W	PHO90
YJL199C	MBB1
YJL200C	ACO2
YJL206C	
YJL206C-A	
YJL207C	LAA1
YJL209W	CBP1
YJL210W	PEX2
YJL212C	OPT1
YJL213W	
YJL214W	HXT8
YJL215C	
YJL216C	
YJL217W	
YJL218W	
YJR001W	AVT1
YJR003C	
YJR004C	SAG1
YJR008W	
YJR009C	TDH2
YJR010C-A	SPC1
YJR010W	MET3
YJR011C	
YJR014W	TMA22
YJR015W	
YJR018W	
YJR019C	TES1
YJR020W	
YJR021C	REC107
YJR024C	
YJR025C	BNA1
YJR026W	
YJR030C	
YJR031C	GEA1
YJR035W	RAD26
YJR036C	HUL4
YJR037W	
YJR038C	
YJR039W	
YJR043C	POL32
YJR047C	ANB1
YJR048W	CYC1
YJR050W	ISY1
YJR051W	OSM1
YJR052W	RAD7
YJR053W	BFA1
YJR056C	
YJR058C	APS2
YJR060W	CBF1
YJR061W	
YJR062C	NTA1
YJR063W	RPA12
YJR069C	HAM1
YJR070C	LIA1
YJR074W	MOG1
YJR077C	MIR1
YJR078W	BNA2
YJR079W	

YJR080C	FMP26
YJR084W	CSN12
YJR087W	
YJR088C	
YJR091C	JSN1
YJR092W	BUD4
YJR094C	IME1
YJR094W-A	RPL43B
YJR095W	SFC1
YJR096W	
YJR097W	JJJ3
YJR098C	
YJR099W	YUH1
YJR100C	
YJR102C	VPS25
YJR103W	URA8
YJR105W	ADO1
YJR106W	ECM27
YJR107W	
YJR108W	ABM1
YJR109C	CPA2
YJR110W	YMR1
YJR111C	
YJR113C	RSM7
YJR115W	
YJR116W	
YJR119C	JHD2
YJR120W	
YJR121W	ATP2
YJR124C	
YJR125C	ENT3
YJR126C	VPS70
YJR127C	RSF2
YJR128W	
YJR129C	
YJR130C	STR2
YJR131W	MNS1
YJR133W	XPT1
YJR134C	SGM1
YJR135C	MCM22
YJR137C	ECM17
YJR140C	HIR3
YJR144W	MGM101
YJR146W	
YJR147W	HMS2
YJR149W	
YJR150C	DAN1
YJR152W	DAL5
YJR153W	PGU1
YJR154W	
YKL001C	MET14
YKL002W	DID4
YKL005C	BYE1
YKL006W	RPL14A
YKL008C	LAC1
YKL010C	UFD4
YKL011C	CCE1
YKL015W	PUT3
YKL017C	HCS1
YKL020C	SPT23
YKL023W	
YKL025C	PAN3
YKL026C	GPX1
YKL027W	
YKL029C	MAE1
YKL030W	
YKL031W	
YKL033W-A	
YKL038W	RGT1
YKL039W	PTM1
YKL043W	PHD1
YKL044W	
YKL046C	DCW1
YKL050C	
YKL051W	SFK1
YKL053C-A	MDM35
YKL053W	
YKL055C	OAR1
YKL056C	TMA19
YKL057C	NUP120
YKL061W	
YKL062W	MSN4
YKL064W	MNR2
YKL065C	YET1
YKL066W	
YKL067W	YNK1
YKL068W	NUP100
YKL069W	
YKL070W	
YKL071W	
YKL072W	STB6
YKL073W	LHS1
YKL075C	
YKL076C	PSY1
YKL079W	SMY1
YKL080W	VMA5

YKL081W	TEF4
YKL084W	HOT13
YKL085W	MDH1
YKL086W	SRX1
YKL087C	CYT2
YKL090W	CUE2
YKL091C	
YKL092C	BUD2
YKL093W	MBR1
YKL094W	YJU3
YKL096W	CWP1
YKL096W-A	CWP2
YKL097C	
YKL098W	
YKL100C	
YKL101W	HSL1
YKL102C	
YKL103C	LAP4
YKL105C	
YKL106W	AAT1
YKL107W	
YKL109W	HAP4
YKL113C	RAD27
YKL115C	
YKL116C	PRR1
YKL117W	SBA1
YKL118W	
YKL119C	VPH2
YKL120W	OAC1
YKL121W	
YKL123W	
YKL124W	SSH4
YKL126W	YPK1
YKL127W	PGM1
YKL128C	PMU1
YKL129C	MYO3
YKL130C	SHE2
YKL131W	
YKL132C	RMA1
YKL133C	
YKL135C	APL2
YKL136W	
YKL137W	
YKL139W	CTK1
YKL140W	TGL1
YKL142W	MRP8
YKL143W	LTV1
YKL146W	AVT3
YKL147C	
YKL148C	SDH1
YKL150W	MCR1
YKL151C	
YKL155C	RSM22
YKL156W	RPS27A
YKL157W	APE2
YKL158W	
YKL159C	RCN1
YKL161C	
YKL162C	
YKL162C-A	
YKL163W	PIR3
YKL164C	PIR1
YKL166C	TPK3
YKL167C	MRP49
YKL168C	KKQ8
YKL171W	
YKL174C	TPO5
YKL175W	ZRT3
YKL176C	LST4
YKL179C	COY1
YKL183W	LOT5
YKL184W	SPE1
YKL185W	ASH1
YKL187C	
YKL188C	PXA2
YKL191W	DPH2
YKL198C	PTK1
YKL199C	
YKL200C	
YKL201C	MNN4
YKL202W	
YKL205W	LOS1
YKL207W	
YKL208W	CBT1
YKL212W	SAC1
YKL213C	DOA1
YKL214C	YRA2
YKL215C	
YKL216W	URA1
YKL217W	JEN1
YKL218C	SRY1
YKL220C	FRE2
YKL221W	MCH2
YKL222C	
YKR003W	OSH6
YKR005C	

YKR006C	MRPL13
YKR009C	FOX2
YKR010C	TOF2
YKR011C	
YKR012C	
YKR013W	PRY2
YKR014C	YPT52
YKR015C	
YKR016W	FMP13
YKR017C	
YKR018C	
YKR019C	IRS4
YKR021W	ALY1
YKR027W	BCH2
YKR028W	SAP190
YKR030W	GMH1
YKR031C	SPO14
YKR033C	
YKR034W	DAL80
YKR035C	OPI8
YKR036C	CAF4
YKR039W	GAP1
YKR043C	
YKR044W	UIP5
YKR045C	
YKR046C	PET10
YKR047W	
YKR048C	NAP1
YKR049C	FMP46
YKR050W	TRK2
YKR053C	YSR3
YKR054C	DYN1
YKR055W	RHO4
YKR056W	TRM2
YKR058W	GLG1
YKR059W	TIF1
YKR060W	UTP30
YKR061W	KTR2
YKR064W	OAF3
YKR065C	PAM17
YKR066C	CCP1
YKR067W	GPT2
YKR069W	MET1
YKR070W	
YKR072C	SIS2
YKR073C	
YKR075C	
YKR076W	ECM4
YKR077W	
YKR078W	
YKR080W	MTD1
YKR082W	NUP133
YKR087C	OMA1
YKR088C	TVP38
YKR089C	TGL4
YKR090W	PXL1
YKR091W	SRL3
YKR092C	SRP40
YKR093W	PTR2
YKR094C	RPL40B
YKR095W	MLP1
YKR096W	
YKR097W	PCK1
YKR098C	UBP11
YKR099W	BAS1
YKR100C	SKG1
YKR101W	SIR1
YKR102W	FLO10
YKR103W	NFT1
YKR104W	
YKR105C	
YKR106W	
YLL001W	DNM1
YLL002W	RTT109
YLL005C	SPO75
YLL009C	COX17
YLL010C	PSR1
YLL012W	YEH1
YLL014W	
YLL015W	BPT1
YLL016W	
YLL017W	
YLL018C-A	COX19
YLL019C	KNS1
YLL020C	
YLL021W	SPA2
YLL023C	
YLL025W	PAU17
YLL026W	HSP104
YLL028W	TPO1
YLL029W	
YLL030C	
YLL032C	
YLL040C	VPS13
YLL041C	SDH2
YLL042C	ATG10

YLL043W	FPS1
YLL044W	
YLL045C	RPL8B
YLL046C	RNP1
YLL047W	
YLL048C	YBT1
YLL051C	FRE6
YLL052C	AQY2
YLL053C	
YLL054C	
YLL055W	
YLL056C	
YLL057C	JLP1
YLL058W	
YLL059C	
YLL060C	GTT2
YLL061W	MMP1
YLL062C	MHT1
YLL063C	AYT1
YLR001C	
YLR003C	
YLR004C	THI73
YLR011W	LOT6
YLR012C	
YLR013W	GAT3
YLR015W	BRE2
YLR016C	PML1
YLR017W	MEU1
YLR018C	POM34
YLR019W	PSR2
YLR020C	YEH2
YLR023C	IZH3
YLR025W	SNF7
YLR028C	ADE16
YLR030W	
YLR031W	
YLR032W	RAD5
YLR034C	SMF3
YLR035C	MLH2
YLR036C	
YLR037C	DAN2
YLR038C	COX12
YLR039C	RIC1
YLR040C	
YLR041W	
YLR042C	
YLR043C	TRX1
YLR044C	PDC1
YLR046C	
YLR047C	FRE8
YLR049C	
YLR050C	
YLR053C	
YLR054C	OSW2
YLR056W	ERG3
YLR057W	
YLR059C	REX2
YLR063W	
YLR064W	
YLR065C	
YLR067C	PET309
YLR070C	XYL2
YLR072W	
YLR073C	
YLR077W	FMP25
YLR080W	EMP46
YLR081W	GAL2
YLR082C	SRL2
YLR083C	EMP70
YLR084C	RAX2
YLR085C	ARP6
YLR087C	CSF1
YLR089C	ALT1
YLR090W	XDJ1
YLR092W	SUL2
YLR093C	NYV1
YLR094C	GIS3
YLR095C	IOC2
YLR096W	KIN2
YLR097C	HRT3
YLR098C	CHA4
YLR102C	APC9
YLR104W	
YLR107W	REX3
YLR108C	
YLR109W	AHP1
YLR110C	CCW12
YLR112W	
YLR113W	HOG1
YLR114C	AVL9
YLR118C	
YLR119W	SRN2
YLR120C	YPS1
YLR121C	YPS3
YLR122C	
YLR123C	

YLR124W	
YLR125W	
YLR126C	
YLR128W	DCN1
YLR130C	ZRT2
YLR133W	CKI1
YLR134W	PDC5
YLR135W	SLX4
YLR136C	TIS11
YLR137W	
YLR138W	NHA1
YLR142W	PUT1
YLR143W	
YLR146C	SPE4
YLR149C	
YLR150W	STM1
YLR151C	PCD1
YLR152C	
YLR154C	RNH203
YLR164W	
YLR165C	PUS5
YLR168C	
YLR169W	
YLR170C	APS1
YLR171W	
YLR172C	DPH5
YLR173W	
YLR174W	IDP2
YLR178C	TFS1
YLR179C	
YLR181C	VTA1
YLR183C	TOS4
YLR184W	
YLR187W	SKG3
YLR188W	MDL1
YLR189C	ATG26
YLR190W	MMR1
YLR193C	UPS1
YLR194C	
YLR199C	
YLR203C	MSS51
YLR204W	QRI5
YLR205C	HMX1
YLR206W	ENT2
YLR209C	PNP1
YLR210W	CLB4
YLR211C	
YLR213C	CRR1
YLR216C	CPR6
YLR217W	
YLR218C	
YLR219W	MSC3
YLR220W	CCC1
YLR221C	RSA3
YLR224W	
YLR227C	ADY4
YLR228C	ECM22
YLR231C	BNA5
YLR232W	
YLR233C	EST1
YLR235C	
YLR236C	
YLR238W	FAR10
YLR240W	VPS34
YLR241W	
YLR242C	ARV1
YLR244C	MAP1
YLR246W	ERF2
YLR247C	IRC20
YLR248W	RCK2
YLR250W	SSP120
YLR251W	SYM1
YLR252W	
YLR253W	
YLR254C	NDL1
YLR255C	
YLR257W	
YLR258W	GSY2
YLR260W	LCB5
YLR261C	VPS63
YLR262C-A	TMA7
YLR263W	RED1
YLR265C	NEJ1
YLR266C	PDR8
YLR267W	BOP2
YLR268W	SEC22
YLR270W	DCS1
YLR271W	
YLR273C	PIG1
YLR278C	
YLR279W	
YLR280C	
YLR281C	
YLR282C	
YLR283W	
YLR284C	ECI1

YLR285W	NNT1
YLR286C	CTS1
YLR287C	
YLR288C	MEC3
YLR290C	
YLR292C	SEC72
YLR294C	
YLR295C	ATP14
YLR296W	
YLR297W	
YLR299W	ECM38
YLR300W	EXG1
YLR304C	ACO1
YLR306W	UBC12
YLR307W	CDA1
YLR308W	CDA2
YLR309C	IMH1
YLR311C	
YLR312C	
YLR312W-A	MRPL15
YLR313C	SPH1
YLR318W	EST2
YLR319C	BUD6
YLR320W	MMS22
YLR324W	PEX30
YLR325C	RPL38
YLR326W	
YLR327C	TMA10
YLR328W	NMA1
YLR329W	REC102
YLR330W	CHS5
YLR331C	JIP3
YLR332W	MID2
YLR333C	RPS25B
YLR334C	
YLR337C	VRP1
YLR338W	OPI9
YLR341W	SPO77
YLR343W	GAS2
YLR344W	RPL26A
YLR345W	
YLR346C	
YLR348C	DIC1
YLR349W	
YLR350W	ORM2
YLR351C	NIT3
YLR352W	
YLR353W	BUD8
YLR354C	TAL1
YLR356W	
YLR358C	
YLR361C	DCR2
YLR363C	NMD4
YLR364W	
YLR365W	
YLR366W	
YLR367W	RPS22B
YLR370C	ARC18
YLR371W	ROM2
YLR372W	SUR4
YLR374C	
YLR375W	STP3
YLR376C	PSY3
YLR377C	FBP1
YLR380W	CSR1
YLR381W	CTF3
YLR385C	SWC7
YLR387C	REH1
YLR389C	STE23
YLR390W	ECM19
YLR390W-A	CCW14
YLR392C	
YLR393W	ATP10
YLR394W	CST9
YLR395C	COX8
YLR396C	VPS33
YLR398C	SKI2
YLR399C	BDF1
YLR400W	
YLR401C	DUS3
YLR402W	
YLR405W	DUS4
YLR407W	
YLR408C	
YLR413W	
YLR414C	
YLR415C	
YLR416C	
YLR417W	VPS36
YLR421C	RPN13
YLR422W	
YLR426W	
YLR427W	MAG2
YLR428C	
YLR429W	CRN1
YLR431C	ATG23

YLR432W	IMD3
YLR435W	TSR2
YLR437C	
YLR438W	CAR2
YLR441C	RPS1A
YLR443W	ECM7
YLR444C	
YLR445W	
YLR446W	
YLR447C	VMA6
YLR449W	FPR4
YLR450W	HMG2
YLR451W	LEU3
YLR452C	SST2
YLR453C	RIF2
YLR454W	FMP27
YLR456W	
YLR460C	
YLR461W	PAU4
YML001W	YPT7
YML002W	
YML003W	
YML004C	GLO1
YML005W	TRM12
YML009C	MRPL39
YML011C	RAD33
YML013C-A	
YML016C	PPZ1
YML018C	
YML019W	OST6
YML020W	
YML021C	UNG1
YML022W	APT1
YML027W	YOX1
YML029W	USA1
YML030W	
YML033W	
YML034W	SRC1
YML036W	CGI121
YML037C	
YML038C	YMD8
YML041C	VPS71
YML042W	CAT2
YML047C	PRM6
YML048W	GSF2
YML048W-A	
YML050W	
YML051W	GAL80
YML052W	SUR7
YML053C	
YML054C	CYB2
YML056C	IMD4
YML057W	CMP2
YML058C-A	
YML058W	SML1
YML059C	NTE1
YML060W	OGG1
YML062C	MFT1
YML063W	RPS1B
YML066C	SMA2
YML067C	ERV41
YML068W	ITT1
YML070W	DAK1
YML072C	TCB3
YML074C	FPR3
YML075C	HMG1
YML076C	WAR1
YML078W	CPR3
YML080W	DUS1
YML081C-A	ATP18
YML081W	
YML082W	
YML083C	
YML084W	
YML086C	ALO1
YML087C	
YML088W	UFO1
YML089C	
YML090W	
YML094W	GIM5
YML096W	
YML099C	ARG81
YML100W	TSL1
YML100W-A	
YML101C	CUE4
YML102C-A	
YML102W	CAC2
YML104C	MDM1
YML106W	URA5
YML107C	PML39
YML108W	
YML109W	ZDS2
YML110C	COQ5
YML111W	BUL2
YML112W	CTK3
YML113W	DAT1

YML116W	ATR1
YML117W-A	
YML118W	NGL3
YML119W	
YML120C	NDI1
YML123C	PHO84
YML124C	TUB3
YML129C	COX14
YML131W	
YMR002W	MIC17
YMR003W	
YMR004W	MVP1
YMR006C	PLB2
YMR007W	
YMR008C	PLB1
YMR009W	ADI1
YMR011W	HXT2
YMR012W	CLU1
YMR015C	ERG5
YMR017W	SPO20
YMR018W	
YMR019W	STB4
YMR020W	FMS1
YMR021C	MAC1
YMR022W	QRI8
YMR023C	MSS1
YMR024W	MRPL3
YMR025W	CSI1
YMR026C	PEX12
YMR027W	
YMR029C	FAR8
YMR030W	RSF1
YMR031C	
YMR031W-A	
YMR032W	HOF1
YMR034C	
YMR035W	IMP2
YMR037C	MSN2
YMR039C	SUB1
YMR040W	YET2
YMR041C	
YMR042W	ARG80
YMR044W	IOC4
YMR048W	CSM3
YMR052C-A	
YMR052W	FAR3
YMR053C	STB2
YMR055C	BUB2
YMR056C	AAC1
YMR057C	
YMR060C	SAM37
YMR065W	KAR5
YMR067C	UBX4
YMR068W	AVO2
YMR069W	NAT4
YMR071C	TVP18
YMR074C	
YMR077C	VPS20
YMR078C	CTF18
YMR080C	NAM7
YMR081C	ISF1
YMR082C	
YMR083W	ADH3
YMR084W	
YMR085W	
YMR086C-A	
YMR086W	
YMR087W	
YMR088C	VBA1
YMR090W	
YMR092C	AIP1
YMR095C	SNO1
YMR096W	SNZ1
YMR101C	SRT1
YMR102C	
YMR103C	
YMR104C	YPK2
YMR105C	PGM2
YMR107W	SPG4
YMR109W	MYO5
YMR110C	HFD1
YMR111C	
YMR114C	
YMR115W	FMP24
YMR118C	
YMR119W-A	
YMR120C	ADE17
YMR121C	RPL15B
YMR122C	
YMR124W	
YMR125W	STO1
YMR126C	DLT1
YMR127C	SAS2
YMR129W	POM152
YMR130W	
YMR132C	JLP2

YMR133W	REC114
YMR135C	GID8
YMR135W-A	
YMR136W	GAT2
YMR137C	PSO2
YMR138W	CIN4
YMR139W	RIM11
YMR140W	SIP5
YMR141C	
YMR143W	RPS16A
YMR144W	
YMR145C	NDE1
YMR147W	
YMR148W	
YMR150C	IMP1
YMR151W	YIM2
YMR153C-A	
YMR153W	NUP53
YMR155W	
YMR156C	TPP1
YMR157C	FMP39
YMR158C-B	
YMR158W	MRPS8
YMR158W-A	
YMR160W	
YMR161W	HLJ1
YMR163C	INP2
YMR164C	MSS11
YMR166C	
YMR169C	ALD3
YMR170C	ALD2
YMR171C	
YMR172C-A	
YMR172W	HOT1
YMR173W	DDR48
YMR173W-A	
YMR174C	PAI3
YMR175W	SIP18
YMR176W	ECM5
YMR177W	MMT1
YMR178W	
YMR180C	CTL1
YMR181C	
YMR182C	RGM1
YMR183C	SSO2
YMR184W	ADD37
YMR186W	HSC82
YMR187C	
YMR189W	GCV2
YMR191W	SPG5
YMR192W	GYL1
YMR193C-A	
YMR194C-A	
YMR194W	RPL36A
YMR195W	ICY1
YMR196W	
YMR198W	CIK1
YMR201C	RAD14
YMR202W	ERG2
YMR204C	INP1
YMR206W	
YMR207C	HFA1
YMR209C	
YMR210W	
YMR215W	GAS3
YMR219W	ESC1
YMR221C	FMP42
YMR222C	FSH2
YMR224C	MRE11
YMR225C	MRPL44
YMR226C	TMA29
YMR232W	FUS2
YMR233W	
YMR234W	RNH1
YMR237W	BCH1
YMR242C	RPL20A
YMR243C	ZRC1
YMR244C-A	
YMR244W	
YMR245W	
YMR246W	FAA4
YMR247C	RKR1
YMR250W	GAD1
YMR251W	GTO3
YMR251W-A	HOR7
YMR252C	
YMR253C	
YMR254C	
YMR255W	GFD1
YMR256C	COX7
YMR257C	PET111
YMR259C	
YMR261C	TPS3
YMR262W	
YMR264W	CUE1
YMR265C	

YMR266W	RSN1
YMR269W	TMA23
YMR271C	URA10
YMR272C	SCS7
YMR273C	ZDS1
YMR274C	RCE1
YMR278W	
YMR279C	
YMR280C	CAT8
YMR283C	RIT1
YMR285C	NGL2
YMR286W	MRPL33
YMR289W	ABZ2
YMR291W	
YMR292W	GOT1
YMR294W-A	
YMR295C	
YMR297W	PRC1
YMR302C	YME2
YMR303C	ADH2
YMR304C-A	
YMR305C	SCW10
YMR306C-A	
YMR306W	FKS3
YMR307W	GAS1
YMR310C	
YMR311C	GLC8
YMR315W	
YMR316C-A	
YMR316C-B	
YMR316W	DIA1
YMR317W	
YMR318C	ADH6
YMR319C	FET4
YMR320W	
YMR322C	SNO4
YMR326C	
YNL001W	DOM34
YNL004W	HRB1
YNL009W	IDP3
YNL010W	
YNL011C	
YNL012W	SPO1
YNL013C	
YNL014W	HEF3
YNL020C	ARK1
YNL022C	
YNL024C	
YNL031C	HHT2
YNL032W	SIW14
YNL034W	
YNL035C	
YNL043C	
YNL044W	YIP3
YNL045W	
YNL046W	
YNL047C	SLM2
YNL049C	SFB2
YNL051W	COG5
YNL052W	COX5A
YNL053W	MSG5
YNL056W	OCA2
YNL057W	
YNL058C	
YNL059C	ARP5
YNL063W	MTQ1
YNL065W	AQR1
YNL066W	SUN4
YNL068C	FKH2
YNL070W	TOM7
YNL072W	RNH201
YNL077W	APJ1
YNL078W	NIS1
YNL080C	EOS1
YNL082W	PMS1
YNL083W	SAL1
YNL084C	END3
YNL085W	MKT1
YNL086W	
YNL087W	TCB2
YNL089C	
YNL090W	RHO2
YNL092W	
YNL093W	YPT53
YNL094W	APP1
YNL095C	
YNL098C	RAS2
YNL099C	OCA1
YNL100W	
YNL101W	AVT4
YNL104C	LEU4
YNL105W	
YNL106C	INP52
YNL107W	YAF9
YNL108C	
YNL109W	

YNL111C	CYB5
YNL115C	
YNL116W	DMA2
YNL117W	MLS1
YNL122C	
YNL123W	NMA111
YNL125C	ESBP6
YNL127W	FAR11
YNL128W	TEP1
YNL129W	NRK1
YNL130C	CPT1
YNL133C	FYV6
YNL134C	
YNL135C	FPR1
YNL136W	EAF7
YNL138W	SRV2
YNL139C	RLR1
YNL140C	
YNL142W	MEP2
YNL143C	
YNL144C	
YNL145W	MFA2
YNL146W	
YNL147W	LSM7
YNL154C	YCK2
YNL155W	
YNL157W	IGO1
YNL159C	ASI2
YNL160W	YGP1
YNL168C	FMP41
YNL173C	MDG1
YNL175C	NOP13
YNL176C	
YNL179C	
YNL187W	
YNL190W	
YNL191W	DUG3
YNL193W	
YNL194C	
YNL195C	
YNL196C	
YNL197C	WHI3
YNL200C	
YNL202W	SPS19
YNL203C	
YNL204C	SPS18
YNL205C	
YNL211C	
YNL212W	VID27
YNL214W	PEX17
YNL217W	
YNL220W	ADE12
YNL225C	CNM67
YNL230C	ELA1
YNL231C	PDR16
YNL233W	BNI4
YNL235C	
YNL237W	YTP1
YNL239W	LAP3
YNL242W	ATG2
YNL246W	VPS75
YNL249C	MPA43
YNL250W	RAD50
YNL253W	TEX1
YNL254C	
YNL255C	GIS2
YNL257C	SIP3
YNL259C	ATX1
YNL265C	IST1
YNL266W	
YNL268W	LYP1
YNL269W	BSC4
YNL270C	ALP1
YNL273W	TOF1
YNL274C	
YNL275W	BOR1
YNL278W	CAF120
YNL279W	PRM1
YNL280C	ERG24
YNL281W	HCH1
YNL285W	
YNL286W	CUS2
YNL289W	PCL1
YNL292W	PUS4
YNL293W	MSB3
YNL295W	
YNL296W	
YNL297C	MON2
YNL299W	TRF5
YNL300W	
YNL301C	RPL18B
YNL303W	
YNL304W	YPT11
YNL305C	
YNL307C	MCK1
YNL311C	SKP2

YNL315C	ATP11
YNL316C	PHA2
YNL318C	HXT14
YNL319W	
YNL321W	
YNL326C	PFA3
YNL327W	EGT2
YNL328C	MDJ2
YNL332W	THI12
YNL333W	SNZ2
YNL334C	SNO2
YNL335W	DDI3
YNL336W	COS1
YNL338W	
YNL339C	YRF1-6
YNR001C	CIT1
YNR002C	ATO2
YNR004W	
YNR005C	
YNR008W	LRO1
YNR009W	NRM1
YNR013C	PHO91
YNR014W	
YNR015W	SMM1
YNR018W	
YNR019W	ARE2
YNR024W	
YNR025C	
YNR027W	BUD17
YNR028W	CPR8
YNR031C	SSK2
YNR032C-A	HUB1
YNR032W	PPG1
YNR033W	ABZ1
YNR034W	SOL1
YNR037C	RSM19
YNR041C	COQ2
YNR042W	
YNR045W	PET494
YNR048W	
YNR049C	MSO1
YNR052C	POP2
YNR055C	HOL1
YNR056C	BIO5
YNR057C	BIO4
YNR058W	BIO3
YNR059W	MNT4
YNR060W	FRE4
YNR061C	
YNR062C	
YNR063W	
YNR064C	
YNR065C	YSN1
YNR066C	
YNR069C	BSC5
YNR070W	
YNR071C	
YNR072W	HXT17
YNR073C	
YNR075W	COS10
YOL002C	IZH2
YOL003C	PFA4
YOL006C	TOP1
YOL007C	CSI2
YOL008W	COQ10
YOL011W	PLB3
YOL013C	HRD1
YOL013W-A	
YOL014W	
YOL015W	IRC10
YOL016C	CMK2
YOL017W	ESC8
YOL019W	
YOL020W	TAT2
YOL024W	
YOL025W	LAG2
YOL027C	MDM38
YOL028C	YAP7
YOL029C	
YOL030W	GAS5
YOL031C	SIL1
YOL032W	OPI10
YOL037C	
YOL039W	RPP2A
YOL042W	NGL1
YOL043C	NTG2
YOL045W	PSK2
YOL046C	
YOL047C	
YOL048C	
YOL050C	
YOL051W	GAL11
YOL052C	SPE2
YOL053C-A	
YOL053W	
YOL054W	PSH1

YOL055C	THI20
YOL056W	GPM3
YOL057W	
YOL058W	ARG1
YOL059W	GPD2
YOL060C	MAM3
YOL061W	PRS5
YOL062C	APM4
YOL063C	CRT10
YOL065C	INP54
YOL067C	RTG1
YOL070C	NBA1
YOL071W	EMI5
YOL072W	THP1
YOL075C	
YOL076W	MDM20
YOL079W	
YOL080C	REX4
YOL082W	ATG19
YOL083W	
YOL084W	PHM7
YOL085C	
YOL088C	MPD2
YOL089C	HAL9
YOL090W	MSH2
YOL091W	SPO21
YOL092W	
YOL093W	TRM10
YOL096C	COQ3
YOL098C	
YOL099C	
YOL101C	IZH4
YOL103W	ITR2
YOL104C	NDJ1
YOL105C	WSC3
YOL106W	
YOL107W	
YOL109W	ZEO1
YOL111C	MDY2
YOL112W	MSB4
YOL113W	SKM1
YOL114C	
YOL117W	RRI2
YOL118C	
YOL119C	MCH4
YOL122C	SMF1
YOL126C	MDH2
YOL131W	
YOL132W	GAS4
YOL136C	PFK27
YOL137W	BSC6
YOL138C	
YOL141W	PPM2
YOL147C	PEX11
YOL148C	SPT20
YOL150C	
YOL151W	GRE2
YOL152W	FRE7
YOL153C	
YOL155C	HPF1
YOL158C	ENB1
YOL159C	
YOL160W	
YOL162W	
YOL163W	
YOR003W	YSP3
YOR005C	DNL4
YOR007C	SGT2
YOR008C	SLG1
YOR009W	TIR4
YOR010C	TIR2
YOR011W	AUS1
YOR012W	
YOR013W	IRC11
YOR014W	RTS1
YOR015W	
YOR018W	ROD1
YOR019W	
YOR021C	
YOR022C	
YOR023C	AHC1
YOR024W	IRC12
YOR025W	HST3
YOR027W	STI1
YOR028C	CIN5
YOR029W	
YOR031W	CRS5
YOR032C	HMS1
YOR033C	EXO1
YOR034C	AKR2
YOR036W	PEP12
YOR037W	CYC2
YOR038C	HIR2
YOR040W	GLO4
YOR041C	
YOR042W	CUE5

YOR044W	IRC23
YOR045W	TOM6
YOR047C	STD1
YOR049C	RSB1
YOR050C	
YOR051C	
YOR052C	
YOR053W	
YOR054C	VHS3
YOR055W	
YOR058C	ASE1
YOR059C	
YOR062C	
YOR064C	YNG1
YOR066W	
YOR070C	GYP1
YOR071C	THI71
YOR072W	
YOR079C	ATX2
YOR080W	DIA2
YOR081C	TGL5
YOR082C	
YOR083W	WHI5
YOR084W	
YOR086C	TCB1
YOR087W	YVC1
YOR088W	
YOR090C	PTC5
YOR091W	TMA46
YOR092W	ECM3
YOR093C	
YOR094W	ARF3
YOR097C	
YOR099W	KTR1
YOR100C	CRC1
YOR101W	RAS1
YOR105W	
YOR107W	RGS2
YOR108W	LEU9
YOR109W	INP53
YOR111W	
YOR113W	AZF1
YOR114W	
YOR115C	TRS33
YOR118W	
YOR120W	GCY1
YOR121C	
YOR126C	IAH1
YOR127W	RGA1
YOR129C	
YOR130C	ORT1
YOR131C	
YOR133W	EFT1
YOR134W	BAG7
YOR137C	SIA1
YOR138C	RUP1
YOR139C	
YOR141C	ARP8
YOR142W	LSC1
YOR144C	ELG1
YOR152C	
YOR153W	PDR5
YOR154W	SLP1
YOR155C	ISN1
YOR156C	NFI1
YOR158W	PET123
YOR161C	PNS1
YOR162C	YRR1
YOR163W	DDP1
YOR164C	
YOR165W	SEY1
YOR166C	SWT1
YOR170W	
YOR171C	LCB4
YOR172W	YRM1
YOR173W	DCS2
YOR175C	
YOR177C	MPC54
YOR178C	GAC1
YOR180C	DCI1
YOR185C	GSP2
YOR186W	
YOR188W	MSB1
YOR189W	IES4
YOR190W	SPR1
YOR191W	RIS1
YOR192C	THI72
YOR193W	PEX27
YOR195W	SLK19
YOR197W	MCA1
YOR199W	
YOR200W	
YOR202W	HIS3
YOR208W	PTP2
YOR213C	SAS5
YOR214C	

YOR215C	
YOR219C	STE13
YOR220W	
YOR222W	ODC2
YOR225W	
YOR226C	ISU2
YOR227W	
YOR228C	
YOR229W	WTM2
YOR230W	WTM1
YOR231W	MKK1
YOR234C	RPL33B
YOR237W	HES1
YOR238W	
YOR239W	ABP140
YOR240W	
YOR242C	SSP2
YOR243C	PUS7
YOR245C	DGA1
YOR247W	SRL1
YOR248W	
YOR251C	
YOR252W	TMA16
YOR253W	NAT5
YOR255W	OSW1
YOR258W	HNT3
YOR263C	
YOR264W	DSE3
YOR265W	RBL2
YOR266W	PNT1
YOR267C	HRK1
YOR268C	
YOR273C	TPO4
YOR277C	
YOR279C	RFM1
YOR280C	FSH3
YOR283W	
YOR284W	HUA2
YOR285W	
YOR286W	FMP31
YOR288C	MPD1
YOR289W	
YOR290C	SNF2
YOR291W	
YOR292C	
YOR293W	RPS10A
YOR296W	
YOR297C	TIM18
YOR298W	MUM3
YOR300W	
YOR301W	RAX1
YOR302W	
YOR303W	CPA1
YOR304W	ISW2
YOR306C	MCH5
YOR307C	SLY41
YOR308C	SNU66
YOR311C	HSD1
YOR312C	RPL20B
YOR313C	SPS4
YOR314W	
YOR315W	SFG1
YOR316C	COT1
YOR317W	FAA1
YOR318C	
YOR320C	GNT1
YOR321W	PMT3
YOR323C	PRO2
YOR324C	FRT1
YOR325W	
YOR327C	SNC2
YOR328W	PDR10
YOR331C	
YOR332W	VMA4
YOR333C	
YOR334W	MRS2
YOR337W	TEA1
YOR338W	
YOR339C	UBC11
YOR342C	
YOR343C	
YOR345C	
YOR346W	REV1
YOR347C	PYK2
YOR348C	PUT4
YOR350C	MNE1
YOR354C	MSC6
YOR356W	
YOR357C	SNX3
YOR359W	VTS1
YOR363C	PIP2
YOR365C	
YOR366W	
YOR367W	SCP1
YOR368W	RAD17
YOR369C	RPS12

YOR374W	ALD4
YOR377W	ATF1
YOR378W	
YOR379C	
YOR380W	RDR1
YOR381W	FRE3
YOR382W	FIT2
YOR383C	FIT3
YOR384W	FRE5
YOR385W	
YOR386W	PHR1
YPL001W	HAT1
YPL003W	ULA1
YPL004C	LSP1
YPL006W	NCR1
YPL008W	CHL1
YPL009C	
YPL013C	MRPS16
YPL014W	
YPL015C	HST2
YPL017C	IRC15
YPL018W	CTF19
YPL019C	VTC3
YPL021W	ECM23
YPL023C	MET12
YPL025C	
YPL026C	SKS1
YPL027W	SMA1
YPL030W	
YPL031C	PHO85
YPL032C	SVL3
YPL033C	
YPL034W	
YPL035C	
YPL036W	PMA2
YPL038W	MET31
YPL039W	
YPL040C	ISM1
YPL041C	
YPL045W	VPS16
YPL046C	ELC1
YPL048W	CAM1
YPL049C	DIG1
YPL050C	MNN9
YPL051W	ARL3
YPL052W	OAZ1
YPL053C	KTR6
YPL054W	LEE1
YPL055C	LGE1
YPL056C	
YPL057C	SUR1
YPL058C	PDR12
YPL059W	GRX5
YPL060W	LPE10
YPL064C	CWC27
YPL067C	
YPL068C	
YPL069C	BTS1
YPL070W	MUK1
YPL071C	
YPL072W	UBP16
YPL073C	
YPL074W	YTA6
YPL077C	
YPL078C	ATP4
YPL079W	RPL21B
YPL080C	
YPL081W	RPS9A
YPL084W	BRO1
YPL087W	YDC1
YPL088W	
YPL091W	GLR1
YPL092W	SSU1
YPL095C	EEB1
YPL096W	PNG1
YPL098C	MGR2
YPL100W	ATG21
YPL103C	FMP30
YPL105C	
YPL107W	
YPL108W	
YPL109C	
YPL110C	GDE1
YPL111W	CAR1
YPL112C	PEX25
YPL113C	
YPL114W	
YPL115C	BEM3
YPL116W	HOS3
YPL119C	DBP1
YPL121C	MEI5
YPL123C	RNY1
YPL127C	HHO1
YPL129W	TAF14
YPL130W	SPO19
YPL132W	COX11

YPL133C	RDS2
YPL134C	ODC1
YPL135W	ISU1
YPL136W	
YPL137C	GIP3
YPL139C	UME1
YPL140C	MKK2
YPL141C	
YPL145C	KES1
YPL147W	PXA1
YPL149W	ATG5
YPL154C	PEP4
YPL155C	KIP2
YPL156C	PRM4
YPL158C	
YPL159C	PET20
YPL162C	
YPL163C	SVS1
YPL164C	MLH3
YPL165C	SET6
YPL166W	ATG29
YPL167C	REV3
YPL168W	
YPL170W	DAP1
YPL171C	OYE3
YPL172C	COX10
YPL176C	TRE1
YPL177C	CUP9
YPL179W	PPQ1
YPL181W	CTI6
YPL182C	
YPL183C	
YPL183W-A	
YPL184C	MRN1
YPL185W	
YPL186C	UIP4
YPL187W	MF(ALPHA)1
YPL189W	GUP2
YPL191C	
YPL192C	PRM3
YPL193W	RSA1
YPL194W	DDC1
YPL196W	OXR1
YPL197C	
YPL198W	RPL7B
YPL199C	
YPL200W	CSM4
YPL201C	YIG1
YPL202C	AFT2
YPL203W	TPK2
YPL205C	
YPL206C	
YPL207W	TYW1
YPL212C	PUS1
YPL215W	CBP3
YPL216W	
YPL219W	PCL8
YPL220W	RPL1A
YPL221W	FLC1
YPL222W	FMP40
YPL223C	GRE1
YPL224C	MMT2
YPL225W	
YPL229W	
YPL230W	
YPL232W	SSO1
YPL234C	TFP3
YPL236C	
YPL239W	YAR1
YPL240C	HSP82
YPL241C	CIN2
YPL244C	HUT1
YPL246C	RBD2
YPL247C	
YPL248C	GAL4
YPL249C	GYP5
YPL256C	CLN2
YPL257W	
YPL258C	THI21
YPL259C	APM1
YPL260W	
YPL263C	KEL3
YPL264C	
YPL265W	DIP5
YPL267W	ACM1
YPL268W	PLC1
YPL272C	
YPL273W	SAM4
YPL274W	SAM3
YPR001W	CIT3
YPR002W	PDH1
YPR003C	
YPR004C	
YPR005C	HAL1
YPR006C	ICL2
YPR007C	REC8

YPR008W	HAA1
YPR009W	SUT2
YPR011C	
YPR012W	
YPR013C	
YPR014C	
YPR015C	
YPR017C	DSS4
YPR020W	ATP20
YPR021C	AGC1
YPR022C	
YPR023C	EAF3
YPR026W	ATH1
YPR027C	
YPR028W	YOP1
YPR029C	APL4
YPR030W	CSR2
YPR031W	NTO1
YPR032W	SRO7
YPR036W	VMA13
YPR037C	ERV2
YPR038W	IRC16
YPR039W	
YPR040W	TIP41
YPR042C	PUF2
YPR043W	RPL43A
YPR044C	OPI11
YPR045C	MNI2
YPR046W	MCM16
YPR049C	ATG11
YPR050C	
YPR052C	NHP6A
YPR053C	
YPR054W	SMK1
YPR058W	YMC1
YPR059C	
YPR061C	JID1
YPR062W	FCY1
YPR063C	
YPR068C	HOS1
YPR071W	
YPR072W	NOT5
YPR073C	LTP1
YPR075C	OPY2
YPR076W	
YPR077C	
YPR078C	
YPR079W	MRL1
YPR083W	MDM36
YPR084W	
YPR089W	
YPR090W	
YPR091C	
YPR092W	
YPR093C	ASR1
YPR095C	SYT1
YPR096C	
YPR097W	
YPR098C	
YPR109W	
YPR111W	DBF20
YPR114W	
YPR117W	
YPR118W	
YPR119W	CLB2
YPR120C	CLB5
YPR121W	THI22
YPR122W	AXL1
YPR123C	
YPR124W	CTR1
YPR125W	YLH47
YPR126C	
YPR127W	
YPR128C	ANT1
YPR129W	SCD6
YPR133W-A	TOM5
YPR134W	MSS18
YPR135W	CTF4
YPR138C	MEP3
YPR139C	VPS66
YPR140W	TAZ1
YPR145W	ASN1
YPR146C	
YPR147C	
YPR148C	
YPR149W	NCE102
YPR150W	
YPR151C	SUE1
YPR152C	URN1
YPR153W	
YPR154W	PIN3
YPR155C	NCA2
YPR156C	TPO3
YPR157W	
YPR158W	
YPR159W	KRE6

YPR160W	GPH1
YPR164W	MMS1
YPR167C	MET16
YPR170C	
YPR171W	BSP1
YPR172W	
YPR173C	VPS4
YPR174C	
YPR184W	GDB1
YPR185W	ATG13
YPR188C	MLC2
YPR191W	QCR2
YPR192W	AQY1
YPR193C	HPA2
YPR194C	OPT2
YPR195C	
YPR196W	
YPR197C	
YPR198W	SGE1
YPR199C	ARR1
YPR200C	ARR2
