# Supplementary material for: Genome-Wide Functional Profiling Identifies Genes and Processes Important for Zinc-Limited Growth of Saccharomyces cerevisiae
Source: PLoS Genet. 2012 Jun 7;8(6):e1002699. doi: 10.1371/journal.pgen.1002699 (PMC3369956; doi:10.1371/journal.pgen.1002699)
Supplement: Table S4 — Autophagy is not greatly required for low iron or low copper growth. Various autophagy mutants showing growth defects in low zinc were cultured in metal replete (YPD) and low iron (YPD+100 µM BPS) or low copper (YPD+200 µM BCS) and assayed for growth as described for Table 1. (PDF) [file pgen.1002699.s005.pdf]

Supplemental Table 4. Autophagy is not greatly required for low iron or low copper growth.

Strain	% mutant in initial inoculum	% mutant after 15 gen. in YPD	% mutant after 15 gen. + BPS	% mutant after 15 gen. + BCS	+BPS/-BPS ratio	p-value[a]	+BCS/-BCS ratio	p-value[a]
fet3Δ	51.60	53.50 ± 0.69	2.35 ± 0.64	53.50 ± 0.01	0.04	0.0002	1.0	NS
irc21Δ	42.50	33.37 ± 1.46	41.17 ± 5.98	5.31 ± 0.23	1.2	NS	0.2	0.0008
atg3Δ	38.10	38.40 ± 0.10	54.47 ± 1.62	33.67 ± 0.64	1.4	0.004	0.9	0.006
atg8Δ	53.20	50.33 ± 0.50	54.50 ± 0.20	47.97 ± 0.51	1.1	0.007	0.9	0.007
atg15Δ	46.20	23.00 ± 0.17	67.83 ± 2.97	22.03 ± 0.84	2.9	0.002	1.0	NS
atg16Δ	31.00	37.47 ± 0.12	46.80 ± 1.77	35.50 ± 0.26	1.2	0.01	1.0	NS

[a] Significance was defined as having a p-value less than 0.05; NS = not significant.
