# Supplementary material for: Genome-Wide Functional Profiling Identifies Genes and Processes Important for Zinc-Limited Growth of Saccharomyces cerevisiae
Source: PLoS Genet. 2012 Jun 7;8(6):e1002699. doi: 10.1371/journal.pgen.1002699 (PMC3369956; doi:10.1371/journal.pgen.1002699)
Supplement: Table S5 — Effects of mutations in autophagy and ER stress response on growth of iron- or copper-limited cells that are zinc replete. Various autophagy mutants and mutants disrupting ER function were cultured in metal replete, low iron, and low copper conditions and assayed for growth as described for Table 1. LZM was made iron-limiting by leaving out the iron supplement and adding 100 µM ZnCl2 to make it zinc replete. Iron was then supplemented to either 1 (low iron) or 100 (replete iron) µM FeCl3. LZM was modified to control copper availability by leaving out the copper supplement and replacing the EDTA with 200 µM BCS. Copper was then supplemented to either 0.1 (low copper) or 10 (replete copper) µM CuCl2. (PDF) [file pgen.1002699.s006.pdf]

Supplemental Table 5. Effects of mutations in autophagy and ER stress response on growth of iron- or copper-limited cells that are zinc replete.

Strain	% mutant in initial inoculum	% mutant after 15 gen. in low Fe	% mutant after 15 gen. in replete Fe	-Fe/+Fe ratio	p-value[a]	% mutant after 15 gen. in low Cu	% mutant after 15 gen. in replete Cu	-Cu/+Cu ratio	p-value[a]
BY4743	50.70	49.23 ± 0.31	49.37 ± 0.30	1.0	NS	47.97 ± 0.33	52.50 ± 0.49	0.9	0.006
fet3Δ	50.10	0.90 ± 0.23	15.23 ± 0.12	16.7	0.0001	--	--	--	--
irc21Δ	49.30	--	--	--	--	4.32 ± 0.26	44.10 ± 0.11	10.2	0.0001
atg3Δ	45.10	46.90 ± 0.80	35.33 ± 0.45	1.3	0.001	34.90 ± 0.70	43.27 ± 0.60	0.8	0.001
atg8Δ	49.10	46.73 ± 0.23	42.03 ± 0.52	1.1	0.007	40.43 ±0.23	50.23 ± 0.27	0.8	0.001
atg15Δ	46.40	26.97 ± 0.40	30.13 ± 0.77	0.9	0.01	24.20 ± 1.0	16.70 ± 0.20	1.5	0.007
atg16Δ	44.00	43.27 ± 0.58	44.83 ± 0.68	1.0	NS	46.97 ± 0.19	50.10 ± 0.33	0.9	0.003
hac1Δ	49.70	52.77 ± 0.23	49.50 ± 0.49	1.1	0.003	48.03 ± 0.50	42.53 ± 0.35	1.1	0.007
ire1Δ	49.50	52.17 ± 0.91	48.87 ± 0.24	1.1	0.02	46.67 ± 0.46	42.43 ± 0.20	1.1	0.002
ice2Δ	49.50	38.90 ± 0.36	27.83 ± 0.74	1.4	0.002	27.87 ± 0.64	12.53 ± 0.33	2.2	0.001

[a] Significance was defined as having a p-value less than 0.05; NS = not significant.
