# Supplementary material for: Genome-Wide Functional Profiling Identifies Genes and Processes Important for Zinc-Limited Growth of Saccharomyces cerevisiae
Source: PLoS Genet. 2012 Jun 7;8(6):e1002699. doi: 10.1371/journal.pgen.1002699 (PMC3369956; doi:10.1371/journal.pgen.1002699)
Supplement: Table S6 — Enrichment among low zinc-resistant mutants for molecular function, biological processes, and other categories. (PDF) [file pgen.1002699.s007.pdf]

Supplemental Table 6. Enrichment among low zinc-resistant mutants for molecular function, biological processes, and other categories.

GO Molecular Function category	p-value	Genes identified	k[a]	f[b]
Structural constituent of ribosome [GO:0003735]	1.00E-14	*MRPL16 MRP21 RPS11B RPS6B MRPL37 RPS14A RPP1A RPL13A RPS16B RPL35B MRPL11 RPS11A YDR115W RSM24 MRPL7 MRPL35 MRPS28 MRP1 MRP20 RPS17B RPS18A MRPL28 RML2 RPL12A RSM18 RPS24A RPL24A RPL7A RSM23 RPL1B RPL9A MRPL25 RPS23A MRPS35 RPS0A RSM27 MRPL9 RPL27A MRPL6 RPS4B RPL2B RPS24B RSM25 RPL16A MRPL8 RPS21B RPS4A MRP17 MRPL31 MRPL38 RPS21A MRPL20 RPS0B RPL22A RPL37A RPS28B RPS30A RPS29A MRPL4 RPL6B RPS17A RPS18B RPL6A RPL13B MRPS17 MRPL24 RPS10B MRP7 RPL16B RPS7B MRPL22 MRPL17 MRPL10 MRPS12 RPS19A RPS7A MRPL23 RPS28A RPS30B RPS6A MRP51 MRPL40 MRPL51 RPS23B MRP2*	85	417
rRNA binding [GO:0019843]	2.46E-09	*MRPL16 RPS11B RPS11A MRPS28 EFT2 RPS18A RPL9A CCM1 SSF1 MRPL6 RPS4B RPL2B RPS4A MRP17 RPL37A HCR1 RPS18B MRPS17*	18	46

GO Biological Process category	p-value	Genes identified	k[a]	f[b]
Mitochondrial translation [GO:0032543]	1.00E-14	*MRPL16 PET112 MRP21 MRPL37 MRPL11 YDR115W RSM24 MRPL7 MRPL35 MRPS28 MRP1 MRP20 MRPL28 RSM18 RSM23 MRF1 MRPL25 MRPS35 RSM27 RRF1 MRPL6 RSM25 MRPL8 MRP17 MRPL31 MRPL38 MRPL20 MEF1 SLS1 NAM2 MRPL4 MTG1 MRPS17 MRPL24 MRP7 MRPL22 MRPL17 MRPL10 MRPS12 IFM1 MRPL23 MRP51 MRPL40 MRPL51 MRP2*	45	104
Translation [GO:0006412]	1.00E-14	*MRPL16 PET112 MRP21 RPS11B RPS6B SRO9 RPS14A MTF2 RPP1A RPL13A RPS16B RPL35B RPS11A YDR115W MRPL7 MSW1 MRPS28 EFT2 MRP20 RPS17B RPS18A RML2 RPL12A RSM18 RPS24A AIM10 RPL24A RPL7A RPL1B MRF1 RPL9A YGR102C RPS23A TIF4631 RPS0A MRPL9 RPL27A DIA4 RRF1 SSZ1 MRPL6 RPS4B RPL2B RPS24B RSM25 RPL16A MRPL8 MEF2 RPS21B RPS4A MRP17 MRPL38 GCN3 RPS21A HBS1 RPS0B RPL22A MEF1 SLS1 RPL37A HCR1 RPS28B RPS30A GUF1 NAM2 RPS29A MRPL4 RPL6B RPS17A RPS18B RPL6A AEP1 RPL13B MRPS17 MRPL24 RPS10B HER2 MRP7 RPL16B RPS7B MRPL22 MRPL10 MRPS12 IFM1 RPS19A SKI7 RPS7A MRPL23 RPS28A RPS30B RPS6A MSY1 MSD1 MRPL40 MSF1 RPS23B MRP2*	97	517
Mitochondrial genome maintenance [GO:0000002]	4.16E-11	*EXO5 RIM1 GGC1 RRG1 MHR1 MDJ1 RPO41 CCM1 MSH1 MRPL8 ILM1 MMM1 GEP5 PIF1 ABF2 YHM2 HMI1 MGM1 RRG8*	19	42
Ribosomal small subunit assembly [GO:0000028]	6.18E-07	*DRS2 RPS11B RPS14A RPS11A RPS17B NSR1 RPS0A RPS0B RPS17A*	9	16
Mitochondrial translational initiation [GO:0070124]	2.65E-06	*MRP21 AEP2 IFM1 AEP3 MRP51*	5	5

GO Cellular Component category	p-value	Genes identified	k[a]	f[b]
Mitochondrial large ribosomal subunit [GO:0005762]	1.00E-14	*MRPL16 MRPL37 MRPL11 YDR115W MRPL7 MRPL35 MRP20 MRPL28 RML2 MRPL25 MRPL9 MRPL6 MRPL8 MRPL31 MRPL38 MRPL20 MRPL4 MRPL24 MRP7 MRPL22 MRPL17 MRPL10 MRPL23 MRPL40 MRPL51*	25	53
Cytosolic small ribosomal subunit [GO:0022627]	1.00E-14	*RPS11B RPS6B RPS14A PAT1 RPS16B RPS11A RPS17B RPS18A RPS24A RPS23A RPS0A RPS4B RPS24B RPS21B RPS4A RPS21A RPS0B RPS28B RPS30A RPS29A RPS17A RPS18B ASC1 RPS10B RPS7B RPS19A RPS7A RPS28A RPS30B RPS6A RPS23B*	31	72
Ribonucleoprotein complex [GO:0030529]	1.00E-14	*MRPL16 MRP21 RPS11B RPS6B MRPL37 RPS14A RPP1A RPL13A RPS16B RPL35B MRPL11 RPS11A YDR115W RSM24 MRPL7 MRPL35 MRPS28 MRP1 MRP20 RPS17B RPS18A MRPL28 RML2 RPL12A RSM18 RPS24A RPL24A RPL7A RSM23 RPL1B RPL9A MRPL25 RPS23A MRPS35 RPS0A RSM27 MRPL9 RPL27A MRPL6 RPS4B RPL2B RPS24B RSM25 RPL16A MRPL8 LSM1 RPS21B RPS4A MRP17 MRPL31 MRPL38 RPS21A MRPL20 RPS0B RPL22A RPL37A RPS28B RPS30A RPS29A MRPL4 RPL6B RPS17A RPS18B RPL6A RPL13B MRPS17 MRPL24 RPS10B MRP7 RPL16B RPS7B MRPL22 MRPL17 MRPL10 MRPS12 RPS19A BUD21 RPS7A MRPL23 RPS28A RPS30B RPS6A MRP51 MRPL40 MRPL51 RPS23B MRP2*	87	312
Intracellular [GO:0005622]	1.00E-14	*MRPL16 MRP21 RPS11B RPS6B REI1 RPS14A RPN4 RPP1A RPL13A RPS16B RPL35B MRPL11 RPS11A VMS1 YDR115W MRPL7 MRPS28 MRP20 RPS17B RPS18A STP1 RML2 RSM18 RPS24A MIG1 RPL7A RPL1B RPL9A RPS23A RPS0A MRPL9 RIM101 RPL27A MRPL6 RPS4B RPL2B RPS24B MRPL8 RPS21B RPS4A MRT4 RPS21A IRC25 RPS0B RPL22A MEF1 BUD20 ACF2 RPL37A RPS28B RPS30A RPS29A SFP1 RPL6B RPS17A RPS18B RPL6A MIH1 MTG1 RPL13B RIM13 MRPS17 MRPL24 MRP7 RPS7B MRPL22 JJJ1 MRPS12 IFM1 RPS19A RRP6 RPS7A RPS28A RPS30B RPS6A POC4 MRPL40 RPS23B MRP2*	79	389
Ribosome [GO:0005840]	1.00E-14	*MRPL16 MRP21 RPS11B RPS6B MRPL37 RPS14A RPP1A RPL13A RPS16B RPL35B MRPL11 RPS11A YDR115W RSM24 MRPL7 MRPL35 MRPS28 MRP1 EFT2 MRP20 RPS17B RPS18A MRPL28 RML2 RPL12A RSM18 RPS24A RPL24A RPL7A RSM23 RPL1B RPL9A MRPL25 RPS23A TIF4631 MRPS35 RPS0A RSM27 MRPL9 ZUO1 RPL27A MRPL6 RPS4B RPL2B RPS24B RSM25 RPL16A MRPL8 RPS21B RPS4A MRP17 MRPL31 MRPL38 RPS21A MRPL20 RPS0B RPL22A RPL37A RPS28B RPS30A RPS29A MRPL4 RPL6B RPS17A RPS18B RPL6A RPL13B MRPS17 MRPL24 RPS10B MRP7 RPL16B RPS7B MRPL22 MRPL17 MRPL10 MRPS12 RPS19A RPS7A MRPL23 RPS28A RPS30B RPS6A MRP51 MRPL40 MRPL51 RPS23B MRP2*	88	485
Mitochondrial small ribosomal subunit [GO:0005763]	3.67E-07	*MRP21 RSM24 MRPS28 MRP1 RSM18 RSM23 MRPS35 RSM27 RSM25 MRP17 MRPS17 MRPS12 MRP51 MRP2*	14	38

MIPS Functional Classification category	p-value	Genes identified	k[a]	f[b]
Ribosomal proteins [12.01.01]	1.00E-14	MRPL16 MRP21 RPS11B RPS6B MRPL37 RPS14A RPP1A RPL13A RPS16B RPL35B MRPL11 RPS11A YDR115W RSM24 MRPL7 MRPL35 MRPS28 MRP1 MRP20 RPS17B RPS18A MRPL28 RML2 RPL12A RSM18 RPS24A RPL24A RPL7A RSM23 RPL1B RPL9A MRPL25 RPS23A NSR1 MRPS35 RPS0A RSM27 MRPL9 RPL27A PIH1 SSF1 MRPL6 RPS4B RPL2B RPS24B RSM25 RPL16A MRPL8 RPS21B RPS4A MRP17 MRPL31 MRPL38 DBP7 RPS21A MRPL20 RPS0B RPL22A RPL37A RPS28B RPS30A RPS29A MRPL4 RPL6B RPS17A RPS18B RPL6A RPL13B MRPS17 MRPL24 RPS10B MRP7 RPL16B RPS7B MRPL22 MRPL17 MRPL10 MRPS12 RPS19A RPS7A MRPL23 RPS28A RPS30B RPS6A MRP51 MRPL40 MRPL51 RPS23B MRP2	89	246

Supplemental Table 6. Enrichment among low zinc-resistant mutants for molecular function, biological processes, and other categories.

Mitochondrion [42.16]	1.00E-14	MRPL16 PET112 MRP21 MRPL37 GGC1 MRPL11 YDR115W RSM24 MSS116 MRPL7 MRPL35 MRPS28 MRP1 SHE9 MRP20 MRPL28 RML2 RSM18 RPO41 MRH4 RSM23 MRM2 MDM34 MRPL25 PCP1 MRPS35 RSM27 MRPL9 MRPL6 MDM31 FIS1 RSM25 MRPL8 MRP17 MRPL31 MRPL38 MRPL20 MMM1 MDM30 MRPL4 ABF2 MRPS17 MRPL24 MTF1 MRP7 POR1 MRPL22 MRPL17 MRPL10 MRPS12 MDM12 MRPL23 MGM1 MRP51 MRPL40 YME1 MRPL51 MRP2	58	170
Aerobic respiration [02.13.03]	8.49E-07	PET112 ETR1 YDR115W RSM24 MRPS35 DIA4 COX6 COX23 SLS1 COQ9 MRPS17 PPA2 HER2 POR1 MRPL22 CYT1 MCT1 GDS1 MDL2 MRPL51	20	77
**MIPS Phenotypes category**	**p-value**	**Genes identified**	**k[a]**	**f[b]**
Respiratory deficiency [42.25.20]	1.00E-14	*PET112 TCM62 MRPL11 ATP5 MRPS28 MRP1 ATP17 MRP20 RML2 AFG3 PET117 COX15 PET122 OXA1 MDJ1 RRF1 MSH1 MRPL6 EGD2 PET130 MRPL8 NUC1 PET191 GEF1 OCT1 MMM1 SLS1 SSQ1 NAM2 MRPL4 ABF2 YTA12 MTF1 YHM2 AEP2 DSS1 PET8 MRP7 POR1 PHO80 CAT5 IDH2 LIP5 MRM1 MCT1 MIP1 GDS1 HAP5 SUV3 MSY1 MSD1 MRP51 PPT2 ATP15 YME1 MAK3 MRP2*	57	173
Slow-growth (slg) [12.15]	6.74E-08	*ATS1 FEN2 BUD23 BUD31 PAT1 HMO1 REF2 VAC8 RPL7A ARC1 RPL1B RPS0A PRS3 RPL27A RRF1 COX6 FYV4 SSZ1 HTD2 MSH1 CPR7 DBP7 RPS0B YKE2 RPS30A RSC2 SSQ1 SFP1 AMD1 GTR1 ASC1 RIM13 SKY1 UBP8 VAC7 TPM1 IES2 MRPL17 ALD6 ELP3 SSE1 LEA1 MAK3*	43	237
Mitochondrial mutants [52.55]	1.81E-06	*ETR1 MRPS28 MRP1 RML2 RPO41 PET191 MMM1 SSQ1 DSS1 TPM1 MGM1 YME1 MRP2*	13	37
**MIPS Subcellular Localization category**	**p-value**	**Genes identified**	**k[a]**	**f[b]**
Mitochondrial matrix [755.07]	9.37E-14	*GCV3 MRPL16 PET112 MRP21 PDB1 MRPL37 RIM1 MRPL11 YDR115W MSS116 MHR1 MRPL28 RSM18 LPD1 MRM2 RSM27 RSM25 NFU1 ISA1 LIP2 ABF2 MRPS17 MRPL22 HMI1 MRP51 MSF1 ISA2 MRPL51*	28	72
Mitochondrial inner membrane [755.05]	4.78E-05	*TCM62 GGC1 COQ4 ATP5 ATP22 SHE9 AFG3 COX15 PET122 OXA1 MDJ1 PCP1 MTM1 COX6 YIA6 MRS3 NUC1 ATP7 COQ9 YTA12 YHM2 PET8 CYT1 CAT5 MDL2 ATP15 YME1*	27	156
**MIPS Protein Complexes category**	**p-value**	**Genes identified**	**k[a]**	**f[b]**
Mitochondrial ribosomal large subunit [500.60.10]	1.00E-14	*MRPL16 MRPL37 MRPL11 YDR115W MRPL7 MRPL35 MRP20 MRPL28 RML2 MRPL25 MRPL9 MRPL6 MRPL8 MRPL31 MRPL38 MRPL20 MRPL4 MRPL24 MRP7 MRPL22 MRPL17 MRPL10 MRPL23 MRPL40 MRPL51*	25	44
Cytoplasmic ribosomal small subunit [500.40.20]	1.00E-14	*RPS11B RPS6B RPS14A RPS16B RPS11A RPS17B RPS18A RPS24A RPS23A RPS0A RPS4B RPS24B RPS21B RPS4A RPS21A RPS0B RPS28B RPS30A RPS29A RPS17A RPS18B ASC1 RPS10B RPS7B RPS19A RPS7A RPS28A RPS30B RPS6A RPS23B*	30	57
Complex Number 108, probably protein synthesis turnover [550.1.108]	1.28E-10	*MRPL16 YDR115W MRPL7 MHR1 MRPL35 MRP20 MRPL28 MRH4 MRPL25 MRPL9 MRPL6 MRPL8 MRPL20 MRPL4 MRPL24 MRP7 MRPL17 MRPL10 MRPL23 MRM1 MRPL51*	21	54
Mitochondrial ribosomal small subunit [500.60.20]	1.61E-07	*MRP21 RSM24 MRPS28 MRP1 RSM18 RSM23 MRPS35 RSM27 RSM25 MRP17 MRPS17 MRP51 MRP2*	13	31
Complex Number 104, probably protein synthesis turnover [550.1.104]	7.62E-07	*MRP21 RSM24 MRPS28 MRP1 RSM23 CCM1 TIF4631 RSM27 FYV4 RSM25 MRP17 MRPS17 GEP3 MRP51*	14	40

[a] k = number of genes of specific category identified by screen that when deleted increase tolerance to low zinc

[b] f = total number of genes in specific GO / MIPS category
