# Supplementary material for: Genome-Wide Functional Profiling Identifies Genes and Processes Important for Zinc-Limited Growth of Saccharomyces cerevisiae
Source: PLoS Genet. 2012 Jun 7;8(6):e1002699. doi: 10.1371/journal.pgen.1002699 (PMC3369956; doi:10.1371/journal.pgen.1002699)
Supplement: Table S7 — Zinc-regulated genes whose mutants were sensitive to low zinc. Deletion mutants identified in the functional profiling analysis as sensitive to low zinc and also known from other studies to be induced in low zinc are listed. (PDF) [file pgen.1002699.s008.pdf]

Supplemental Table 7. Zinc-regulated genes whose mutants were sensitive to low zinc.

Gene	Zap1 regulated?	Function
HTA1	No	Histone 2A
HAC1	No	ER stress-responsive transcription factor
ADH4	Yes	Alcohol dehydrogenase isozyme, Zap1 target gene
NAB6	No	Putative RNA-binding protein
UTH1	Yes	Implicated in oxidative stress resistance, mitophagy, and cell wall biogenesis
ROT2	No	Glucosidase II catalytic subunit required for normal cell wall synthesis
ZRG17	Yes	Subunit of the Msc2/Zrg17 endoplasmic reticulum zinc uptake transporter
ADP1	No	Putative ATP-dependent permease of the ABC transporter family of proteins
HNT1	Yes	Adenosine 5'-monophosphoramidase of unknown function
DFG5	No	Putative mannosidase, cell surface protein required for cell wall biogenesis
UBI4	No	Ubiquitin
ICY2	Yes	Protein of unknown function
TSA1	Yes	Thioredoxin peroxidase required for oxidative stress resistance
OPI3	No	Phospholipid methyltransferase, phosphatidylcholine biosynthesis
ARG4	No	Argininosuccinate lyase, arginine biosynthesis
